# Theoretical study of the molecular aspect of the suspected novichok agent A234 of the Skripal poisoning

**DOI:** 10.1098/rsos.181831

**Published:** 2019-02-06

**Authors:** Hanusha Bhakhoa, Lydia Rhyman, Ponnadurai Ramasami

**Affiliations:** 1 Computational Chemistry Group, Department of Chemistry, Faculty of Science, University of Mauritius, Réduit 80837, Mauritius; 2 Department of Applied Chemistry, University of Johannesburg, Doornfontein, Johannesburg 2028, South Africa

**Keywords:** chemical warfare, DFT, nerve agent, novichok, A234, sarin

## Abstract

Novichoks are the suspected nerve agents in the March 2018 Skripal poisoning. In this context, the novichok agent A234 (chemical structure proposed by Mirzayanov) was studied using computational methods to shed light on its molecular, electronic, spectroscopic, thermodynamic and toxicity parameters as well as on potential thermal and hydrolysis degradation pathways. The poisoning action and antidote of A234 were also investigated. Some of these parameters were compared to three common G- and V-series nerve agents, namely GB, VR and VX. The research findings should be useful towards the detection, development of antidotes and destruction of A234.

## 1. Introduction

Nerve agents, organophosphate containing chemical warfare agents, are among the most toxic chemicals known to mankind [1]. They can inactivate acetylcholinesterase (AChE) which is a key central nervous system (CNS) enzyme responsible for the breakdown of the neurotransmitter acetylcholine; thus, leading to rapid and severe adverse effects on the environment, human and animals [2,3]. Several nerve agents of the G- [tabun (GA), sarin (GB), chlorosarin (GC) and soman (GD)] and V-series (VE, VR, VS and VX) have been deployed not only in warfare but also in acts of terrorism and high-profile assassinations [4,5]. For instance, GB was used in chemical attacks in the Syrian civil war between 2013 and 2017, while VX was employed to murder the half-brother of the North Korean leader Kim Jong-un at Kuala Lumpur Airport in February 2017. Such events are indicative that these nerve agents are still a threat to the international community, despite the fact that their use is being regulated by the Organisation for the Prohibition of Chemical Weapons under the Chemical Weapons Convention (CWC) [6].

**Scheme 1.**
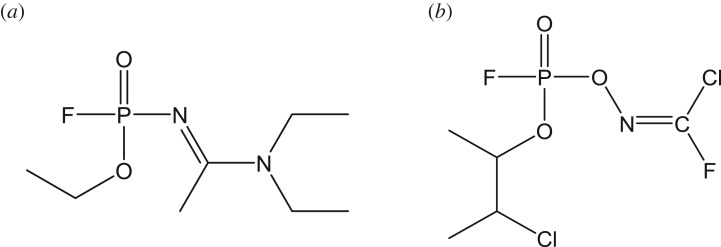
Chemical structures of novichok A234 as proposed by Mirzayanov [14] (*a*) as well as Hoenig [15] and Ellison [16] (*b*).

A novel class of nerve agents, the novichoks or the A-series, has recently come into the limelight following the March 2018 assassination attempt on the former Russian spy, Sergei Skripal and his daughter Yulia in Salisbury, UK [7–9]. Almost 30 years have passed since these ‘fourth generation’ nerve agents were developed by the Soviet Union in a Cold War-era weapons programme [10]. However, information on novichoks is still guarded as ‘top secret’ and exact reliable data are missing. The Skripal poisoning has catalysed the pursuit of detailed information on the history, chemical structure, synthesis, toxicity, deployment, detection and destruction of novichoks. This yielded some reports which are insightful although incomplete at molecular level [4,7–9,11–13]. One of the concerns is related to their chemical structure. For example, there is still a debate as to whether a novichok agent denoted as A234 (scheme 1) corresponds to either structure (*a*) as proposed by Mirzayanov [14] or structure (*b*) as proposed by Hoenig [15] and Ellison [16].

To the best of our knowledge, there is only one scientific publication that provides experimental data for a chemical (alkyl-*N*-[bis(dimethylamino)methylidene]-*P*-methylphosphonamidates) that is specifically identified as a novichok and this compound is included in the Schedule 2.B.04 of the CWC [17]. We are reporting a theoretical study on novichok and in particular on the novichok agent A234 (structure (*a*) of scheme 1; *N*'-[ethoxy(fluoro)phosphoryl]-*N*,*N*-diethylethanimidamide) because some open sources [18–20] speculated the use of A234 in the absence of official communications on the exact chemical identified from the UK incident. Henceforth, A234 will correspond to structure (*a*) of scheme 1 throughout this paper. It is noteworthy that A234 has not yet been scheduled under the CWC and at the revision stage of this manuscript, two reports on novichoks pertaining to QSAR [21] and molecular docking approaches [22] appeared.

This study aims at providing the molecular, electronic, spectroscopic, thermodynamic and toxicity parameters of A234 together with plausible thermal and hydrolysis degradation pathways. The poisoning action and antidote of A234 were also investigated. Some of these parameters are compared to three common G- and V-series nerve agents, namely GB (2-[fluoro(methyl)phosphoryl]oxypropane), VR (*N*,*N*-diethyl-2-[methyl(2-methylpropoxy)phosphoryl]sulfanylethanamine) and VX (*N*-[2-[ethoxy(methyl)phosphoryl]sulfanylethyl]-*N*-propan-2-ylpropan-2-amine).

## 2. Methodology

Conformational analysis for A234, VR and VX was carried out using the MMFF94s force field as implemented in the CONFLEX software [23–25]. The conformational search was limited to 5 kcal mol^−1^ and this led to 115, 8061 and 2473 conformers of A234, VR and VX, respectively. The optimization of the 115 conformers of A234 using the B3LYP/6-311++G(d,p) [26–28] method converged to 26 conformers; their optimized structures together with their relative energies are provided in electronic supplementary material, figure S1. The first 125 conformers of VR and VX were also optimized using the B3LYP/6–311++G(d,p) and the relative energies and Cartesian coordinates of their resulting first 15 minimum energy structures are provided in the electronic supplementary material. Previously reported [29] minimum energy structures of GB were herein revisited using the B3LYP/6-311++G(d,p) method. The B3LYP lowest minimum energy structures of A234, GB, VR and VX were also optimized using the M06-2X/6-311++G(d,p) [30] and MP2/6-311++G(d,p) [31] methods. The B3LYP [29,32,33] and M06-2X [34,35] DFT functionals and the MP2 [29,36] method in conjunction with different Pople basis sets have been used to study nerve agents and related organophosphorus species.

Geometry optimization was followed by analytic Hessian computation using the same methods. The absence of negative Hessian eigenvalues confirmed the stationary points as minima on the potential energy hypersurfaces. Zero-point energy correction was included in the relative energies (Δ*E*s). Reported energies are given at 298.15 K and 1 atm. Natural bond orbital (NBO) analysis was also carried out using the M06-2X/6-311++G(d,p) method [37]. Computations were performed by means of resources provided (Gaussian 16 [38] package) by SEAGrid [39–42].^1^

Geometry optimization was also conducted in solvents (water and *n*-octanol) using the M06-2X/6-311++G(d,p) method in view to calculate the lipophilicity of A234, GB, VR and VX. Solvent effect was taken into account based on the polarizable continuum model [43]. The classical descriptor for lipophilicity is the log *P*_o/w_ (partition coefficient between *n*-octanol and water) [44]. The log *P*_o/w_ values were obtained using the following equation:

2.1 $$\log\;P_{o/w}=\frac{-\Delta G}{2.303RT}$$

where *R* is the universal gas constant (8.314 J K^−1^ mol^−1^), *T* is the system temperature (298.15 K) and Δ*G* (in J mol^−1^) is the difference between the solvation absolute Gibbs free energies in water (*G*_water_) and in *n*-octanol (*G*_n-octanol_). The log *P*_o/w_ values for the four nerve agents were also calculated via the SwissADME [44] and the ALOGPS 2.1 program [45].

The optimized structures in water were used for computing nuclear magnetic resonance (NMR) chemical shifts with the gauge-including atomic orbital method [46] using shieldings of trimethylsilane (for ^1^H NMR and ^13^C NMR), nitromethane (for ^15^N NMR), phosphoric acid (for ^31^P NMR) and trichlorofluoromethane (for ^19^F NMR) computed using the M06-2X/6-311++G(d,p) method.

The CBS-QB3 composite method [47] was employed to calculate the enthalpy of formation ($\Delta_{f}H_{298}^{\circ }$) of the nerve agents. In the CBS-QB3 model, the geometry and frequency computations were performed using the B3LYP/6-311G(2d,d,p) method (also denoted as cbsb7). The CBS-QB3 model is known to perform well and predict accurate energies for organophosphorus compounds [48,49]. The $\Delta_{f}H_{298}^{\circ }$ of A234 was further employed to determine its bond dissociation energies (BDEs) as per the following process RR′ → R• + R'• (where RR′ and R•/R'• represent A234 and the individual radical fragments, respectively) to provide an insight into favourable thermal decomposition pathways [48]. The BDE corresponds to the enthalpy of reaction ($\Delta_{r}H_{298}^{\circ }$) of the thermal dissociation process, where

2.2 $$\mathrm{BDE}=\;\Delta_{r}H_{298}^{\circ }=[\Delta_{f}H_{298}^{\circ }(R•)+\Delta_{f}H_{298}^{\circ }(R^{\prime }•)]-\Delta_{f}H_{298}^{\circ }(RR^{\prime }).$$

The $\Delta_{f}H_{298}^{\circ }$ of each radical was determined using the CBS-QB3 method.

## 3. Results and discussion

Section 3.1 consists of the minimum energy structures of A234, GB, VR and VX. Their spectroscopic parameters, conceptual DFT-based reactivity descriptors, molecular electrostatic potential (MEP) and ADME (absorption, distribution, metabolism, excretion) properties are discussed in §§3.2–3.5, respectively. Their poisoning action and possible antidotes based on model reactions are reported in §3.6. The hydrolysis and thermal degradation of A234 are discussed in §§3.7 and 3.8, respectively.

### 3.1. Structural parameters

**Figure 1.**
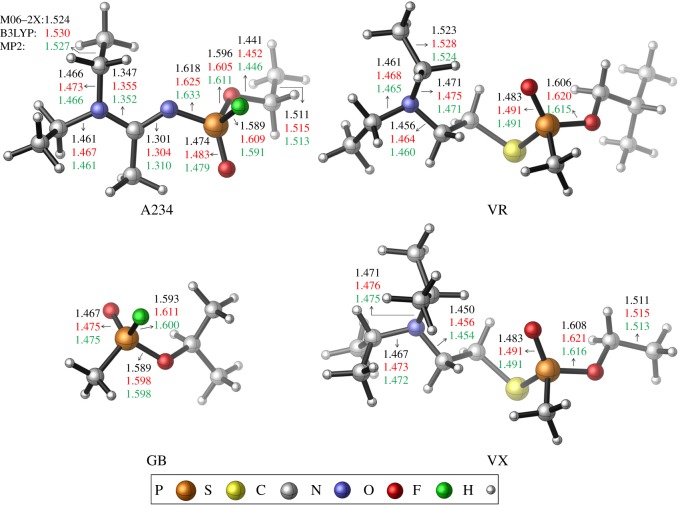
Optimized structures of the nerve agents with selected bond lengths (Å).

Selected bond lengths of the gas-phase optimized structures of A234, GB, VR and VX are provided in figure 1 and additional details are collected in electronic supplementary material, table S1. The rotational constants are tabulated in electronic supplementary material, table S2. The root-mean-square deviation (RMSD) between the atomic positions of the M06-2X and B3LYP optimized structures are 0.1390 and that of the M06-2X and MP2 optimized structures are 0.7747. The high RMSD value arises due to a rotation about the N–P bond in the MP2 optimized structure. A234 has structural difference with GB, VR and VX. The P=O centre of A234 is attached to an acetoamidine group (which consists of an active –N=CR–N< framework) along with –OR and –F.

### 3.2. Spectroscopic analysis

**Figure 2.**
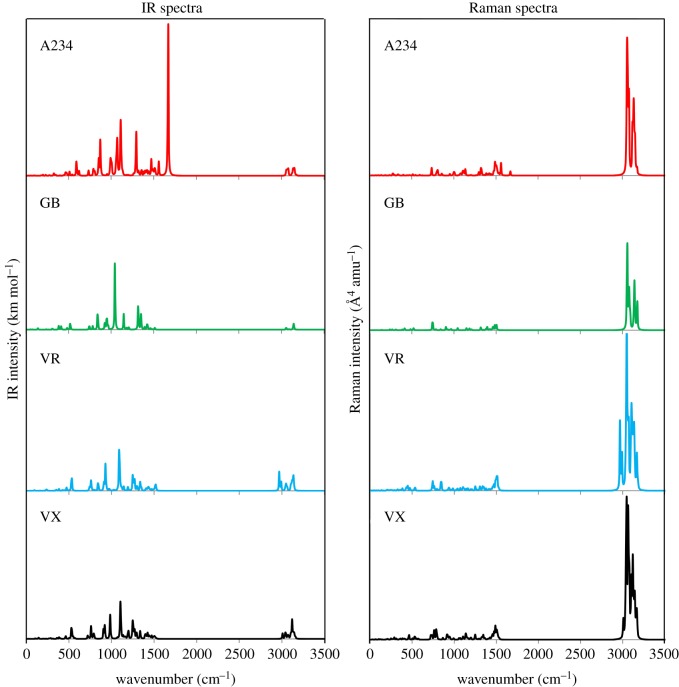
Simulated IR and Raman spectra of the nerve agents.

The IR and Raman spectra of GB, VR and VX were revisited and those of A234 are reported based on the M06-2X/6-311++G(d,p) method in the gas phase (figure 2 and table 1). The IR and Raman spectra of the nerve agents consist of two distinct regions, notably a low-wavenumber region at 200–1700 cm^−1^ and a high-wavenumber region at 2950–3200 cm^−1^. The high-wavenumber region consists of C–H stretching vibrations which are weakly IR active (A234: 3052–3173 cm^−1^; GB: 3053–3176 cm^−1^; VR: 2965–3179 cm^−1^; VX: 3006–3172 cm^−1^). This particular region is highly Raman active with broad absorption bands. The low-wavenumber region (fingerprint region comprising several vibrational modes) of each nerve agent differs from each other. The low-wavenumber region features weakly to non-Raman active absorption bands. A sharp and distinct peak due to C=N stretching at 1670 cm^−1^ is noticeable in the IR spectra of A234. This peak is characteristic for substituted amidines which absorb strongly at 1600–1700 cm^−1^ [53]. The C=N stretching of an *N*-phosphorylated alkylisourea of the type (EtO)_2_P(O)NC(OEt)N(CH_2_CH=CH_2_)_2_ has been reported at 1640 cm^−1^ and this correlates with that of A234 [50]. The C–N stretching of the –N=CR–N< acetoamidine unit of A234 is of weaker intensity and appears at a lower wavenumber (1560 cm^–1^) than that of C=N stretching. Literature values indicate that the C–N bond of amidine derivatives vibrates at lower frequencies within a range of 1230–1412 cm^−1^ [54–56]. The peak of highest intensity within the low-wavenumber region corresponds to the O−C stretching for A234, VR and VX (1113, 1091 and 1107 cm^−1^, respectively) and P–O–C asymmetric stretching for GB (1045 cm^−1^).

**Table 1.** Assignment of selected vibrational modes of the nerve agents.

vibrational modes	wavenumber (cm^−1^)				
	A234	GB	VR	VX	literature (exp.)
*υ*(P=O)	1266, 1294	1319, 1352	1251, 1269	1246, 1269	1255 [50], 1278 [51], 1227 [51], 1317 [52]
*υ*(O−C)	1113	1148	1091	1107	—
*υ*_as_(P–O–C)	1117	1045	—	983	1015 [51], 1031 [51], 1000–1050 [52]
*υ*_s_(P–O–C)	854	745, 786	842	762, 795	—
*υ*(P–O)	990	950	928, 972	—	—
*υ*(P–F)	873	843	—	—	838 [51]
*δ*(P–O–C)	594	521	473	461	778 [51]

**Figure 3.**
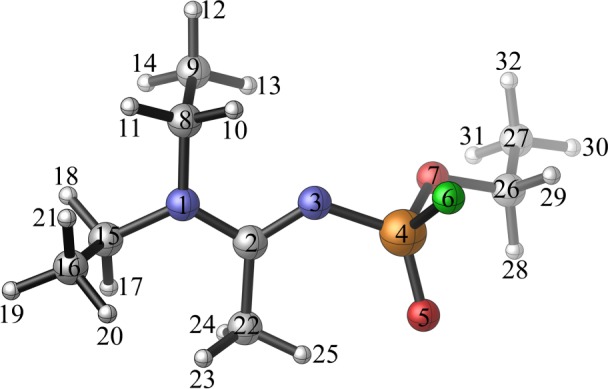
Atom labelling of A234.

The ^1^H, ^13^C, ^15^N, ^31^P and ^19^F NMR chemical shifts of A234, GB, VR and VX in water are provided in electronic supplementary material, table S3 and the atom labelling of A234 are given in figure 3.

#### 3.2.1. ^13^C NMR

The tertiary carbon atom (C2) of the acetoamidine unit of A234 resonates in a weak field with the highest chemical shift of 190.71 ppm compared to the other carbon atoms present in either A234 or GB, VR and VX. This is characteristic of amidine/guanidine-containing molecules [57,58]. The C2 atom is in an electron-poor environment, being bonded to two electronegative nitrogen atoms, and hence, less shielded as opposed to the other carbon atoms. Further, the carbon atom (C26) bonded to oxygen in A234 resonates at a higher chemical shift of 65.45 ppm while those bonded to the less electronegative nitrogen resonate at 48.30 ppm (C8) and 47.28 ppm (C15). The shielded carbon atoms (C9, C16, C22 and C27) have upfield chemical shifts within 12.43–20.94 ppm.

#### 3.2.2. ^1^H NMR

Hydrogen atoms (e.g. H10 and H11 of A234) associated with the electronegative N1 atom have downfield chemical shifts (*δ*_H10_ = 4.04 ppm and *δ*_H11_ = 2.85 ppm). Hydrogen atoms (H28 and H29) associated with the more electronegative O7 atom have slightly larger chemical shift (*δ*_H28_ = 4.14 ppm and *δ*_H29_ = 3.92 ppm). The presence of weak hydrogen bonding in A234 (figure 3; C22–H25•••O5; 2.198 Å) is also evident from the chemical shift of the H25 atom (3.53 ppm). The other two hydrogen atoms (H23 and H24) of the methyl group are at upfield chemical shifts (*δ*_H23_ = 1.87 ppm and *δ*_H24_ = 1.82 ppm).

#### 3.2.3. ^31^P NMR

The ^31^P NMR spectroscopy is particularly useful in distinguishing between the nerve agents. The chemical shift of A234 (3.89 ppm) is upfield with respect to that of GB (39.94 ppm), VR (63.45 ppm) and VX (62.72 ppm). The chemical shifts (referenced to 85% H_3_PO_4_ in C_6_D_6_ solution) of *N*^1^-phosphoryl-*N*^1^,*N*^2^-dimethylamidines of the type (EtO)_2_P(O)N(Me)C(R)=NMe (where R = Ph, CHCl_2_) were reported within −1.4 to −4.4 ppm [59], close to that of A234. In this context, it is worth highlighting that the chemical shifts are dependent on the solvent used. For instance, the chemical shift of VX lies between 60 and 63 ppm in water compared to 57.0 ppm in CDCl_3_ [60].

#### 3.2.4. ^15^N NMR

The N1 atom (−295.91 ppm) of the >N1–CR=N3– unit of A234 has an upfield chemical shift in contrast to N3 (−260.40 ppm). This compares well with the chemical shifts of the N^1^ atom of amidines of the type R^4^N^2^=CR^1^–N^1^R^2^R^3^ (where R^1^–R^4^ are alkyl groups) which lie from −269.2 to −310.5 ppm (converted to the MeNO_2_ scale) [61]. The chemical shift of the tertiary amino N1 atom of A234 is downfield compared to that of VR (−396.54 ppm) and VX (−394.96 ppm).

#### 3.2.5. ^19^F NMR

The chemical shift of the F6 atom of A234 appears at −86.22 ppm while that of GB is at −63.69 ppm.

### 3.3. Conceptual DFT-based reactivity descriptors

**Figure 4.**
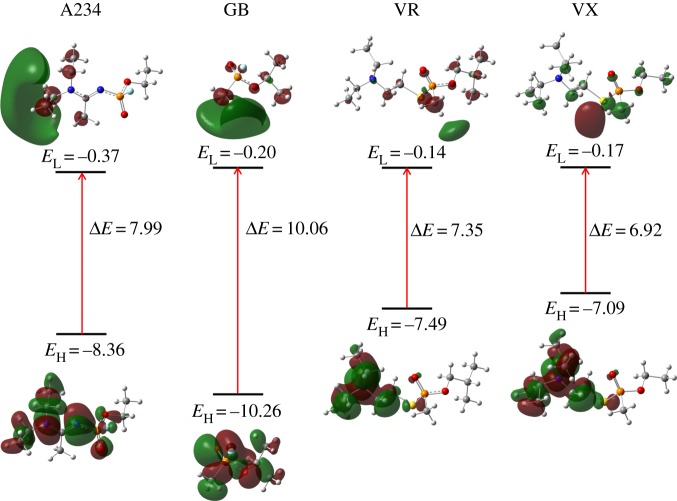
HOMO and LUMO plots (eV) of the nerve agents.

The HOMO and LUMO plots of A234, GB, VR and VX obtained using the M06-2X/6-311++G(d,p) method are illustrated in figure 4. The HOMO of A234 is mainly localized on the N atoms and the ethyl groups of the acetoamidine unit. A small contribution from the orbitals of the electronegative O and F atoms is also observed in HOMO. Its LUMO is mainly centred on the alkyl groups of the acetoamidine unit. Some similarities are also reflected from the HOMO and LUMO of GB, VR and VX. In GB, the HOMO is mainly localized on its alkyl groups as well as its O atom, while in VR/VX, the HOMO is centred on their tertiary amino unit. Further, LUMO is mainly localized on the alkyl groups of GB, VR and VX. A close analysis of the MO coefficients indicates that the orbitals of the P atom contribute to some extent to the HOMO and LUMO of A234, GB, VR and VX. Population analysis shows that the active orbitals associated with acetoamidine unit dominate over that of the fluorophosphate unit within the low-lying HOMOs and higher-lying LUMOs (electronic supplementary material, table S4 and figure S3).

DFT-based reactivity descriptors derived from the HOMO and LUMO energies of the nerve agents are gathered in table 2. The HOMO–LUMO energy gap (Δ*E*) or global hardness (*η*) indicates the overall stability of the molecules. The energy values (Δ*E* or *η*) increase in the order of VX (6.92 eV) < VR (7.35 eV) < A234 (7.99 eV) < GB (10.06 eV). Further, table 2 indicates that both global softness and polarizability increase in the order of GB < A234 < VR ≈ VX. Thus, VR and VX are more soft, polarizable and reactive than GB and A234.

**Table 2.** Conceptual DFT-based reactivity descriptors for the nerve agents.

parameters	A234	GB	VR	VX	derivation
dipole moment (*D*)	5.72	3.11	1.60	1.24	
polarizability (*α*) (Å^3^)	20.52	10.80	28.21	27.94	*α* = (*α*_xx_ + *α*_yy_ + *α*_zz_)/3 [62]
ionization potential (*I*) (eV)	8.36	10.26	7.49	7.09	*I* = –*E*_HOMO_ [63]
electron affinity (*A*) (eV)	0.37	0.20	0.14	0.17	*A* = –*E*_LUMO_ [63]
chemical potential (*μ*) (eV)	−4.36	−5.23	−3.82	−3.63	*μ* = –(*I* + *A*)/2 [64]
electronegativity (*χ*) (eV)	4.36	5.23	3.82	3.63	*χ* = –*μ* [65,66]
global hardness (*η*) (eV)	7.99	10.06	7.35	6.92	*η* = *I*−*A* [64,67–69]
global softness (*S*) (eV^−1^)	0.13	0.10	0.14	0.14	*S* = 1/*η* [62]
electrophilicity index (*ω*) (eV)	1.19	1.36	0.99	0.95	*ω* = *μ*^2^/2*η* [70–73]

A large HOMO–LUMO energy gap has been associated with a large dipole moment [74]. VX has both the lowest energy gap and the lowest dipole moment. A234 has the largest dipole moment (which points towards the N3–P4 bond) and hence, is more polar. The dipole moment decreases on going from A234 → GB → VR → VX.

The electrophilicity index increases from VX → VR → A234 → GB. All the nerve agents are highly electrophilic. They are prone to accept electron density from incoming nucleophiles, bearing oxygen, nitrogen and sulfur donor atoms, preferentially at the electropositive centre/s of the nerve agents. NBO analysis of A234 indicates that the carbon atom (0.578e) of the >N1–CR=N3– acetoamidine unit and the phosphorus atom (2.498e) are both positively charged. All other carbon atoms are negatively charged within a range of −0.033e to −0.598e (see electronic supplementary material, table S5). Thus, these two particular centres (C2 and P4) will be the prime target of nucleophiles. By contrast, GB, VR and VX have only one electropositive centre where nucleophilic attack can take place. Their phosphorus centres (P_GB_ = 2.365e, P_VR_ = 1.974e, P_VX_ = 1.971e) are less positively charged than that of A234. This suggests that a nucleophile may attack the phosphorus centre of A234 more readily than that of GB, VR and VX.

### 3.4. Molecular electrostatic potential

**Figure 5.**
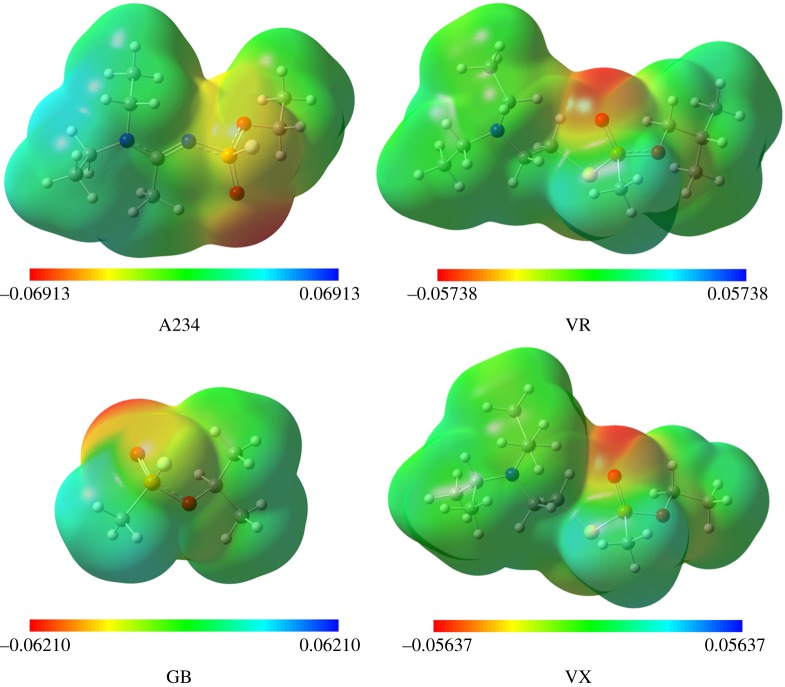
MEP surfaces (arb. units) of the nerve agents.

The MEP surfaces of A234, GB, VR and VX, obtained using the M06-2X/6-311++G(d,p) method, are shown in figure 5 and their back surfaces are also provided in electronic supplementary material, figure S4. Some common features are observed in the MEP surfaces of the four nerve agents. The negative charge (red region) is mainly localized on the oxygen atom of the P=O unit and this will favour strong hydrogen bonding, for example, with the amino acid residue of the AChE active site. The regions governing the electron-rich O (coordinated to alkyl group), S and F atoms are weakly negative (yellow in colour) and these may form weak hydrogen bonding with the –NH_2_ moiety of the amino acid of AChE. The region at the nitrogen atom of the tertiary amino unit of VR and VX are also weakly negative (see electronic supplementary material, figure S4); however, the same observation is not reciprocated by A234. The surfaces around the nitrogen atom (N1) together with the coordinated ethyl groups of A234 are weakly positive (pale blue in colour). The –NEt_2_ unit is electron-deficient most probably due to the significant charge transfer from the lone pair of N1 atom to the anti-bonding orbital of C2=N3 bond (as observed from the second-order perturbation theory analysis within the NBO framework). The observation made from the MEP analysis also correlates with the natural charges of the N atoms of the nerve agents. The N1 atom of A234 (−0.493e) is less negatively charged than that of VR (−0.597e) and VX (−0.598e). On the other hand, the N3 atom of A234 is more negative (−1.005e). Electrophiles will approach the N3 centre more readily than the N1 centre.

### 3.5. ADME parameters

The ADME parameters, namely lipophilicity (log *P*), solubility (log *S*), topological polar surface area (TPSA) and skin permeability (log *K*_p_) of the nerve agents, are summarized in table 3. The nerve agents are lipophilic in nature (the log *P*_o/w_ values are positive). The lipophilicity generally increases in the order of VX ≈ VR > A234 > GB. Data derived from SwissADME indicate a good correlation among the molecular weight, TPSA, lipophilicity, solubility, human gastrointestinal absorption, blood–brain barrier permeability and skin permeability of the nerve agents A234, GB, VR and VX (see electronic supplementary material, figures S5a–d). Lipophilicity increases as the molecular weight and TPSA increase. This can be correlated with an increase in the penetration of the nerve agent in the CNS [76]. All the nerve agents are soluble in water and solubility is in the order of GB > A234 > VR ≈ VX. It is known that the solubility of the compound can influence the absorption and blood–brain barrier permeability [77]. The solubility of the nerve agents can be associated with their high human gastrointestinal absorption as well as good blood–brain barrier and skin permeability. SwissADME predicts that the skin permeability of A234 and GB are comparable and that their more negative log *K*_p_ values indicate that they are marginally less skin permeant than VR and VX [44].

**Table 3.** ADME properties of the nerve agents.

	log *P*				log *S*^b^			TPSA^b^ (Å^2^)	log [*K*_p_/(cm s^−1^)]^b^
	computed^a^	consensus^b^	*A*log*P*^c^	lit.^d^	ESOL	Ali	silicos-IT		
A234	1.33	1.87	1.64	—	−1.49	−1.72	−2.12	51.71	−6.93
GB	0.75	1.08	0.54	0.30	−0.77	−0.62	−1.10	36.11	−6.94
VR	2.12	2.47	2.15	—	−2.29	−3.08	−2.72	64.65	−6.45
VX	1.16	2.55	2.41	2.09	−2.28	−3.18	−3.10	64.65	−6.38

^a^log *P* obtained using the M06-2X/6-311++G(d,p) method.

^b^Consensus log *P*, log *S*, TPSA and log *K*_p_ obtained from SwissADME [44].

^c^log *P* obtained from VCCLab [45].

^d^Literature values [75].

### 3.6. Nerve agent poisoning and antidotes

**Scheme 2.**
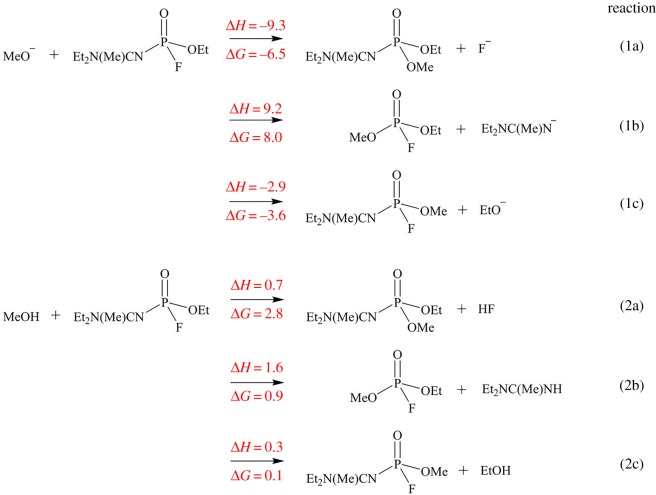
Phosphonylation reaction between A234 and AChE models. The enthalpy (Δ*H*) and free energy change (Δ*G*) are in kcal mol^−1^.

Nerve agent poisoning is caused from the inhibition of the AChE enzyme activity; thereby, forming a covalent bond between the phosphorus atom of the nerve agent and the alcoholic oxygen of the serine residue of the active site. Modelling the reaction between AChE and nerve agents is complex and time-consuming due to the large size of the protein and the requirement of high-accuracy computations. This reaction can alternatively be studied with model species. The simplest model for the active serine site of AChE is methanol [78]. Thus, the phosphonylation reactions of A234 with both the deprotonated CH_3_O^–^ anion and the neutral CH_3_OH molecule were investigated using the M06-2X/6-311++G(d,p) method (scheme 2). The corresponding reactions for GB, VR and VX are provided in electronic supplementary material, scheme S1. The central phosphorus atom of A234 is attached to three potential leaving groups –F, –OEt or –N=C(Me)NEt_2_. Thus, the reaction for the displacement of each leaving group via nucleophilic attack by CH_3_O^–^/CH_3_OH was studied. The enthalpy and free energy change of the reactions indicate that attack by the CH_3_O^–^ anion is favoured over CH_3_OH. This is also observed in the case of GB, VR and VX, except for the processes involving the cleavage of the P–S bond. Further, attack by the CH_3_O^–^ anion will result in the preferential displacement of the –F group over that of –OEt or –N=C(Me)NEt_2_. The relative energies for the substitution of –F by –OMe are comparable for A234 (Δ*H* = −9.3 kcal mol^−1^ and Δ*G* = −6.5 kcal mol^−1^) and GB (Δ*H* = −9.3 kcal mol^−1^ and Δ*G* = −7.5 kcal mol^−1^).

**Scheme 3.**
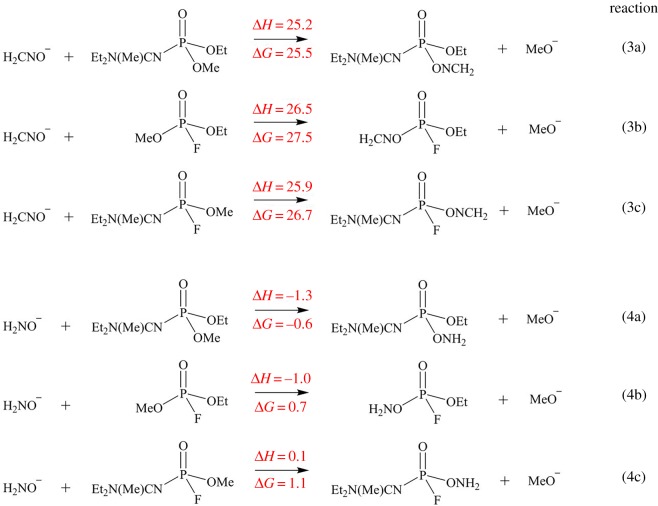
Reactivation of A234-inhibited AChE model induced by formoximate and hydroxylamine anions. The enthalpy (Δ*H*) and free energy change (Δ*G*) are in kcal mol^−1^.

Nerve agent poisoning can traditionally be treated with pralidoxime in conjunction with atropine. The main function of the oxime is to act as a reactivator of the phosphorylated AChE [79]. Thus, the effect of two antidotes on the reactivation of the A234-inhibited AChE model was probed using the M06-2X/6-311++G(d,p) method (scheme 3). The first model antidote used corresponds to the simplest oximate, formoximate anion (H_2_C=NO^−^). The second one is the hydroxylamine (H_2_NO^−^) anion which has been predicted to be a better antidote than formoximate for the reactivation process of GB-inhibited AChE [80]. The current study also indicates that reactivation of A234-inhibited AChE model will preferentially be induced by hydroxylamine than by the formoximate anion. Reaction with the formoximate anion is highly endergonic for all studied nerve agents (scheme 3; electronic supplementary material, scheme S2). Thus, this suggests that the A234-inhibited AChE model obtained from the displacement of the –F atom can be successfully reactivated using the hydroxylamine anion.

### 3.7. Hydrolysis

**Scheme 4.**
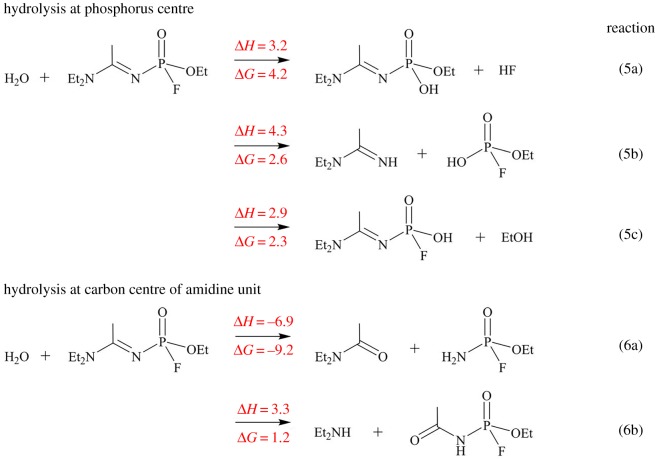
Hydrolysis of A234. The enthalpy (Δ*H*) and free energy change (Δ*G*) are in kcal mol^−1^.

Nerve agents commonly undergo nucleophilic attack by the water molecule at the electropositive phosphorus centre via either an S_N_1 or S_N_2 hydrolysis reaction [81]. Thus, hydrolysis reaction at the P4 centre of A234 was investigated using the M06-2X/6-311++G(d,p) method. This is illustrated by reactions 5a–c (scheme 4) which involve the displacement of the leaving groups –F, –OEt and –N=C(Me)NEt_2_, respectively. The free energy values indicate that these processes are endergonic. The carbon atom (C2) of the acetoamidine unit of A234 is also electropositive. Hydrolysis at the acetoamidine unit can take place via two different pathways as highlighted by Wu *et al*. [82]. Nucleophilic attack by the water molecule at the C2 centre is illustrated by reactions 6a,b (scheme 4) which involve bond breaking at N3=C2 and C2–N1, respectively. Reaction 6a is exergonic (Δ*G* = −9.2 kcal mol^−1^). Based on the reaction energetics and in the absence of information on A234, it can be deduced that its hydrolysis may potentially take place via reaction 6a to yield *N*,*N*-diethylacetamide and ethyl phosphoramidofluoridate.

### 3.8. Heat of formation and bond dissociation energy

The $\Delta_{f}H_{298}^{\circ }$ was calculated using the CBS-QB3 method for the nerve agents. The formation of the nerve agents is exothermic with $\Delta_{f}H_{298}^{\circ }$ of −238.13 (A234), −240.49 (GB), −179.54 (VR) and −179.51 (VX) kcal mol^−1^. The $\Delta_{f}H_{298}^{\circ }$ values were estimated as in [48] and details are provided in the electronic supplementary material. GB is marginally more stable than A234. GB and A234 have greater thermal stability than VR and VX (≈ 60 kcal mol^−1^).

**Figure 6.**
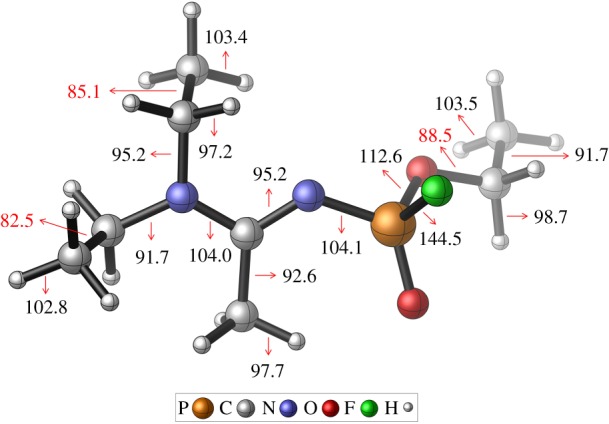
BDEs (kcal mol^−1^) of A234 obtained using the CBS-QB3 method.

Figure 6 illustrates the $\Delta_{r}H_{298}^{\circ }$ values for the dissociation of various bonds in A234 and these were compared with those of GA and GB [48]. The $\Delta_{r}H_{298}^{\circ }$ for the dissociation of the P–F bond to form the radicals •F and •P=O(OEt)(N=C(CH_3_)(NEt_2_)) was calculated to be 144.5 kcal mol^−1^. The P–F bond is the strongest and this is comparable to that of GB (143.2 kcal mol^−1^) [48]. The C–C bond breakings of the acetoamidine unit correlate to the lowest BDEs (82.5 and 85.1 kcal mol^−1^) and these dissociations will predominate in unimolecular initiation reactions. These C–C BDEs are significantly lower than that of the –OEt unit of A234 (91.7 kcal mol^−1^) and GA (93.8 kcal mol^−1^) [48]. Significant amount of electron density transfer from the lone pair of N1 atom to the anti-bonding orbital of the C2=N3 bond has a destabilizing effect on the ethyl groups attached to the N1 atom. There is also a slight difference between the C–C bond distances of the acetoamidine (C8–C9 = 1.530 Å and C15–C16 = 1.532 Å) and –OEt (C26–C27 = 1.515 Å) units of the B3LYP/6-311G(2d,d,p) optimized structure of A234. The next favourable unimolecular initiation will occur at the O–C bond of A234.

## 4. Summary

Theoretical methods were employed to study (i) the molecular, spectroscopic, electronic and toxicity properties, (ii) poisoning action and antidotes based on model reactions, and (iii) hydrolysis and thermal degradation of A234. Some of these parameters were compared with GB, VR and VX. Distinct features in the spectra of A234 are observed, namely (i) a sharp peak due to C=N stretching at 1670 cm^–1^ in the IR spectra, (ii) a large ^13^C NMR chemical shift of 190.71 ppm due to the –N=CR–N< acetoamidine carbon atom, and (iii) a relatively small ^31^P NMR chemical shift of 3.89 ppm. NBO analysis indicates that A234 may have diverse chemistry to other nerve agents due to the presence of two active electronegative centres, namely the carbon atom of the –N=CR–N< acetoamidine group and the phosphorus atom. The energetics for (i) the reactions of the nerve agents with AChE model nucleophiles, (ii) the reactions of nerve agents-inhibited AChE with model antidotes, and (iii) hydrolysis and thermal degradation of A234 will serve as foundations for future computations. Detailed mechanistic studies are currently being carried out in our laboratory for an in-depth understanding of the hydrolysis of A234. Overall, this study suggests that VX and VR are potentially more reactive than A234 and GB further to (i) their lowest HOMO–LUMO energy gaps, (ii) marginally high skin permeability, and (iii) highly negative Δ*G* values associated with their reaction with MeO^−^ and MeOH (for the cleavage of the P–S bond). The current theoretical work could not authenticate the claim made by Mirzayanov on A234 being more potent than VX and this is in accordance with the recent study carried out by Carlsen [21]. The findings from this research work should provide incentives towards efficient detection, development of antidotes and destruction of A234.

## Supplementary Material

Figures, Tables and Schemes

## Footnotes

1 This work used the Extreme Science and Engineering Discovery Environment (XSEDE), which is supported by National Science Foundation grant number OCI-1053575.

## Data accessibility

Cartesian coordinates for the optimized geometries are available as electronic supplementary material.
