# Supplementary material for: Theoretical study of the molecular aspect of the suspected novichok agent A234 of the Skripal poisoning
Source: R Soc Open Sci. 2019 Feb 6;6(2):181831. doi: 10.1098/rsos.181831 (PMC6408395; doi:10.1098/rsos.181831)
Supplement: Figures, Tables and Schemes [file rsos181831supp1.docx]

**Electronic Supplementary Material**

**Theoretical study of the molecular aspect of the suspected novichok agent A234 of the Skripal poisoning**

Hanusha Bhakhoa ^a^, Lydia Rhyman ^a,b^ and Ponnadurai Ramasami* ^a,b^

^a^ Computational Chemistry Group, Department of Chemistry, Faculty of Science, University of Mauritius, Réduit 80837, Mauritius

^b^ Department of Applied Chemistry, University of Johannesburg, Doornfontein, Johannesburg 2028, South Africa

*Corresponding author e-mail: p.ramasami@uom.ac.mu (P.R.)

| **Table of contents** | **Page numbers** |
| --- | --- |
|  |  |
| Conformational analysis | S3, S4 |
| Geometrical parameters and rotational constants | S5-S7 |
| NMR spectroscopy | S8-S9 |
| Population and NBO analyses | S10-S12 |
| Molecular electrostatic potential | S13 |
| ADME parameters | S14-S17 |
| Nerve agent poisoning and antidotes | S18-S23 |
| Heat of formation | S24 |
| Cartesian coordinates | S25-S93 |
| References | S94 |
| Further reading | S94 |

**Conformational analysis**





Figure S1: Gas-phase optimised geometries and relative energies of A234 obtained using the B3LYP/6-311++G(d,p) method.





Figure S1: (*Continued*) Gas-phase optimised geometries and relative energies of A234 obtained using the B3LYP/6-311++G(d,p) method.

**Geometrical parameters**

Table S1: Selected structural parameters of A234 calculated using the M06-2X/6-311++G(d,p), B3LYP/6-311++G(d,p) and MP2/6-311++G(d,p) methods. The associated atom numbers are depicted in Figure S2.

|  | M06-2X | B3LYP | MP2 |
| --- | --- | --- | --- |
| Bond angles (°) |  |  |  |
| N1−C2−N3 | 117.4 | 117.4 | 117.4 |
| C2−N3−P4 | 128.4 | 129.2 | 123.3 |
| P4−O7−C26 | 118.4 | 120.0 | 118.3 |
| O7−C26−C27 | 107.5 | 107.8 | 107.2 |
| O5−P4−F6 | 110.1 | 109.9 | 110.9 |
| O5−P4−O7 | 114.4 | 114.9 | 114.8 |
| O5−P4−N3 | 123.3 | 123.5 | 122.3 |
| Dihedral angles (°) |  |  |  |
| N1−C2−N3−P4 | -179.4 | 178.0 | 179.9 |
| C2−N3−P4−O7 | 123.1 | 132.2 | 75.9 |
| C2−N3−P4−F6 | -132.2 | -123.2 | -178.9 |
| C2−N3−P4−O5 | -8.1 | 0.7 | -55.5 |
| N3−P4−O7−C26 | -177.5 | -176.4 | -179.9 |
| P4−O7−C26−C27 | 171.7 | 168.8 | 176.5 |
| P4−N3−C2−C22 | -0.2 | -2.9 | -1.2 |

| A234 | 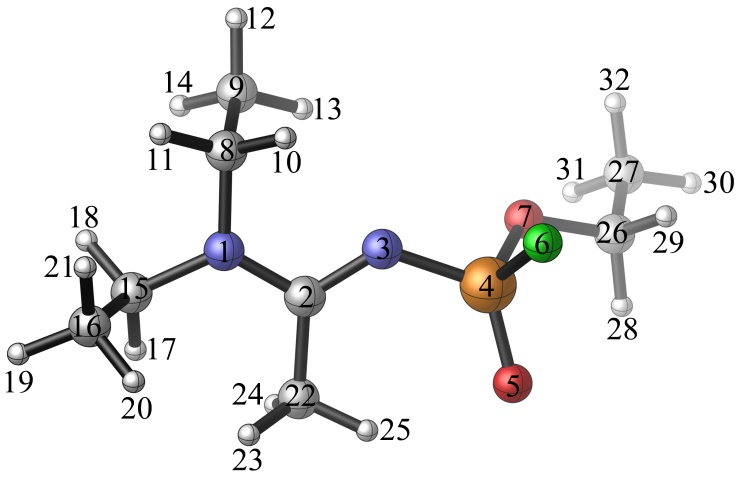 | 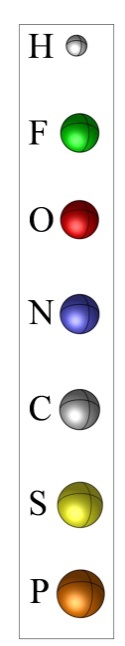 |
| --- | --- | --- |
| GB | 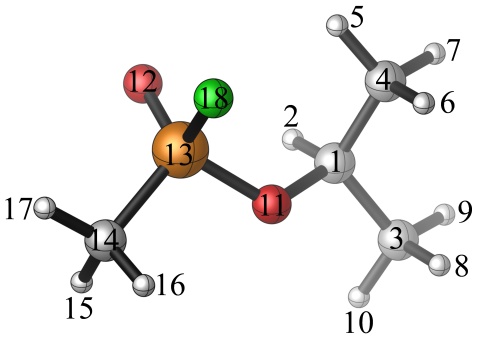 |  |
| VR | 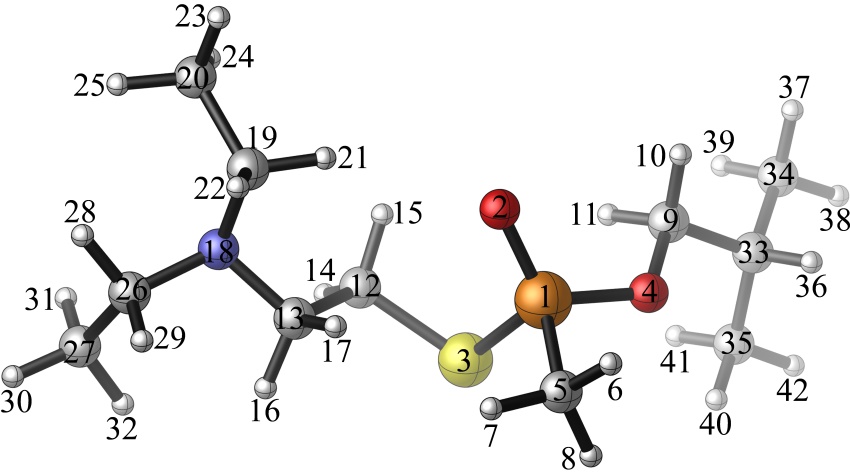 |  |
| VX | 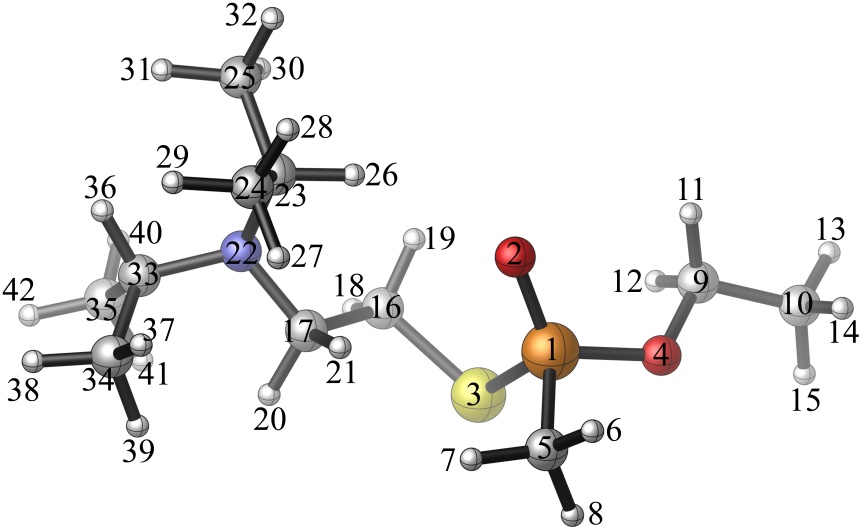 |  |

Figure S2: Gas-phase optimised geometries of the nerve agents (with atom labeling) obtained using the M06-2X/6-311++G(d,p) method.

Table S2: Rotational constants (MHz) of the nerve agents.

|  | A | B | C |
| --- | --- | --- | --- |
|  | M06-2X/6-311++G(d,p) | | |
| A234 | 1051.6814 | 316.2697 | 282.4393 |
| GB | 2860.7263 | 1174.1691 | 1063.4548 |
| VR | 785.3395 | 173.2870 | 169.7328 |
| VX | 766.4814 | 198.4742 | 190.4707 |
|  | B3LYP/6-311++G(d,p) | | |
| A234 | 1032.8919 | 312.1392 | 274.9336 |
| GB | 2776.9919 | 1127.3768 | 1022.6174 |
| VR | 805.5108 | 154.5757 | 150.9771 |
| VX | 767.9495 | 180.5702 | 173.9372 |
|  | MP2/6-311++G(d,p) | | |
| A234 | 967.8993 | 316.7667 | 306.7438 |
| GB | 2821.9292 | 1156.6114 | 1048.0549 |
| VR | 805.1473 | 170.5594 | 166.5203 |
| VX | 777.8018 | 199.0022 | 190.6705 |

The rotational constants of A234, GB, VR, and VX were obtained using the M06-2X/6-311++G(d,p), B3LYP/6-311++G(d,p) and MP2/6-311++G(d,p) methods (Table S2). The values predicted using the different methods are satisfactorily comparable to each other. The rotational constants of GB, in particular, the ones predicted from the M06-2X/6-311++G(d,p) method, are closer to those obtained experimentally where A = 2874.0710(9) MHz, B = 1168.5776(4) MHz, and C = 1056.3363(4) MHz.^S1^ The experimental results suggest that the M06-2X functional outperforms the B3LYP functional and the MP2 method.

**NMR spectroscopy**

Table S3: ^1^H, ^13^C, ^15^N, ^31^P and ^19^F NMR chemical shifts (ppm) of the nerve agents obtained using the M06-2X/6-311++G(d,p) method. The associated atom numbers are clearly depicted in Figure S2.

^1^H NMR

| A234 | | GB | | VR | | VX | |
| --- | --- | --- | --- | --- | --- | --- | --- |
| H10 | 4.04 | H2 | 4.65 | H6 | 1.76 | H6 | 1.74 |
| H11 | 2.85 | H5 | 1.65 | H7 | 1.53 | H7 | 1.57 |
| H12 | 0.87 | H6 | 1.36 | H8 | 1.64 | H8 | 1.63 |
| H13 | 1.66 | H7 | 1.26 | H10 | 3.89 | H11 | 3.94 |
| H14 | 0.99 | H8 | 1.37 | H11 | 3.63 | H12 | 4.03 |
| H17 | 3.50 | H9 | 1.22 | H14 | 2.55 | H13 | 1.27 |
| H18 | 3.08 | H10 | 1.51 | H15 | 3.03 | H14 | 1.43 |
| H19 | 1.04 | H15 | 1.43 | H16 | 2.68 | H15 | 1.36 |
| H20 | 1.09 | H16 | 1.53 | H17 | 1.76 | H18 | 2.46 |
| H21 | 1.34 | H17 | 1.45 | H21 | 2.81 | H19 | 2.95 |
| H23 | 1.87 |  |  | H22 | 1.98 | H20 | 2.67 |
| H24 | 1.82 |  |  | H23 | 0.97 | H21 | 2.11 |
| H25 | 3.53 |  |  | H24 | 0.90 | H26 | 3.23 |
| H28 | 4.14 |  |  | H25 | 1.20 | H27 | 1.39 |
| H29 | 3.92 |  |  | H28 | 2.52 | H28 | 0.76 |
| H30 | 1.26 |  |  | H29 | 1.90 | H29 | 0.84 |
| H31 | 1.37 |  |  | H30 | 1.03 | H30 | 0.94 |
| H32 | 1.46 |  |  | H31 | 0.89 | H31 | 1.29 |
|  |  |  |  | H32 | 1.24 | H32 | 0.98 |
|  |  |  |  | H36 | 1.82 | H36 | 2.88 |
|  |  |  |  | H37 | 1.02 | H37 | 1.09 |
|  |  |  |  | H38 | 1.14 | H38 | 0.86 |
|  |  |  |  | H39 | 0.63 | H39 | 0.92 |
|  |  |  |  | H40 | 1.39 | H40 | 0.93 |
|  |  |  |  | H41 | 0.58 | H41 | 1.09 |
|  |  |  |  | H42 | 1.02 | H42 | 0.89 |
|  |  |  |  |  |  |  |  |

Table S3: (*Continued*) ^1^H, ^13^C, ^15^N, ^31^P and ^19^F NMR chemical shifts (ppm) of the nerve agents obtained using the M06-2X/6-311++G(d,p) method. The associated atom numbers are clearly depicted in Figure S2.

^13^C NMR

| A234 | | GB | | VR | | VX | |
| --- | --- | --- | --- | --- | --- | --- | --- |
| C2 | 190.71 | C1 | 78.82 | C5 | 27.53 | C5 | 27.96 |
| C8 | 48.30 | C3 | 25.66 | C9 | 75.67 | C9 | 64.16 |
| C9 | 12.43 | C4 | 26.78 | C12 | 38.09 | C10 | 17.58 |
| C15 | 47.28 | C14 | 13.14 | C13 | 62.41 | C16 | 39.18 |
| C16 | 14.62 |  |  | C19 | 54.02 | C17 | 52.46 |
| C22 | 20.94 |  |  | C20 | 16.48 | C23 | 54.09 |
| C26 | 65.45 |  |  | C26 | 52.08 | C24 | 18.76 |
| C27 | 18.03 |  |  | C27 | 16.42 | C25 | 24.92 |
|  |  |  |  | C33 | 33.88 | C33 | 48.38 |
|  |  |  |  | C34 | 19.96 | C34 | 21.81 |
|  |  |  |  | C35 | 22.13 | C35 | 27.04 |

^15^N NMR

| A234 | | GB | | VR | | VX | |
| --- | --- | --- | --- | --- | --- | --- | --- |
| N1 | -295.91 | − | | N18 | -396.54 | N22 | -394.96 |
| N3 | -260.40 |  |  |  |  |  |  |

^31^P NMR

| A234 | | GB | | VR | | VX | |
| --- | --- | --- | --- | --- | --- | --- | --- |
| P4 | 3.89 | P13 | 39.94 | P1 | 63.45 | P1 | 62.72 |

^19^F NMR

| A234 | | GB | |  |  |  |  |
| --- | --- | --- | --- | --- | --- | --- | --- |
| F6 | -86.22 | F18 | -63.69 |  |  |  |  |

**Population and NBO analyses**

Table S4: Population analysis of the M06-2X/6-311++G(d,p) optimised structure of A234.


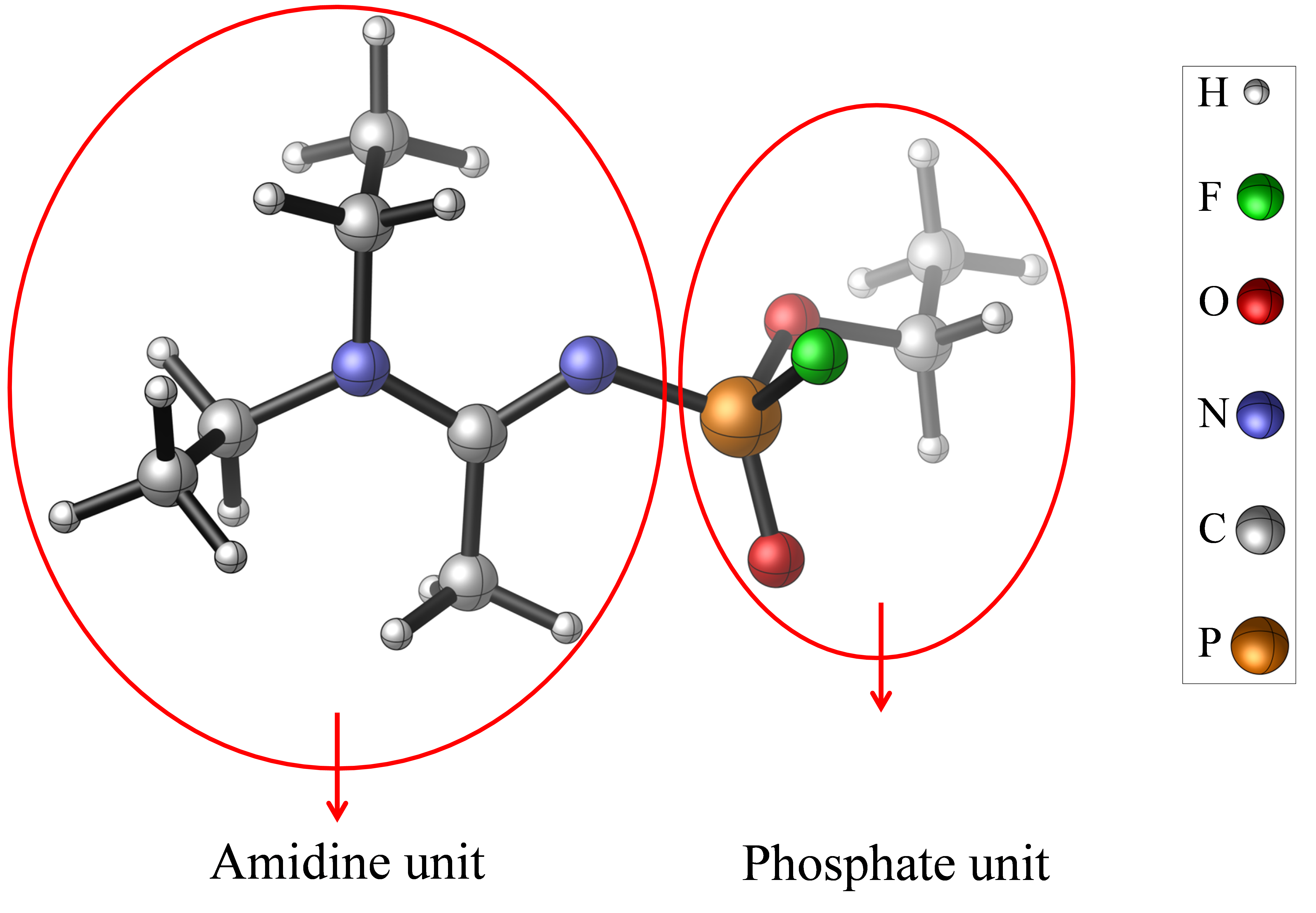


| MO number | MO | Energy (eV) | % Amidine unit | % Phosphate unit |
| --- | --- | --- | --- | --- |
| 54 | HOMO-6 | -11.117 | 69 | 31 |
| 55 | HOMO-5 | -11.067 | 37 | 63 |
| 56 | HOMO-4 | -10.579 | 41 | 59 |
| 57 | HOMO-3 | -10.285 | 59 | 41 |
| 58 | HOMO-2 | -9.769 | 19 | 81 |
| 59 | HOMO-1 | -8.860 | 84 | 16 |
| 60 | HOMO | -8.356 | 86 | 14 |
| 61 | LUMO | -0.367 | 99 | 1 |
| 62 | LUMO+1 | -0.003 | 93 | 7 |
| 63 | LUMO+2 | 0.067 | 98 | 2 |
| 64 | LUMO+3 | 0.174 | 62 | 38 |
| 65 | LUMO+4 | 0.364 | 76 | 24 |
| 66 | LUMO+5 | 0.638 | 94 | 6 |
| 67 | LUMO+6 | 0.662 | 82 | 18 |

The population analysis is a quantitative description of the molecular orbitals (MOs); it quantifies the contribution of each part of a molecule to the MOs. The percentage contribution of the acetoamidine and fluorophosphate units to some of the low-lying HOMO’s and upper-lying LUMO’s are provided in Table S4 and these were obtained from the Chemissian software.^S2^ Corresponding MO plot is also provided (see Figure S3). The values indicate that the highest contribution comes from the acetoamidine unit suggesting that the acetoamidine unit may be more reactive than the fluorophosphate unit.

**
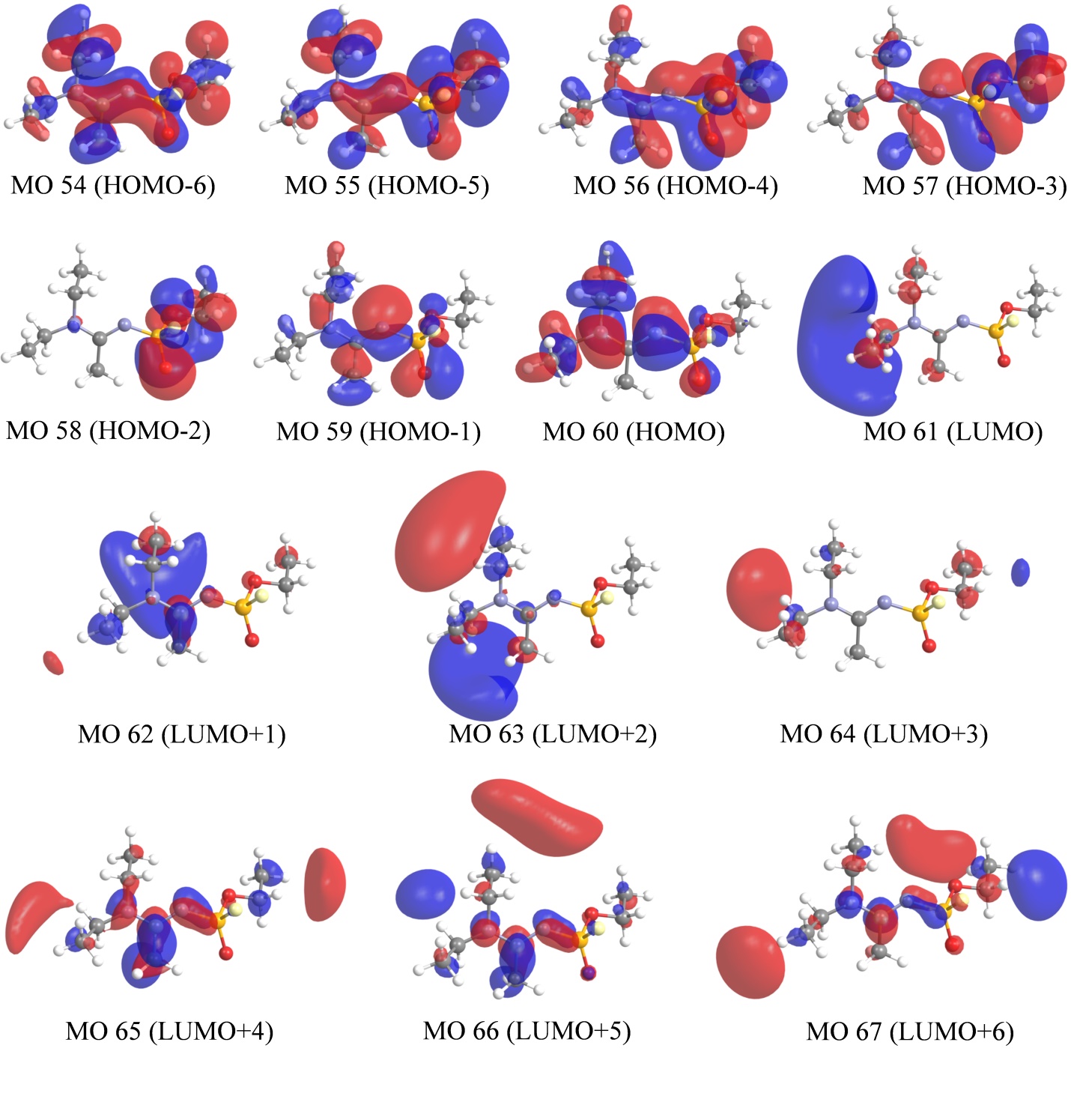
**

Figure S3: MO plots of A234 obtained using the M06-2X/6-311++G(d,p) method.

Table S5: Computed charge densities (natural charges, e) on all the centres of the nerve agents obtained using the M06-2X/6-311++G(d,p) method.^[a]^

| A234 | | GB | | VR | | | | VX | | | |
| --- | --- | --- | --- | --- | --- | --- | --- | --- | --- | --- | --- |
| N 1 | -0.493 | C 1 | 0.126 | P 1 | 1.974 | C 33 | -0.257 | P 1 | 1.971 | C 33 | -0.016 |
| C 2 | 0.578 | H 2 | 0.193 | O 2 | -1.085 | C 34 | -0.583 | O 2 | -1.088 | C 34 | -0.605 |
| N 3 | -1.005 | C 3 | -0.595 | S 3 | -0.128 | C 35 | -0.586 | S 3 | -0.130 | C 35 | -0.581 |
| P 4 | 2.498 | C 4 | -0.614 | O 4 | -0.866 | H 36 | 0.202 | O 4 | -0.862 | H 36 | 0.195 |
| O 5 | -1.096 | H 5 | 0.221 | C 5 | -0.967 | H 37 | 0.205 | C 5 | -0.968 | H 37 | 0.209 |
| F 6 | -0.573 | H 6 | 0.210 | H 6 | 0.248 | H 38 | 0.209 | H 6 | 0.247 | H 38 | 0.207 |
| O 7 | -0.851 | H 7 | 0.213 | H 7 | 0.244 | H 39 | 0.200 | H 7 | 0.243 | H 39 | 0.196 |
| C 8 | -0.177 | H 8 | 0.208 | H 8 | 0.245 | H 40 | 0.214 | H 8 | 0.246 | H 40 | 0.211 |
| C 9 | -0.597 | H 9 | 0.210 | C 9 | -0.020 | H 41 | 0.201 | C 9 | -0.033 | H 41 | 0.193 |
| H 10 | 0.231 | H 10 | 0.214 | H 10 | 0.190 | H 42 | 0.204 | C 10 | -0.598 | H 42 | 0.199 |
| H 11 | 0.190 | O 11 | -0.872 | H 11 | 0.182 |  |  | H 11 | 0.187 |  |  |
| H 12 | 0.208 | O 12 | -1.067 | C 12 | -0.499 |  |  | H 12 | 0.181 |  |  |
| H 13 | 0.227 | P 13 | 2.365 | C 13 | -0.184 |  |  | H 13 | 0.209 |  |  |
| H 14 | 0.194 | C 14 | -0.989 | H 14 | 0.223 |  |  | H 14 | 0.209 |  |  |
| C 15 | -0.172 | H 15 | 0.250 | H 15 | 0.237 |  |  | H 15 | 0.208 |  |  |
| C 16 | -0.594 | H 16 | 0.250 | H 16 | 0.199 |  |  | C 16 | -0.494 |  |  |
| H 17 | 0.207 | H 17 | 0.251 | H 17 | 0.175 |  |  | C 17 | -0.190 |  |  |
| H 18 | 0.201 | F 18 | -0.575 | N 18 | -0.597 |  |  | H 18 | 0.222 |  |  |
| H 19 | 0.210 |  |  | C 19 | -0.186 |  |  | H 19 | 0.234 |  |  |
| H 20 | 0.205 |  |  | C 20 | -0.586 |  |  | H 20 | 0.201 |  |  |
| H 21 | 0.206 |  |  | H 21 | 0.220 |  |  | H 21 | 0.180 |  |  |
| C 22 | -0.675 |  |  | H 22 | 0.166 |  |  | N 22 | -0.598 |  |  |
| H 23 | 0.217 |  |  | H 23 | 0.204 |  |  | C 23 | -0.026 |  |  |
| H 24 | 0.229 |  |  | H 24 | 0.205 |  |  | C 24 | -0.602 |  |  |
| H 25 | 0.274 |  |  | H 25 | 0.192 |  |  | C 25 | -0.583 |  |  |
| C 26 | -0.033 |  |  | C 26 | -0.170 |  |  | H 26 | 0.220 |  |  |
| C 27 | -0.598 |  |  | C 27 | -0.589 |  |  | H 27 | 0.202 |  |  |
| H 28 | 0.191 |  |  | H 28 | 0.194 |  |  | H 28 | 0.210 |  |  |
| H 29 | 0.175 |  |  | H 29 | 0.164 |  |  | H 29 | 0.192 |  |  |
| H 30 | 0.207 |  |  | H 30 | 0.203 |  |  | H 30 | 0.210 |  |  |
| H 31 | 0.207 |  |  | H 31 | 0.208 |  |  | H 31 | 0.191 |  |  |
| H 32 | 0.208 |  |  | H 32 | 0.194 |  |  | H 32 | 0.203 |  |  |

[a] Refer to Figure S2 for atom labeling.

**Molecular electrostatic potential**

The MEP surface is basically a plot of electrostatic potential which is mapped onto constant potential electron density surface. It provides a useful guide towards predicting potential sites of chemical reactivity.^S3,S4^ The MEP surfaces of GA, GB, and GD have been previously studied and this has been valuable in determining the configuration or conformation which is dependent on their toxic behaviour towards the inhibition of AChE.^S5^ The MEP surfaces of A-234, GB, VR and VX, obtained using the M06-2X/6-311++G(d,p) method, is depicted in Figure 5 (main paper). In view of the non-planarity of the structures and in order to have a full picture of the MEP surfaces, their backsides are also provided in Figure S4. For instance, the colour code of the MEP surface of A-234 is within the range of -0.06913 a.u. (deepest red) and 0.06913 a.u. (deepest blue). The red and blue regions correspond to the negative and positive potential, respectively.


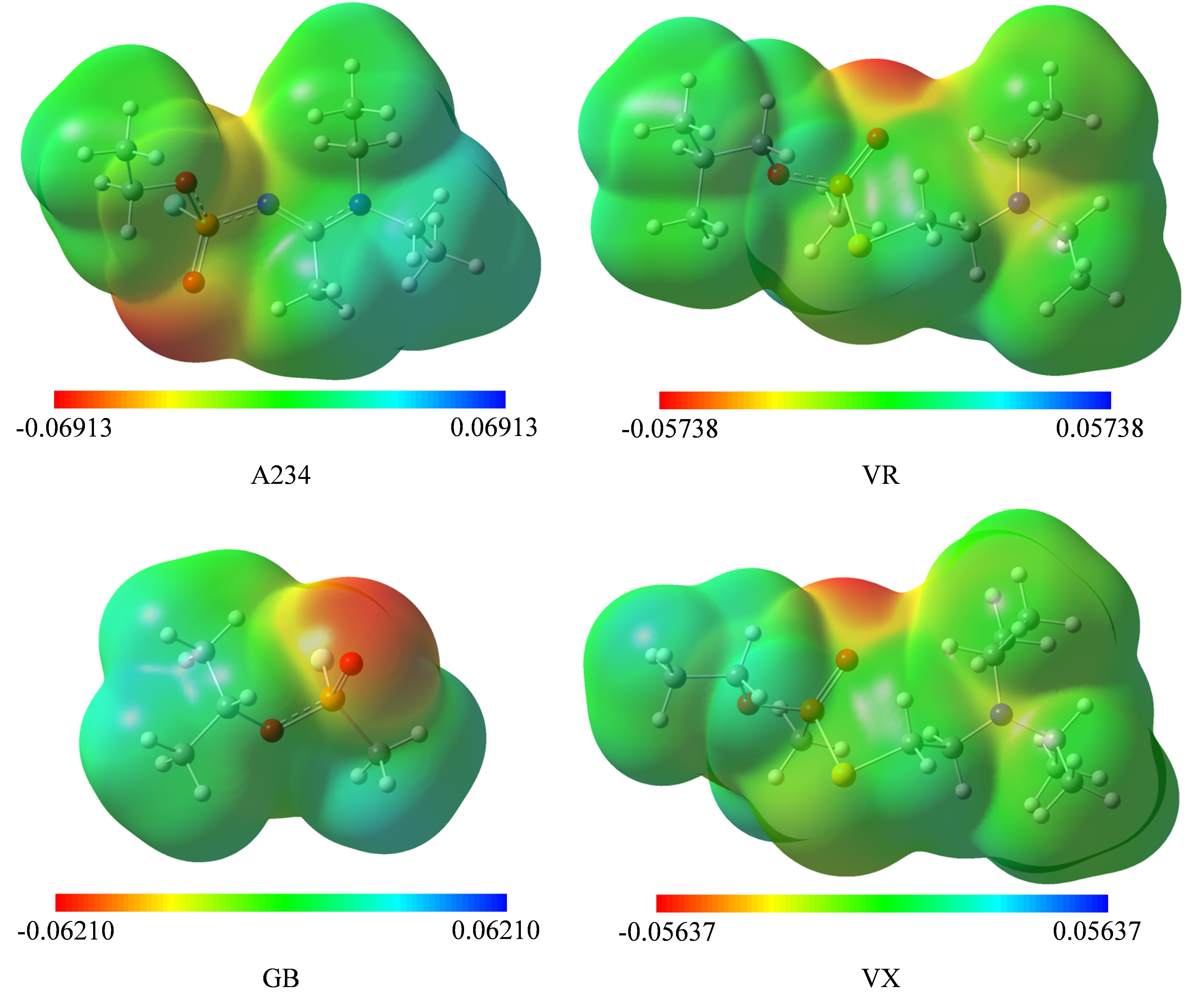


Figure S4: Backside view of the MEP surfaces (a.u.) of the nerve agents.

**ADME parameters**

Figure S5a: Pharmacokinetics, drug-likeness and medicinal chemistry friendliness of A234 evaluated using the free web tool SwissADME.


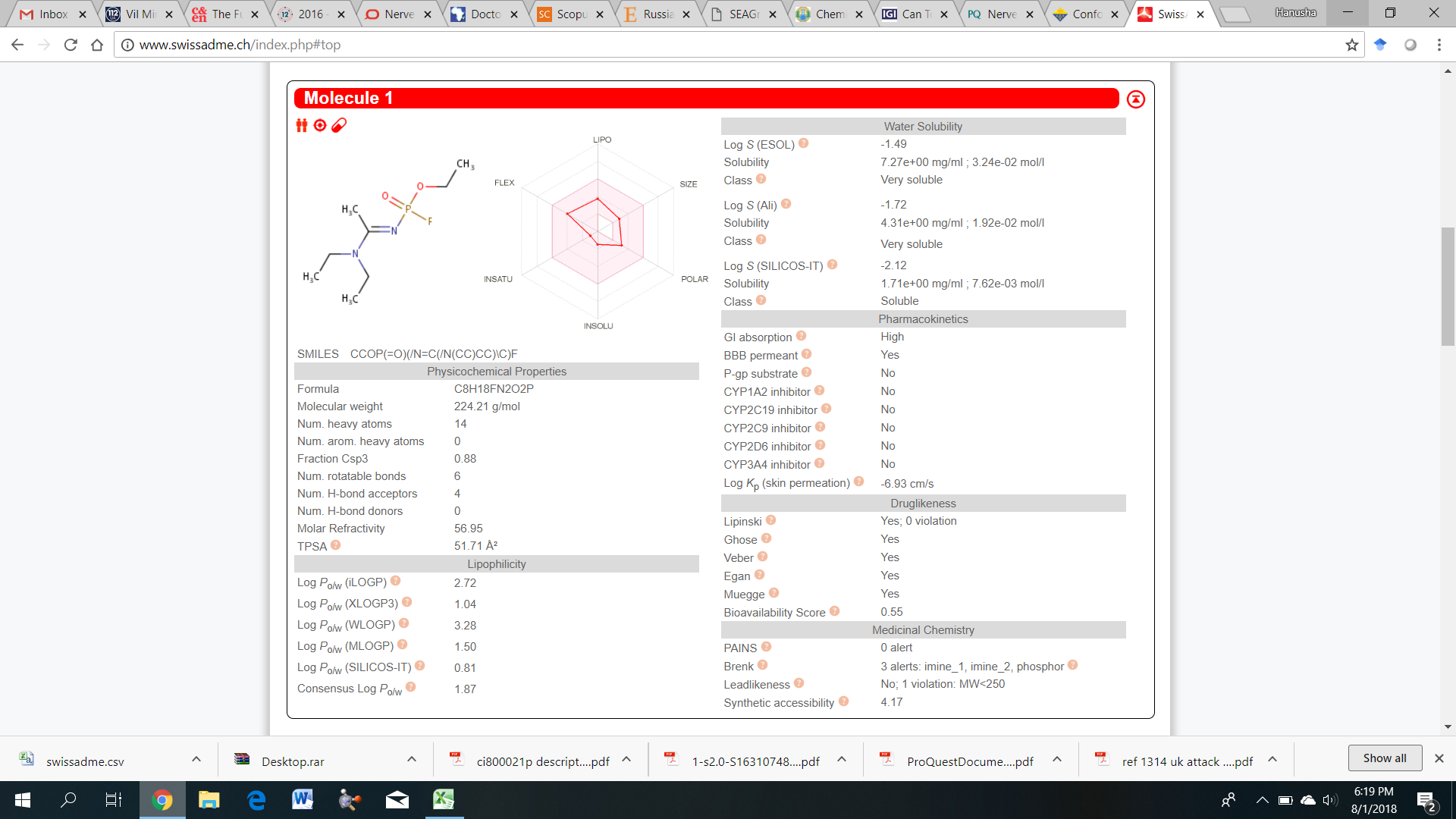


Figure S5b: Pharmacokinetics, drug-likeness and medicinal chemistry friendliness of GB evaluated using the free web tool SwissADME.


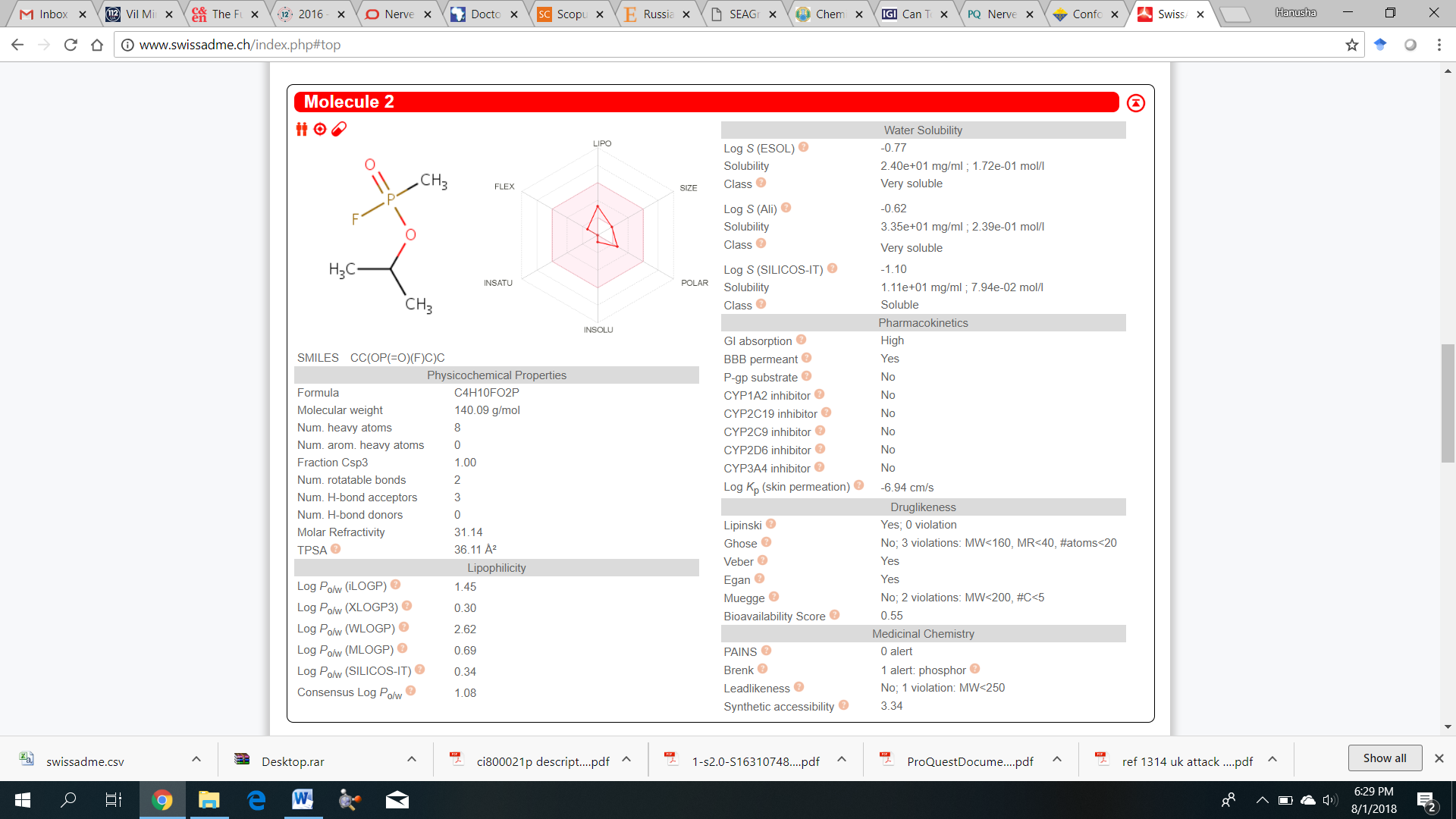


Figure S5c: Pharmacokinetics, drug-likeness and medicinal chemistry friendliness of VR evaluated using the free web tool SwissADME.


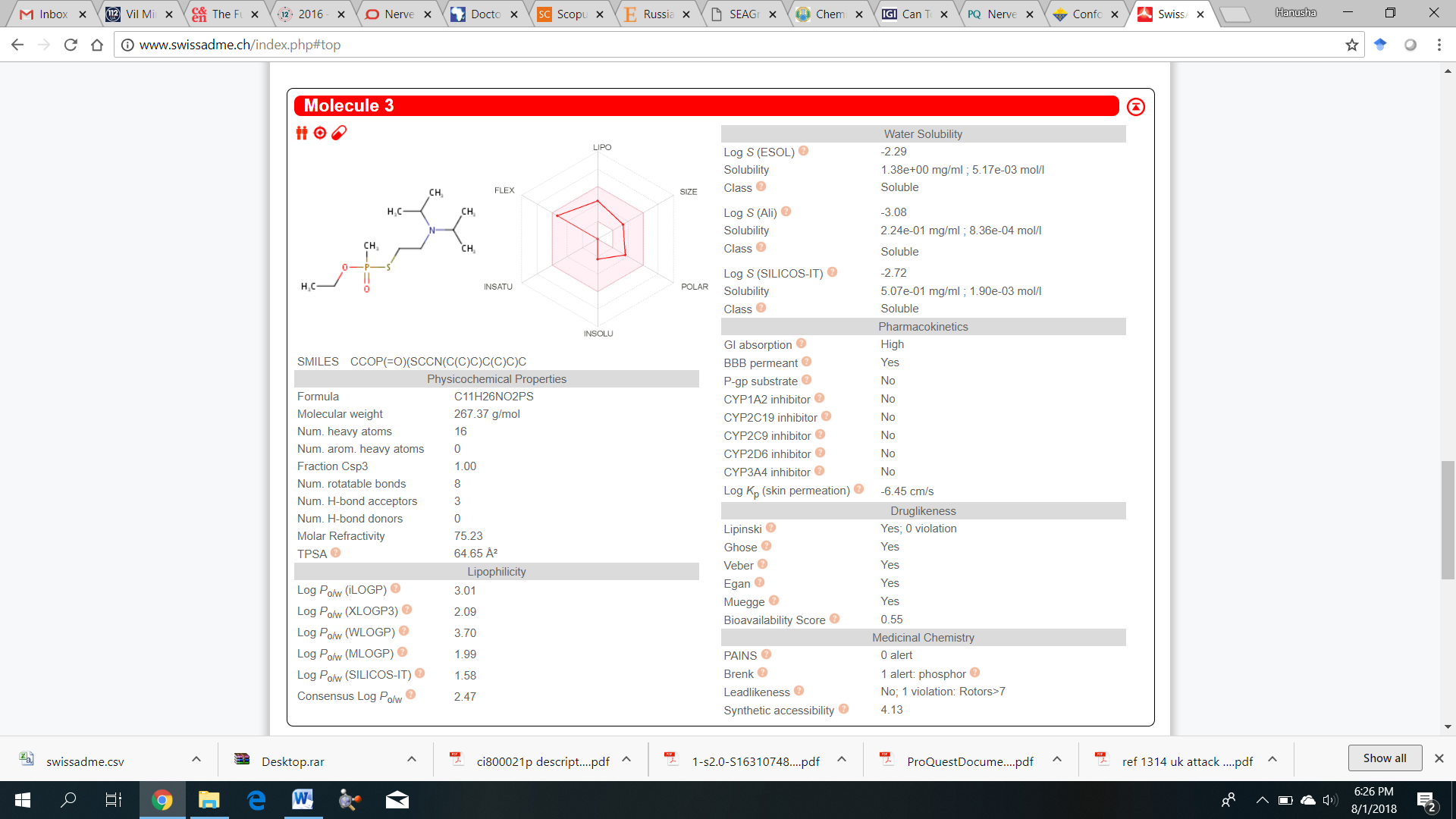


Figure S5d: Pharmacokinetics, drug-likeness and medicinal chemistry friendliness of VX evaluated using the free web tool SwissADME.


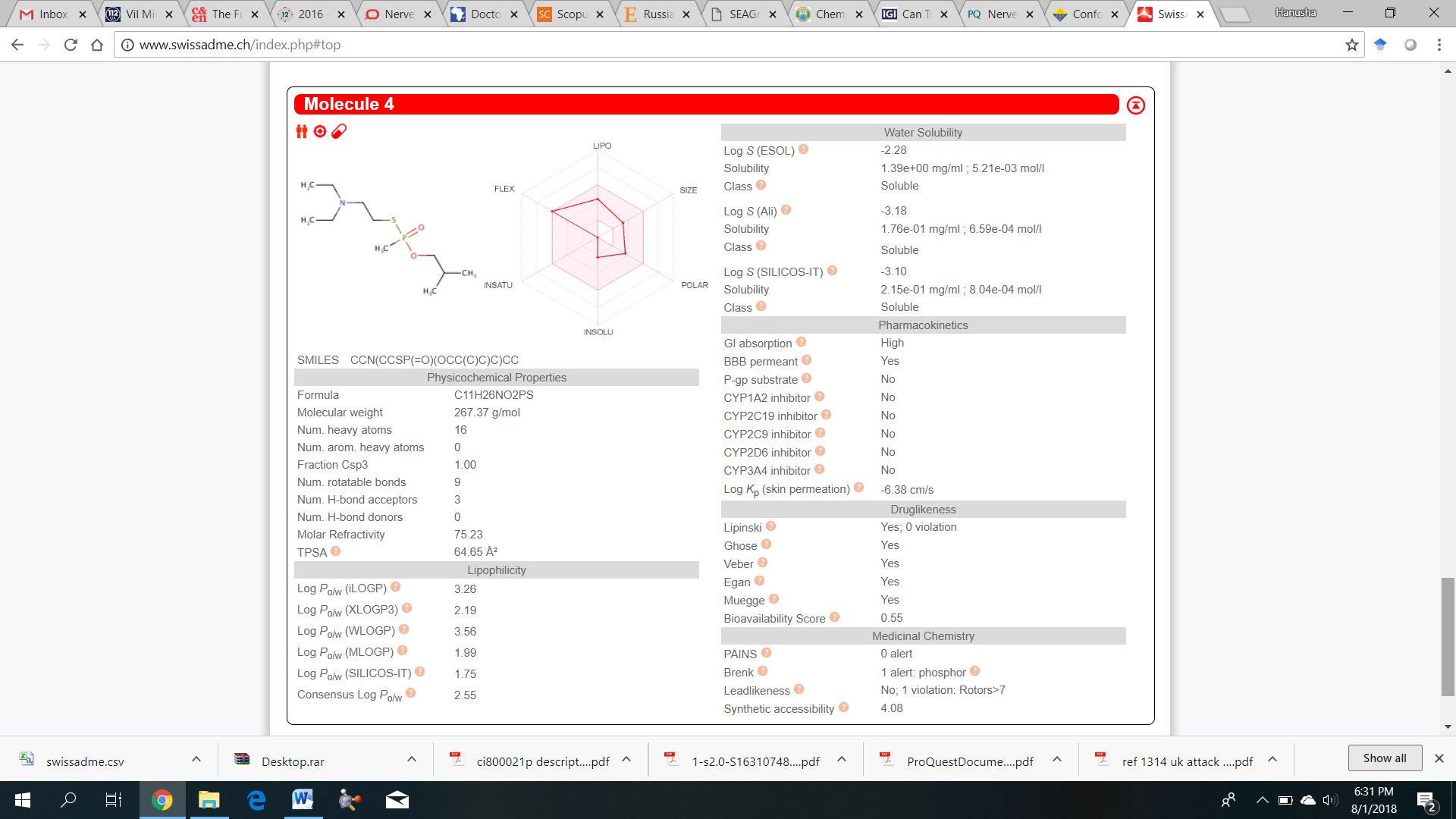


**Nerve agent poisoning and antidotes**


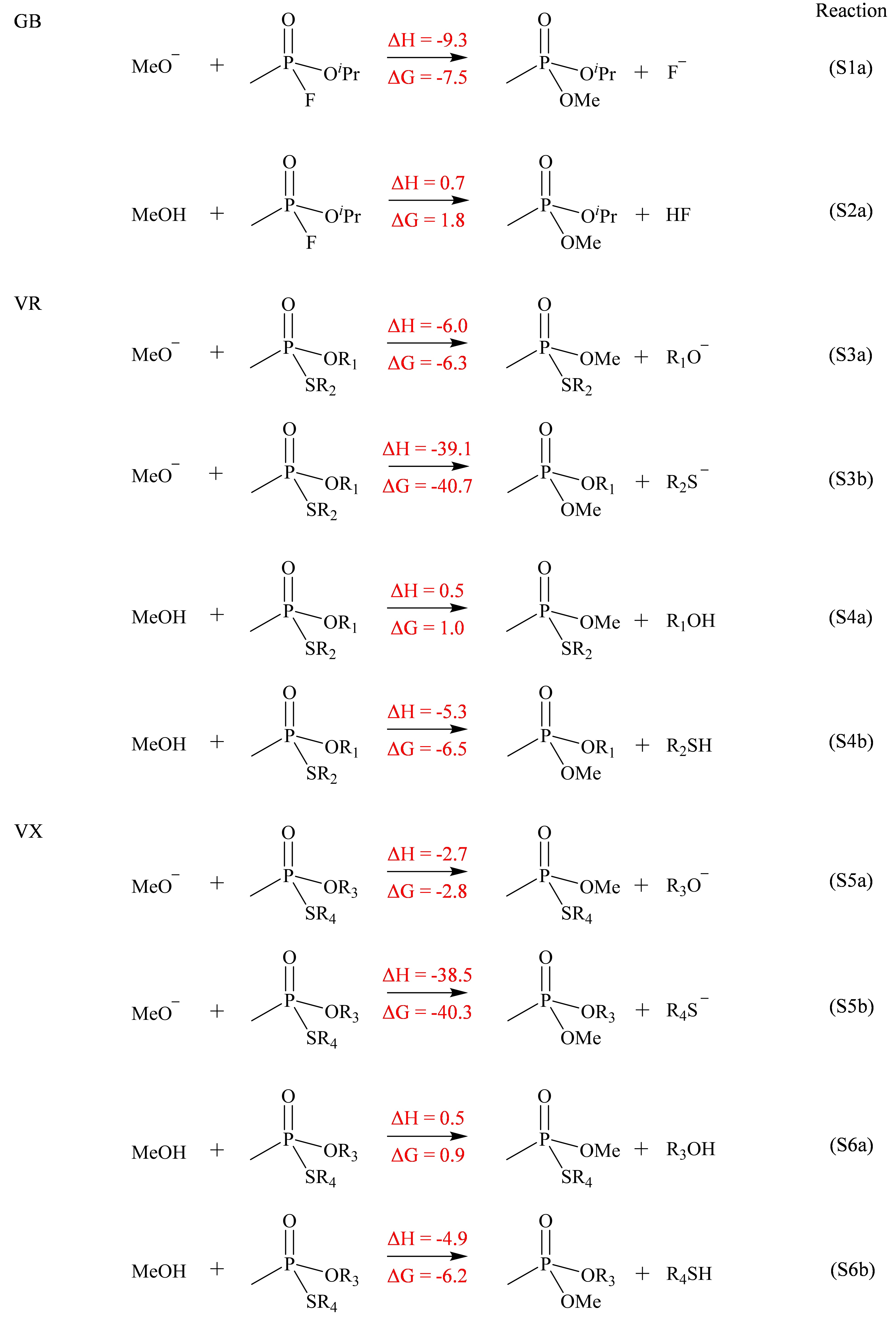


Scheme S1: The enthalpy (ΔH) and free energy change (ΔG) for the phosphonylation reaction between the nerve agents and AChE models (kcal/mol). R_1_, R_2_ R_3_, and R_4_ corresponds to CH_2_CHMe_2_, CH_2_CH_2_NEt_2_, Et, and CH_2_CH_2_N(*i*-Pr)_2_, respectively.


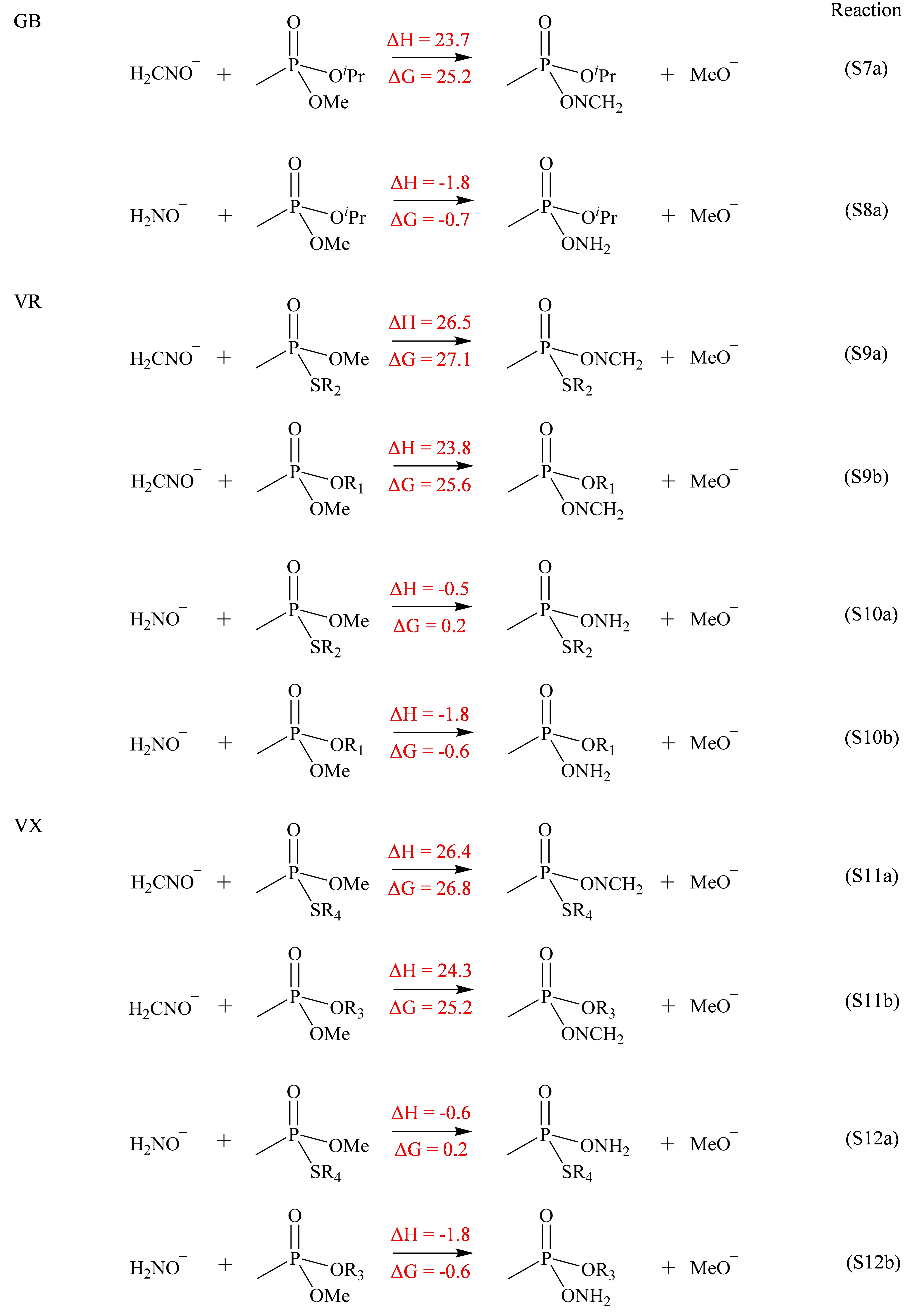


Scheme S2: The enthalpy (ΔH) and free energy change (ΔG) for the reactivation of nerve agent-inhibited AChE model induced by formoximate and hydroxylamine anions (kcal/mol). R_1_, R_2_ R_3_, and R_4_ correspond to CH_2_CHMe_2_, CH_2_CH_2_NEt_2_, Et, and CH_2_CH_2_N(*i*-Pr)_2_, respectively.

| 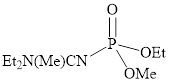 | 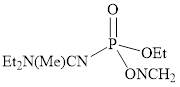 | 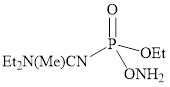 |
| --- | --- | --- |
| 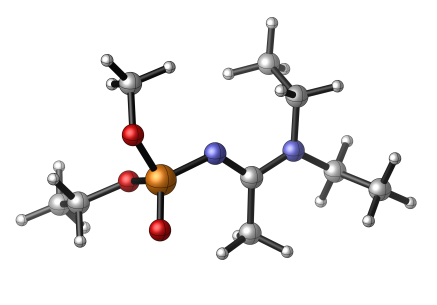 | 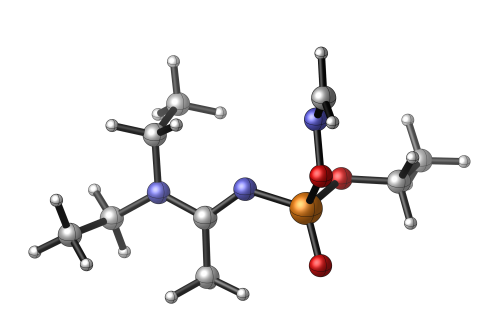 | 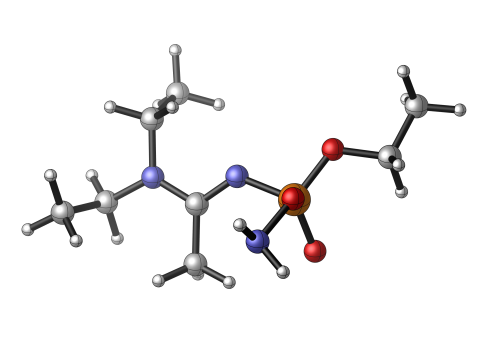 |
| A234-OMe_1 | A234-ONCH_2__1 | A234-ONH_2__1 |
|  |  |  |
| 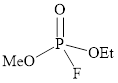 | 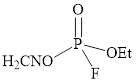 | 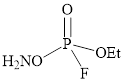 |
| 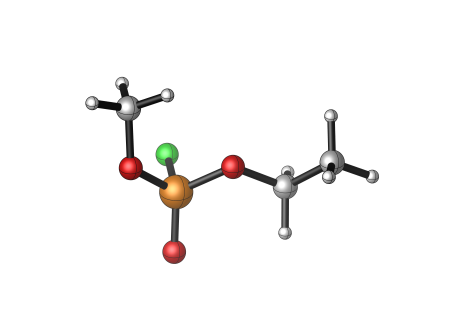 | 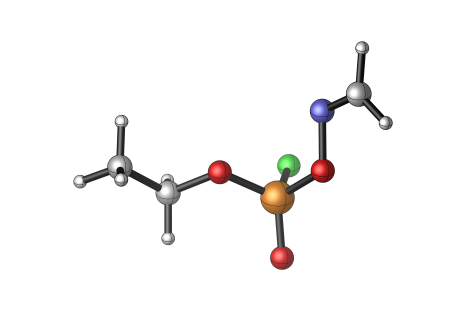 | 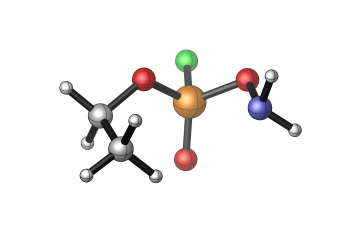 |
| A234-OMe_2 | A234-ONCH_2__2 | A234-ONH_2__2 |
|  |  |  |
| 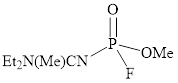 | 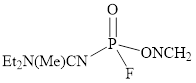 | 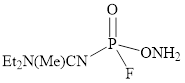 |
| 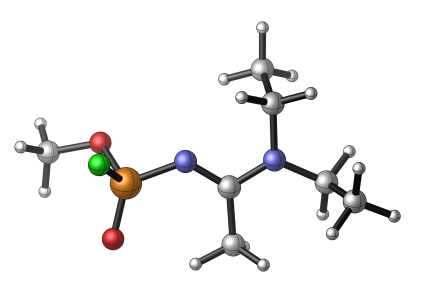 | 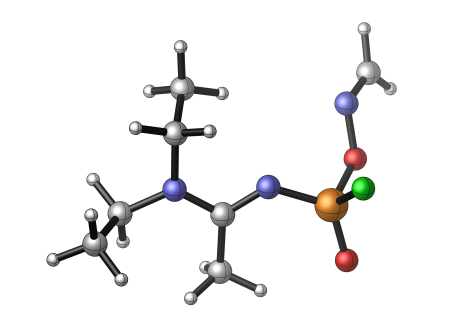 | 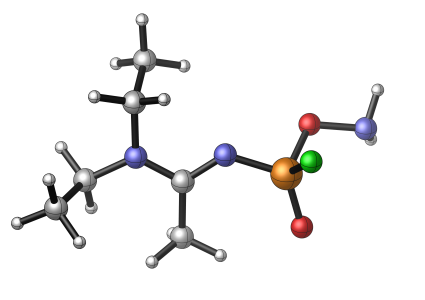 |
| A234-OMe_3 | A234-ONCH_2__3 | A234-ONH_2__3 |

Figure S6a: Optimised geometry of the products [M062X/6-311++G(d,p)] associated with (a) the phosphonylation reaction between A234 and AChE models and (b) the reactivation of A234-inhibited AChE model induced by formoximate and hydroxylamine anions.

| 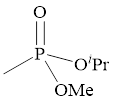 | 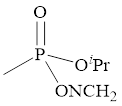 | 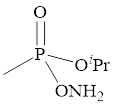 |
| --- | --- | --- |
| 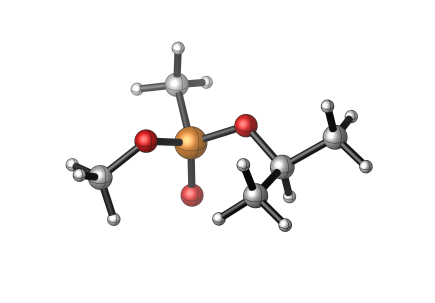 | 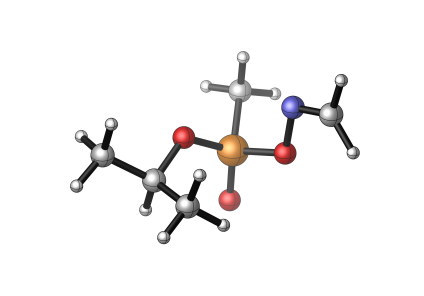 | 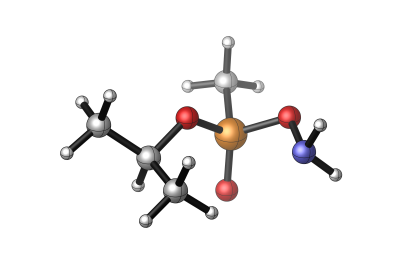 |
| GB-OMe | GB-ONCH_2_ | GB-ONH_2_ |

Figure S6b: Optimised geometry of the products [M062X/6-311++G(d,p)] associated with (a) the phosphonylation reaction between GB and AChE models and (b) the reactivation of GB-inhibited AChE model induced by formoximate and hydroxylamine anions.

| 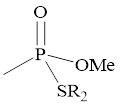 | 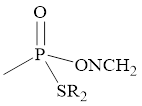 | 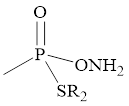 |
| --- | --- | --- |
| 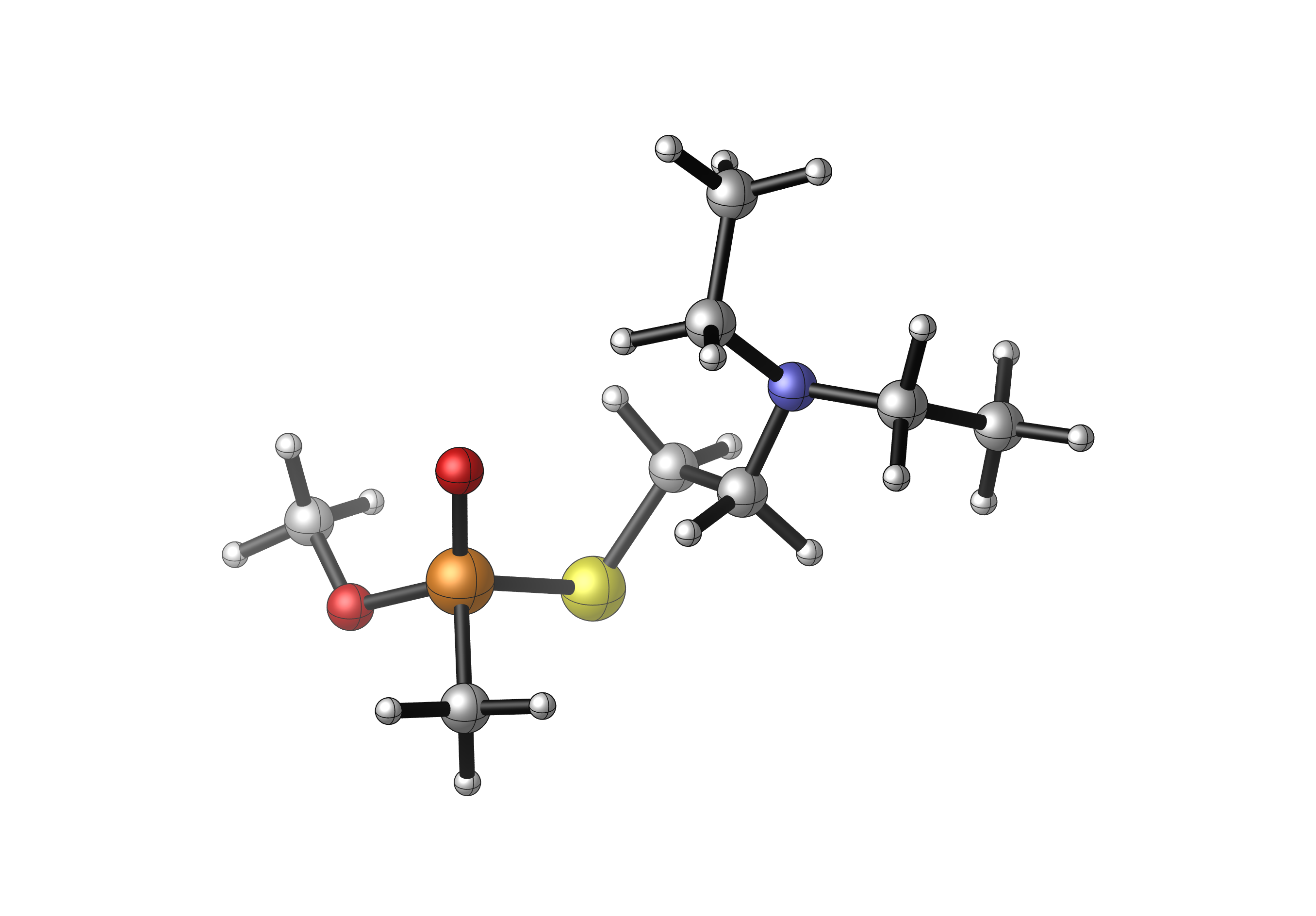 | 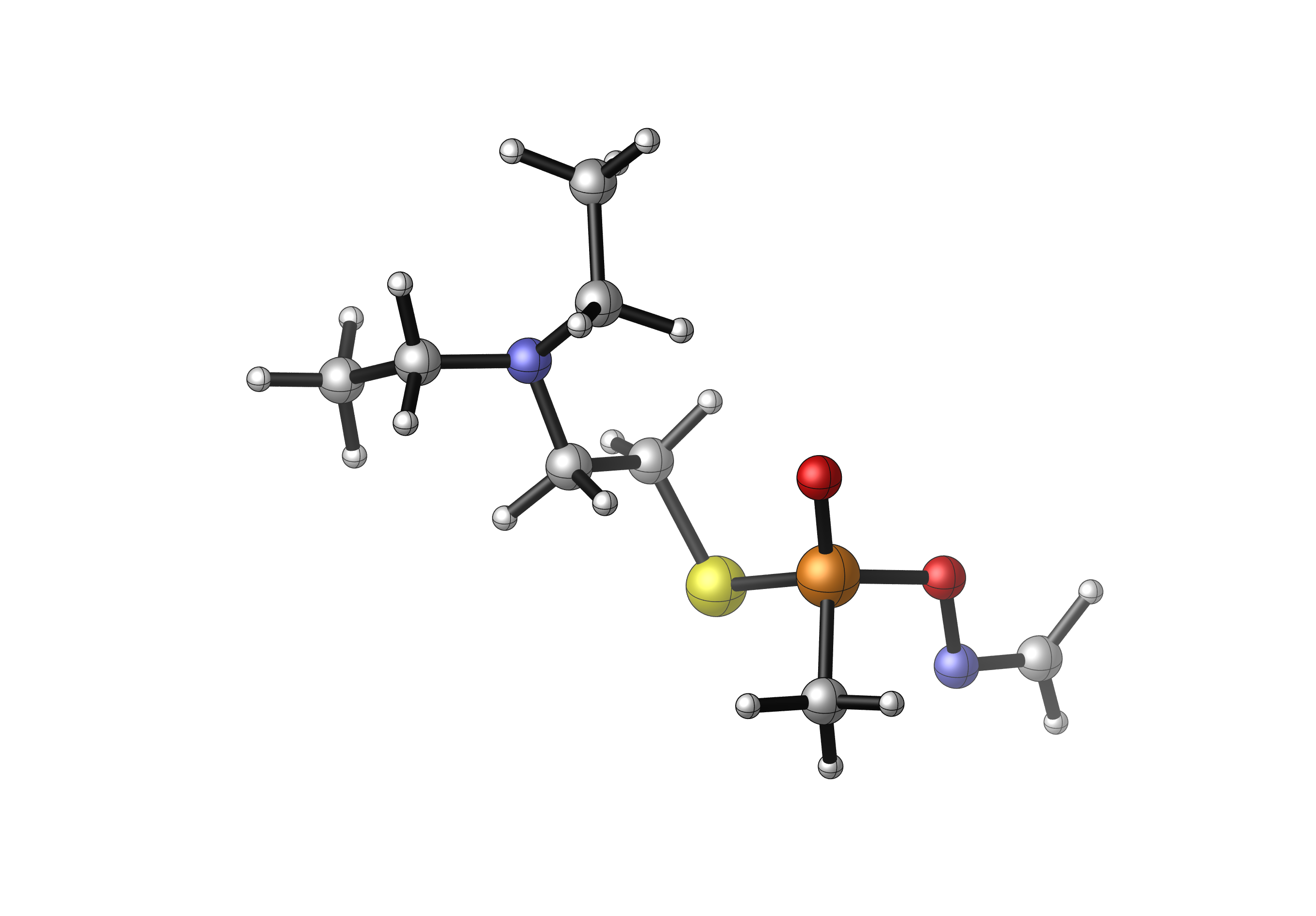 | 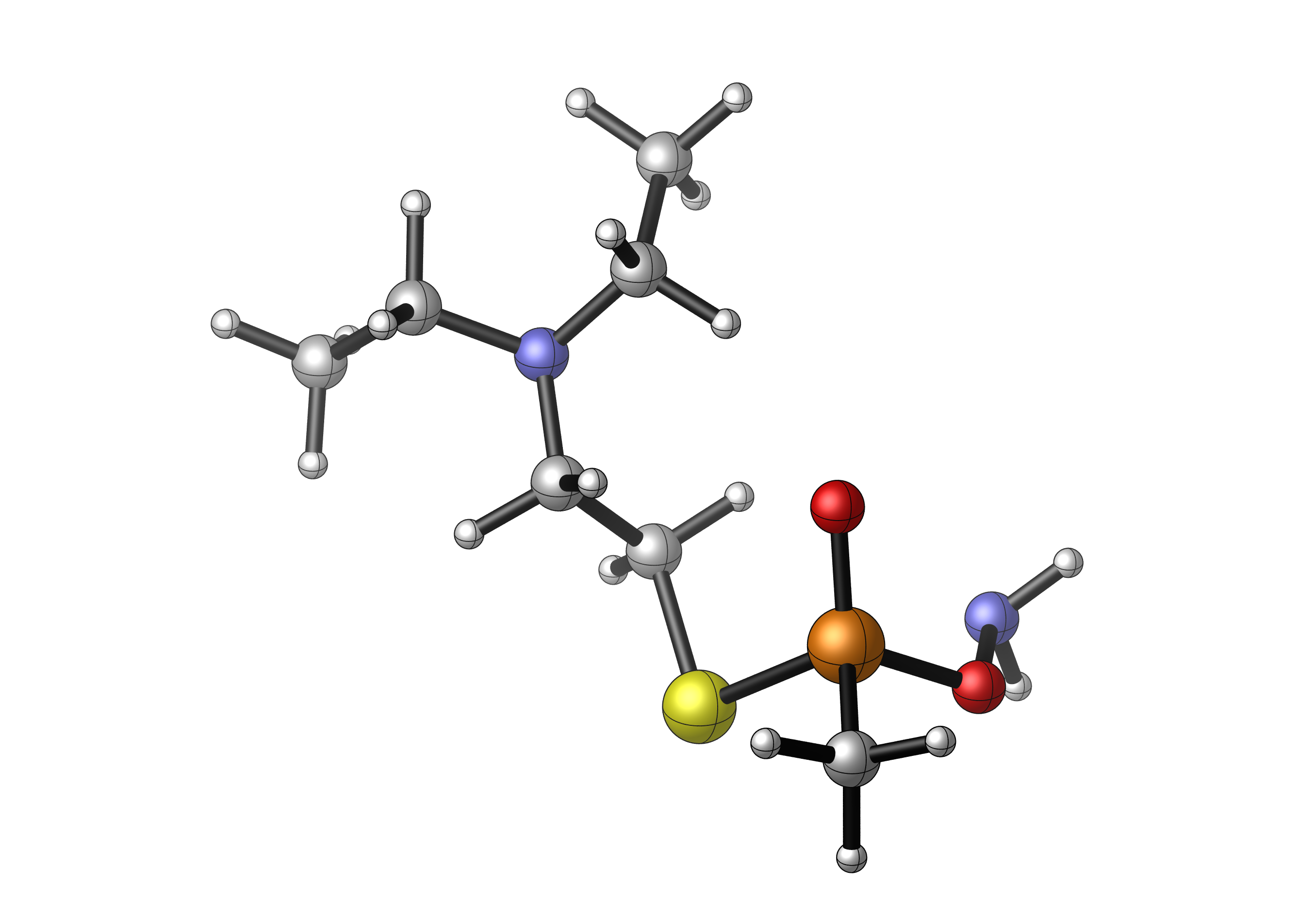 |
| VR-OMe_1 | VR-ONCH_2__1 | VR-ONH_2__1 |
|  |  |  |
| 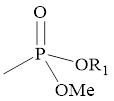 | 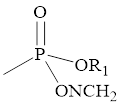 | 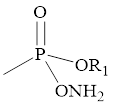 |
| 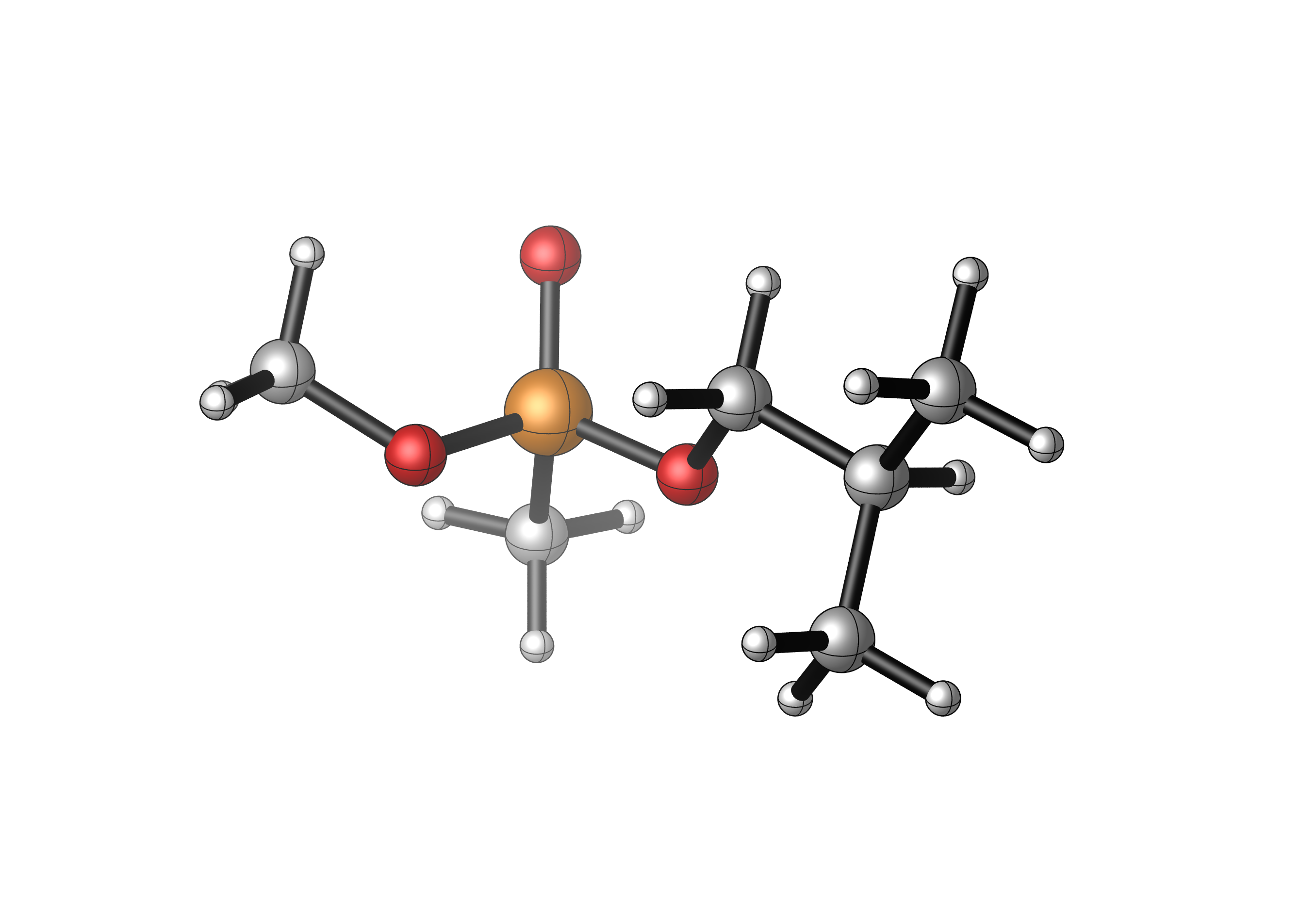 | 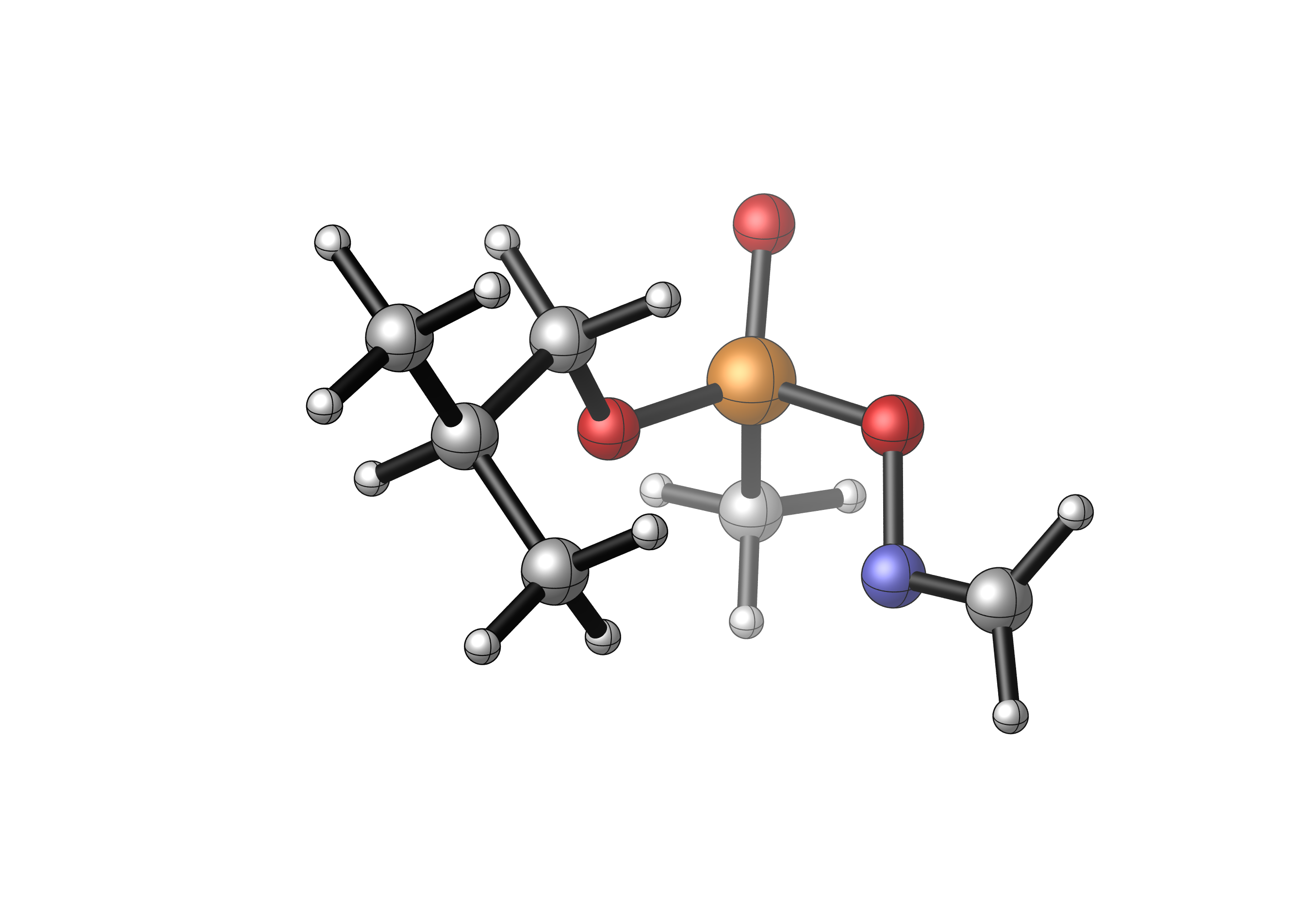 | 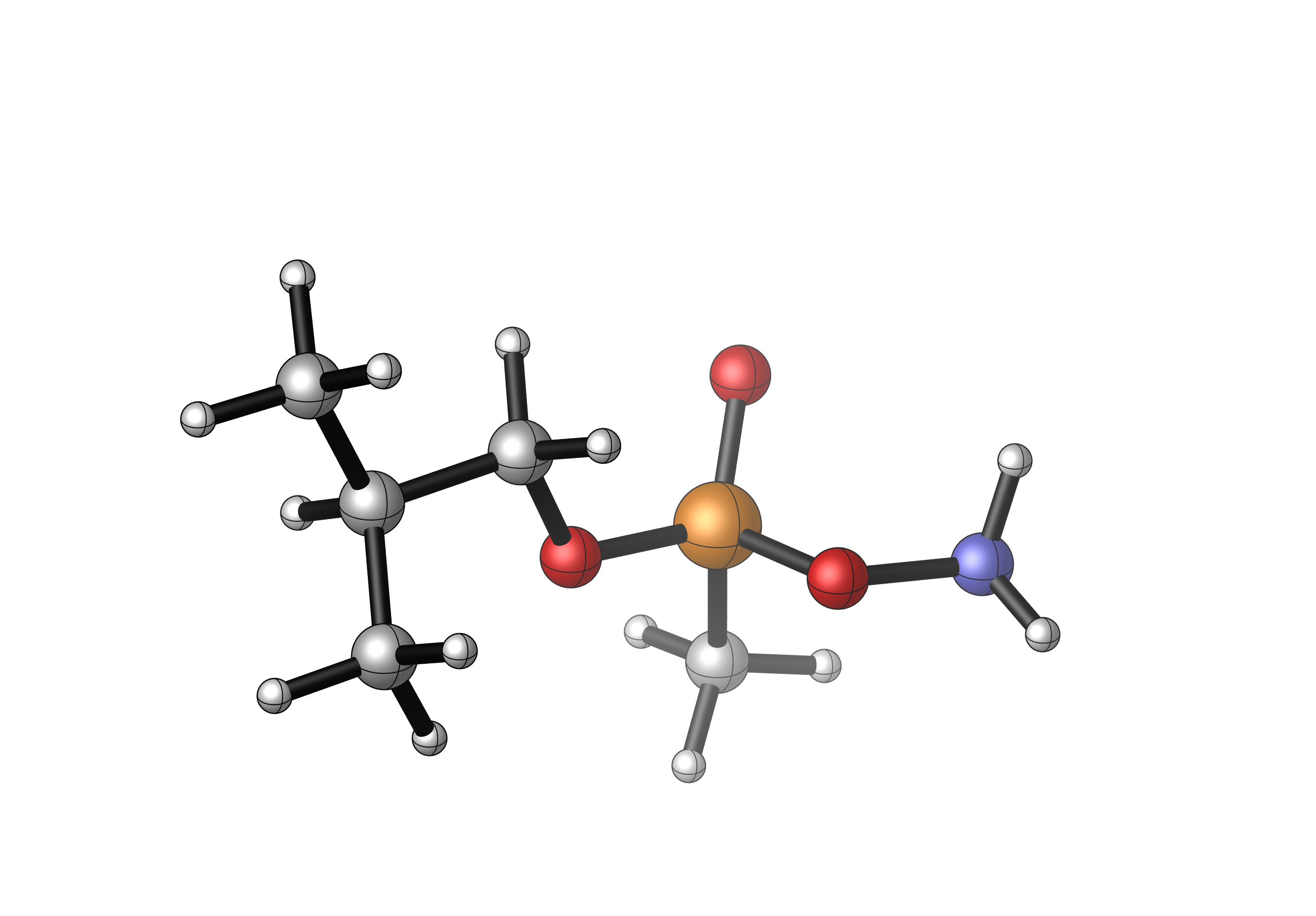 |
| VR-OMe_2 | VR-ONCH_2__2 | VR-ONH_2__2 |

Figure S6c: Optimised geometry of the products [M062X/6-311++G(d,p)] associated with (a) the phosphonylation reaction between VR and AChE models and (b) the reactivation of VR-inhibited AChE model induced by formoximate and hydroxylamine anions.

| 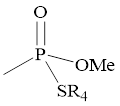 | 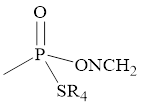 | 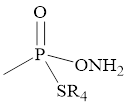 |
| --- | --- | --- |
| 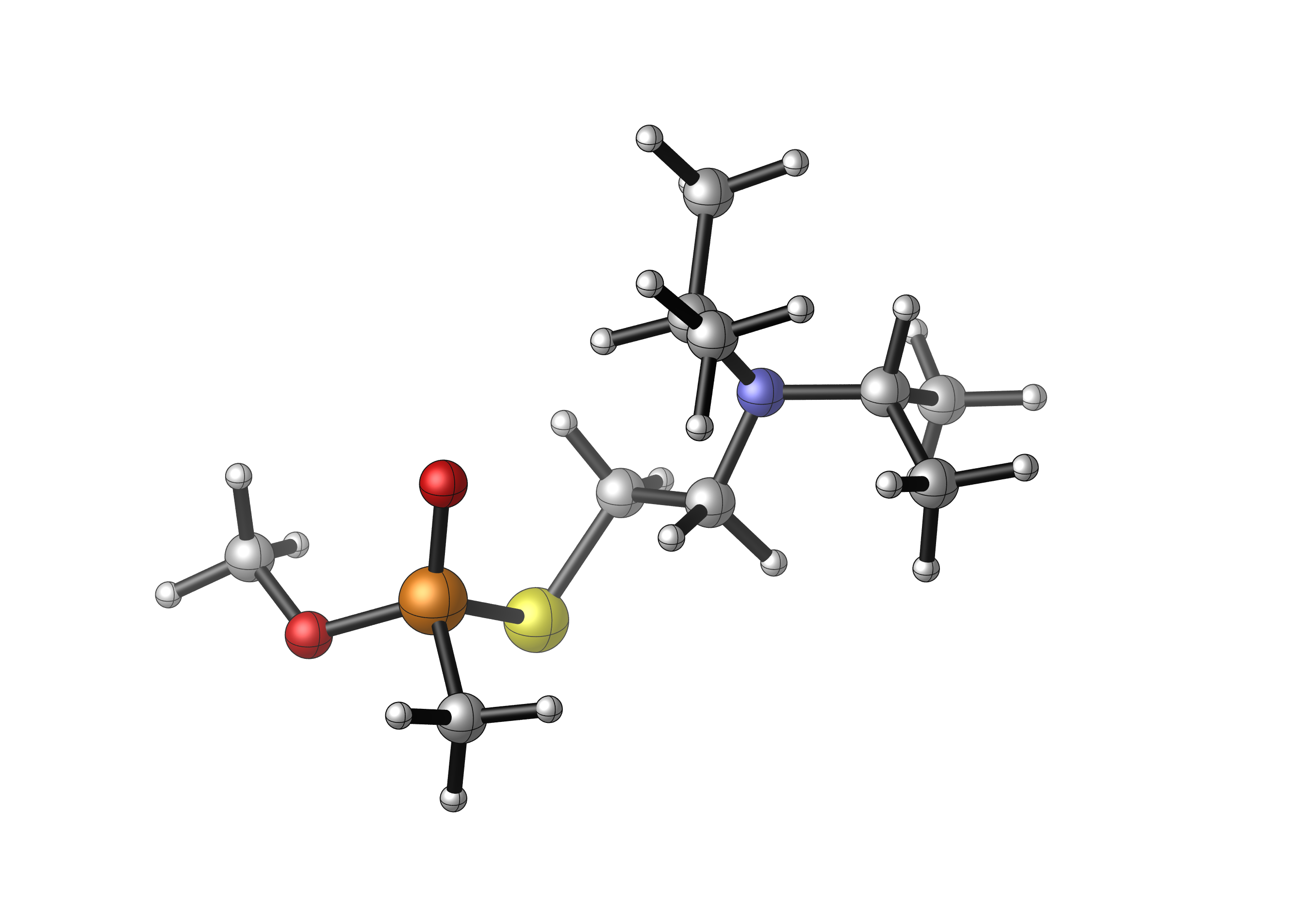 | 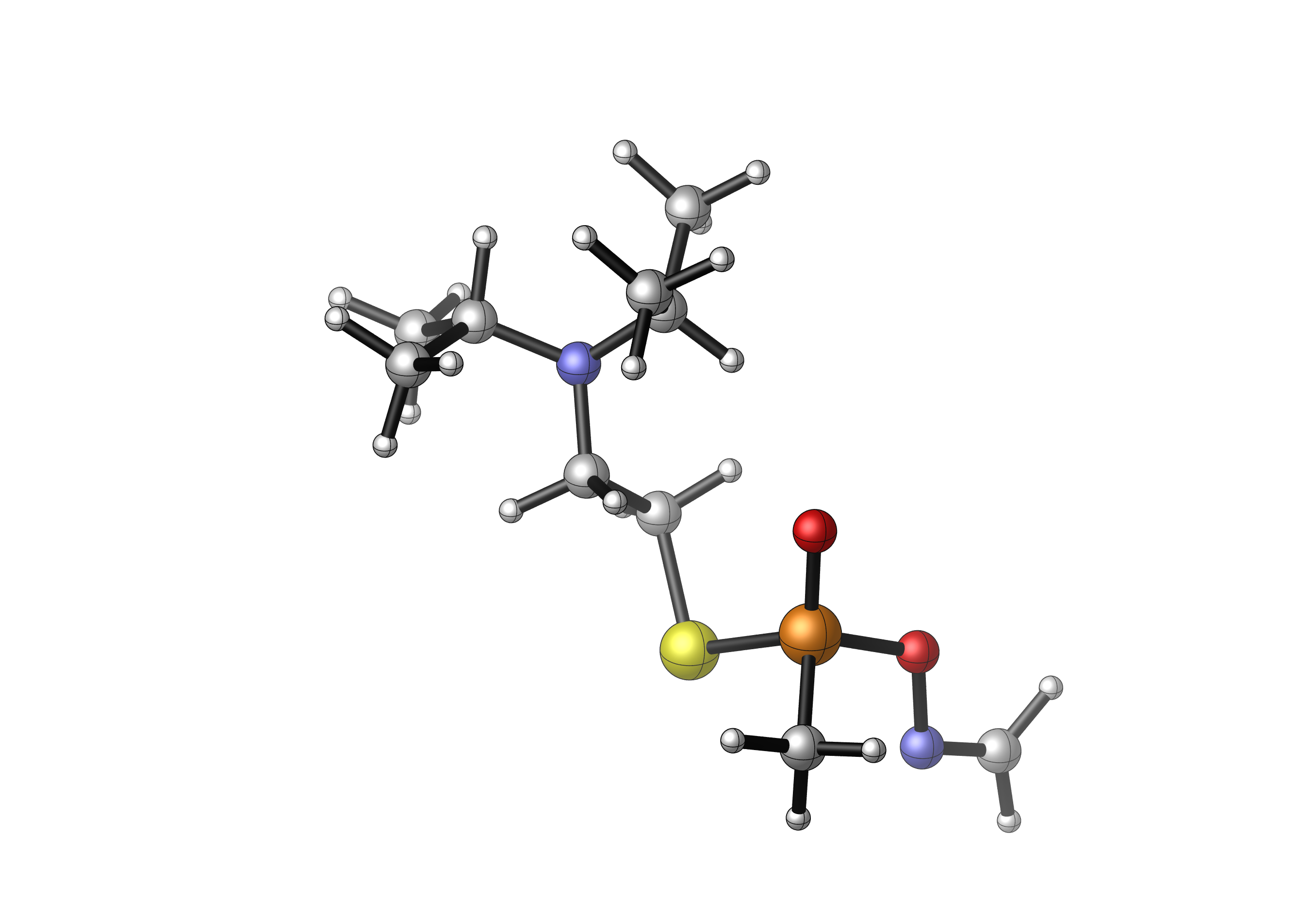 | 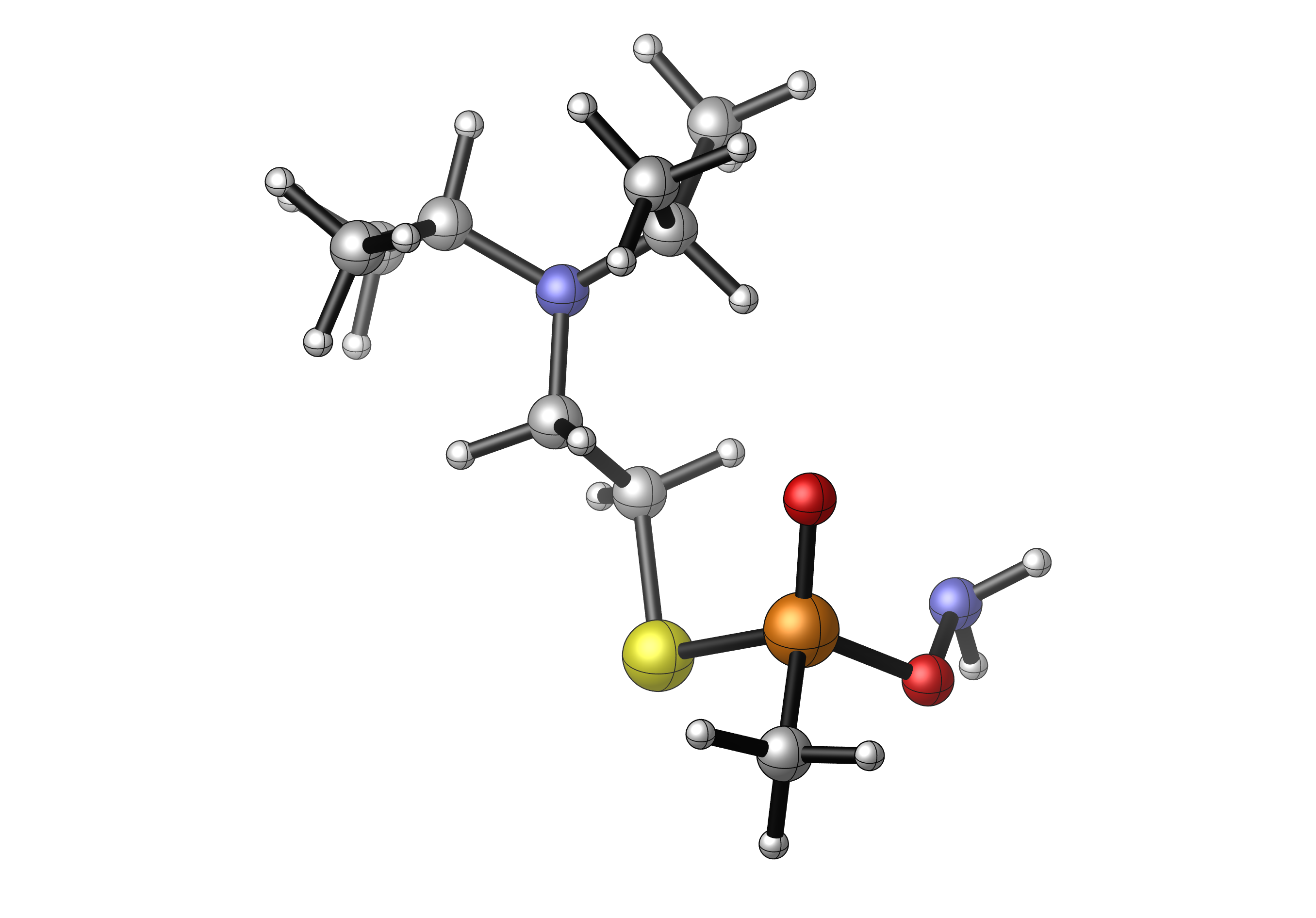 |
| VX-OMe_1 | VX-ONCH_2__1 | VX-ONH_2__1 |
|  |  |  |
| 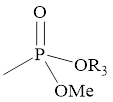 | 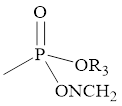 | 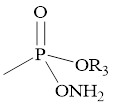 |
| 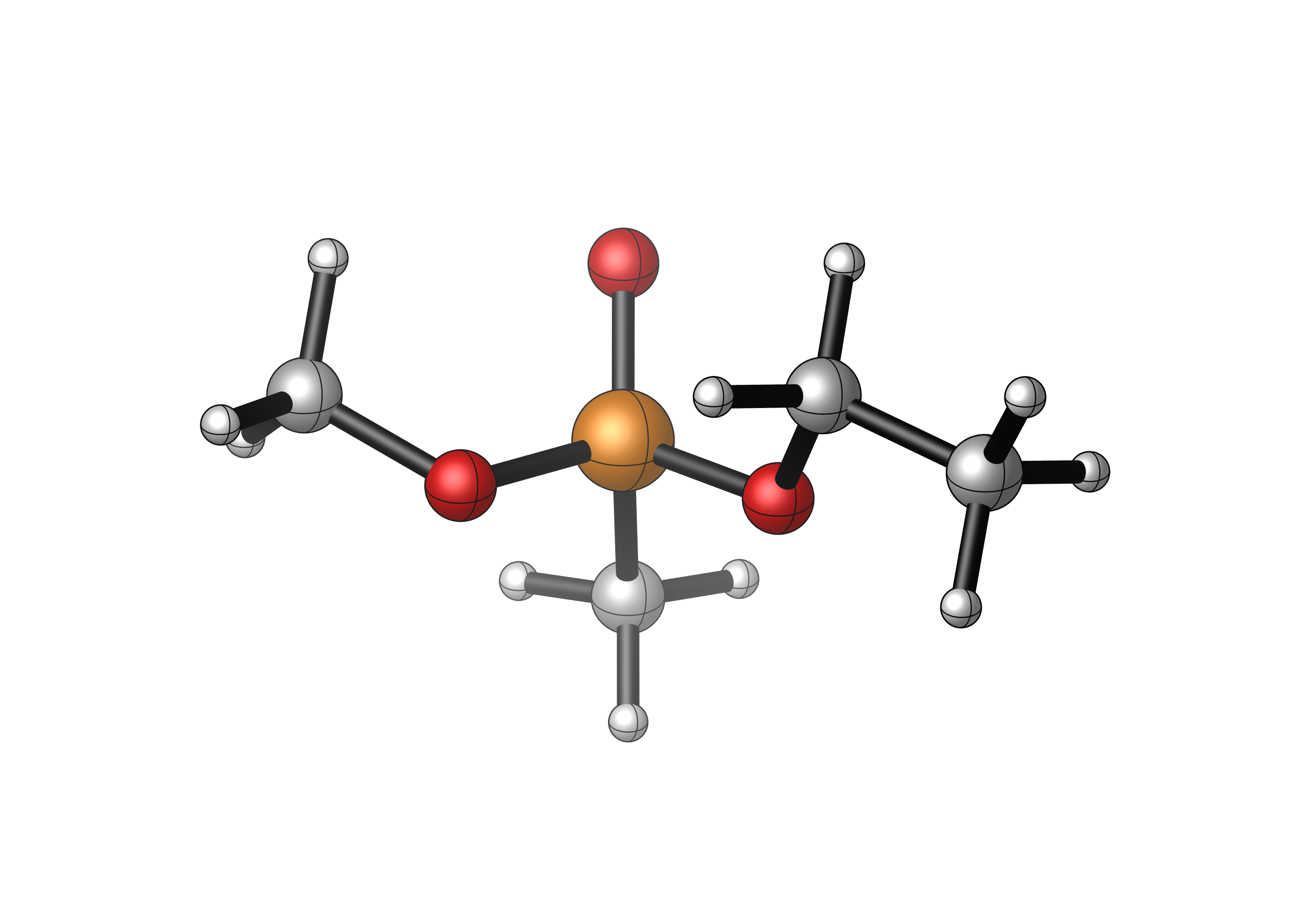 | 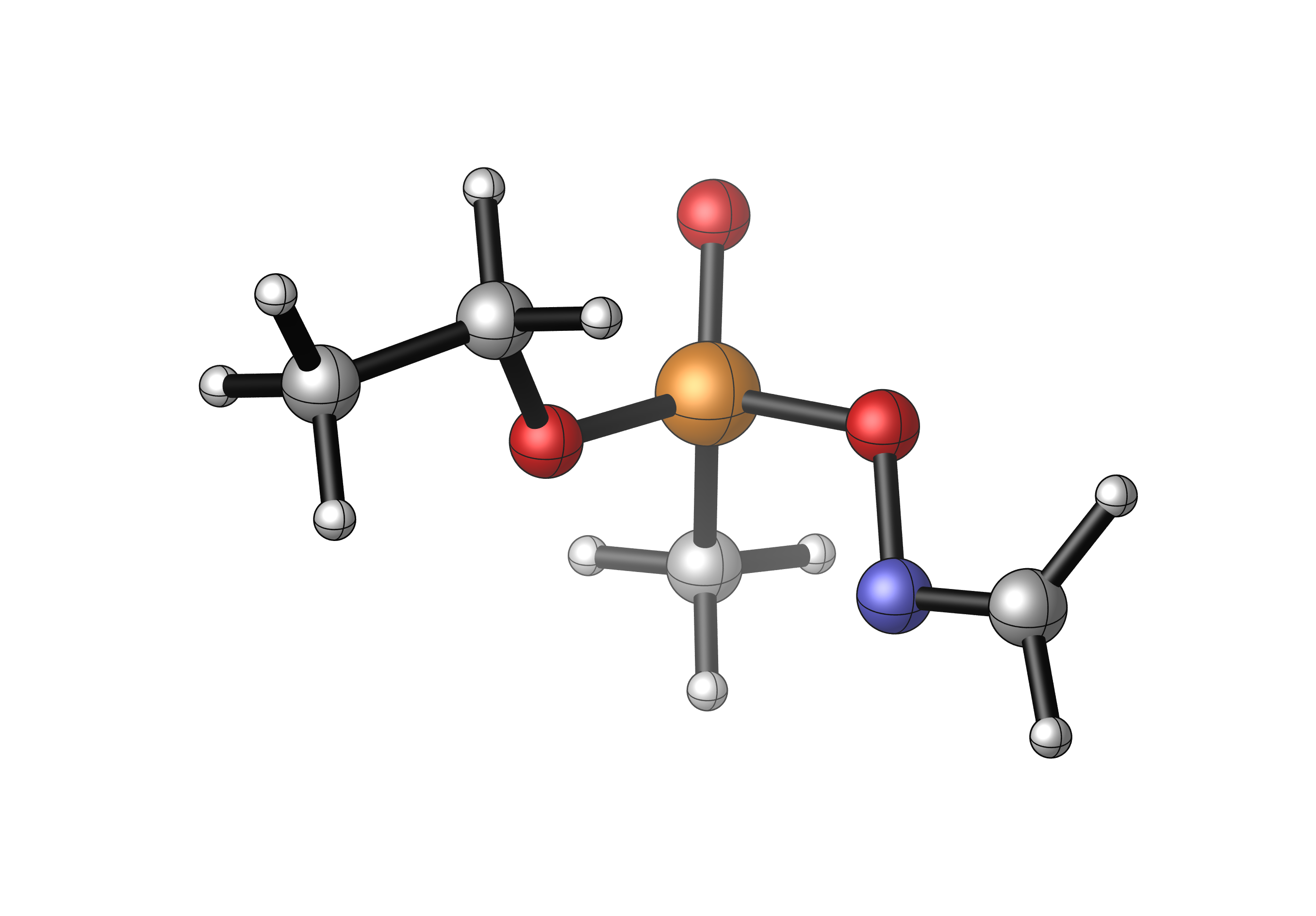 | 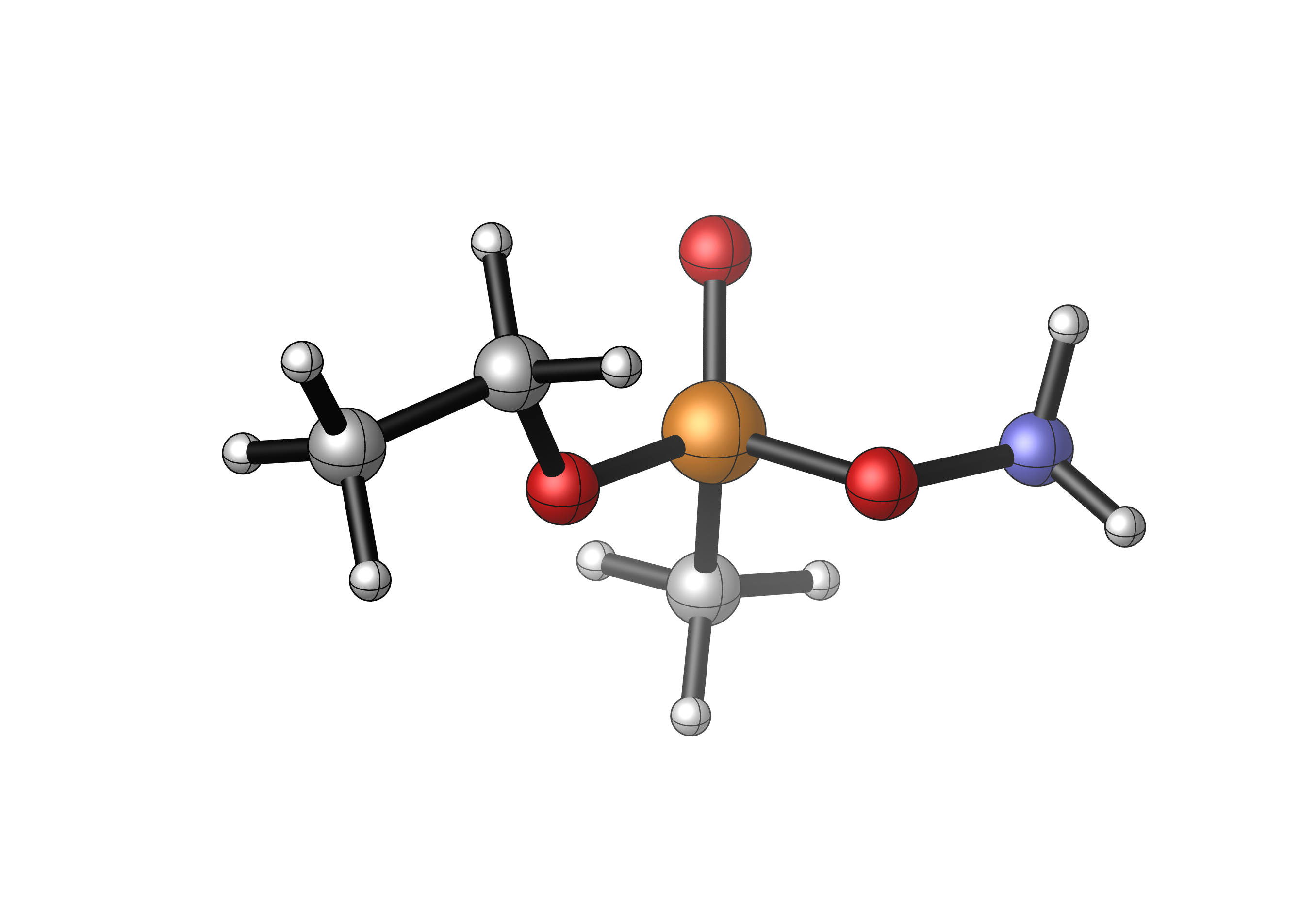 |
| VX-OMe_2 | VX-ONCH_2__2 | VX-ONH_2__2 |

Figure S6d: Optimised geometry of the products [M062X/6-311++G(d,p)] associated with (a) the phosphonylation reaction between VX and AChE models and (b) the reactivation of VX-inhibited AChE model induced by formoximate and hydroxylamine anions.

**Heat of formation**

The enthalpies of formation (Δ_f_$H_{298}^{^{\circ}}$) of the nerve agents were calculated as per the following steps. The atomisation energies (AEs) were calculated using the B3LYP/6-311G(2d,d,p) (as implemented in the CBS-QB3 composite method) optimised geometries, taking into consideration the spin-orbit effects.^S6-S8^ Using the AEs and the Δ_f_$H_{298}^{^{\circ}}$ of the individual atoms (obtained from literature^S9^), the Δ_f_$H_{298}^{^{\circ}}$ of each nerve agent was calculated. Relevant data are provided in Table S6. Taking novichok A234 (C_8_H_18_FN_2_O_2_P) as an example, the heat of formation was estimated as follows:

AE(C_8_H_18_FN_2_O_2_P) = [8E_CBS+SSC_(C) + 18E_CBS+SSC_(H) + ΔE_CBS+SSC_(F) + 2ΔE_CBS+SSC_(N) +
 2E_CBS+SSC_(O) + ΔE_CBS+SSC_(P)] – E_CBS_(C_8_H_18_FN_2_O_2_P)

where E_CBS+SSC_ of each individual atom corresponds to their spin splitting corrected CBS-QB3 enthalpy.

Δ_f_$H_{298}^{^{\circ}}$(C_8_H_18_FN_2_O_2_P) = [8Δ_f_$H_{298}^{^{\circ}}$(C) + 18Δ_f_$H_{298}^{^{\circ}}$(H) + Δ_f_$H_{298}^{^{\circ}}$(F) + 2Δ_f_$H_{298}^{^{\circ}}$(N) + 2Δ_f_$H_{298}^{^{\circ}}$(O) + Δ_f_$H_{298}^{^{\circ}}$(P)] – AE(C_8_H_18_FN_2_O_2_P)

Table S6: Energies associated with the calculation of enthalpy of formation of the studied species.

|  | CBS-QB3 Enthalpy  (Hartree) at 298 K | Spin Splitting  Corrections (Hartree)^S6-S8^ | Δ_f_$H_{298}^{^{\circ}}$ Experimental  (kcal/mol)^S9^ |
| --- | --- | --- | --- |
| C | -37.78302 | 0.00014 | 171.29 |
| H | -0.49746 | 0.00000 | 52.1 |
| F | -99.64072 | 0.00061 | 18.97 |
| N | -54.51818 | 0.00000 | 112.97 |
| O | -74.98527 | 0.00036 | 59.6 |
| P | -340.81482 | 0.00000 | 75.65 |
| S | -397.65500 | 0.00089 | 66.24 |
|  |  |  |  |
| A234 | -1015.44175 |  |  |
| GB | -749.18073 |  |  |
| VR | -1377.55231 |  |  |
| VX | -1377.55227 |  |  |

Table S7: Cartesian coordinates (in Å) and total electronic energies (in Hartree) of the gas-phase optimised geometries of A234 obtained using the B3LYP/6-311++G(d,p) method.

| A234  Conformers | Total Electronic  Energy (Hartree) | Cartesian Coordinates (in Å) | | | |
| --- | --- | --- | --- | --- | --- |
| **1** | -1017.16642 | 7 | 2.284116 | 0.318071 | 0.081343 |
|  |  | 6 | 1.172412 | -0.435374 | 0.263581 |
|  |  | 7 | 0.070287 | -0.027522 | -0.302517 |
|  |  | 15 | -1.399003 | -0.721606 | -0.310673 |
|  |  | 8 | -1.706812 | -1.993979 | 0.386565 |
|  |  | 9 | -1.688292 | -0.877814 | -1.885709 |
|  |  | 8 | -2.362128 | 0.505974 | 0.067128 |
|  |  | 6 | 2.214064 | 1.537220 | -0.742584 |
|  |  | 6 | 1.781004 | 2.768253 | 0.055638 |
|  |  | 1 | 1.512669 | 1.352765 | -1.554763 |
|  |  | 1 | 3.205200 | 1.690034 | -1.176866 |
|  |  | 1 | 1.776936 | 3.649846 | -0.591427 |
|  |  | 1 | 0.773330 | 2.628681 | 0.449612 |
|  |  | 1 | 2.461273 | 2.966837 | 0.889060 |
|  |  | 6 | 3.589054 | 0.008846 | 0.676188 |
|  |  | 6 | 4.527785 | -0.749316 | -0.267686 |
|  |  | 1 | 3.445373 | -0.552923 | 1.597638 |
|  |  | 1 | 4.043923 | 0.960198 | 0.966434 |
|  |  | 1 | 5.496136 | -0.909168 | 0.214672 |
|  |  | 1 | 4.116256 | -1.724624 | -0.537674 |
|  |  | 1 | 4.700397 | -0.190537 | -1.190669 |
|  |  | 6 | 1.297284 | -1.676100 | 1.124916 |
|  |  | 1 | 2.141679 | -2.294484 | 0.815598 |
|  |  | 1 | 1.453980 | -1.391195 | 2.169827 |
|  |  | 1 | 0.382200 | -2.260328 | 1.065495 |
|  |  | 6 | -3.786336 | 0.283790 | 0.242854 |
|  |  | 6 | -4.378798 | 1.535729 | 0.856066 |
|  |  | 1 | -3.931291 | -0.589364 | 0.883423 |
|  |  | 1 | -4.226833 | 0.075083 | -0.736761 |
|  |  | 1 | -5.456666 | 1.406893 | 0.989583 |
|  |  | 1 | -3.931458 | 1.737393 | 1.831863 |
|  |  | 1 | -4.211540 | 2.401220 | 0.211260 |
|  |  |  |  |  |  |
|  |  |  |  |  |  |
| **2** | -1017.16649 | 7 | 2.292193 | -0.224560 | -0.084866 |
|  |  | 6 | 1.182570 | 0.532092 | 0.096183 |
|  |  | 7 | 0.045901 | -0.091748 | 0.239559 |
|  |  | 15 | -1.443142 | 0.529203 | 0.433750 |
|  |  | 8 | -1.766226 | 1.958964 | 0.207316 |
|  |  | 9 | -1.797402 | 0.122109 | 1.952233 |
|  |  | 8 | -2.348907 | -0.507145 | -0.390292 |
|  |  | 6 | 2.175124 | -1.692325 | -0.133969 |
|  |  | 6 | 2.228736 | -2.339460 | 1.250791 |
|  |  | 1 | 2.990997 | -2.059862 | -0.761620 |
|  |  | 1 | 1.233938 | -1.936258 | -0.624379 |
|  |  | 1 | 2.168462 | -3.427283 | 1.156503 |
|  |  | 1 | 3.160055 | -2.099304 | 1.772135 |
|  |  | 1 | 1.388896 | -2.001971 | 1.859734 |
|  |  | 6 | 3.647423 | 0.325231 | -0.202056 |
|  |  | 6 | 4.118119 | 0.489308 | -1.650519 |
|  |  | 1 | 4.317851 | -0.354799 | 0.331191 |
|  |  | 1 | 3.700798 | 1.277195 | 0.323946 |
|  |  | 1 | 5.147028 | 0.859077 | -1.671825 |
|  |  | 1 | 4.091557 | -0.462391 | -2.186436 |
|  |  | 1 | 3.489104 | 1.198334 | -2.193768 |
|  |  | 6 | 1.354942 | 2.036974 | 0.139752 |
|  |  | 1 | 0.382967 | 2.520623 | 0.072379 |
|  |  | 1 | 1.817793 | 2.329757 | 1.087522 |
|  |  | 1 | 1.991720 | 2.394781 | -0.670496 |
|  |  | 6 | -3.766390 | -0.249086 | -0.569300 |
|  |  | 6 | -4.285625 | -1.218124 | -1.611340 |
|  |  | 1 | -3.899525 | 0.790104 | -0.879003 |
|  |  | 1 | -4.268231 | -0.393930 | 0.392238 |
|  |  | 1 | -5.357366 | -1.059673 | -1.761302 |
|  |  | 1 | -3.777040 | -1.068130 | -2.566188 |
|  |  | 1 | -4.130251 | -2.251346 | -1.293271 |
|  |  |  |  |  |  |
|  |  |  |  |  |  |
| **3** | -1017.16593 | 7 | -2.340834 | 0.183391 | -0.067282 |
|  |  | 6 | -1.190747 | -0.475748 | 0.216310 |
|  |  | 7 | -0.072185 | 0.180668 | 0.081756 |
|  |  | 15 | 1.452873 | -0.327967 | 0.320500 |
|  |  | 8 | 1.813797 | -1.745463 | 0.570230 |
|  |  | 9 | 1.915092 | 0.584039 | 1.563749 |
|  |  | 8 | 2.241112 | 0.355195 | -0.897436 |
|  |  | 6 | -2.286052 | 1.582959 | -0.523739 |
|  |  | 6 | -2.240605 | 2.582831 | 0.632927 |
|  |  | 1 | -3.168696 | 1.753924 | -1.145226 |
|  |  | 1 | -1.402660 | 1.696411 | -1.150569 |
|  |  | 1 | -2.230440 | 3.604520 | 0.243149 |
|  |  | 1 | -3.111743 | 2.481529 | 1.286902 |
|  |  | 1 | -1.337232 | 2.433843 | 1.225829 |
|  |  | 6 | -3.677272 | -0.403981 | 0.078229 |
|  |  | 6 | -4.239164 | -0.976524 | -1.226840 |
|  |  | 1 | -4.336553 | 0.384748 | 0.451504 |
|  |  | 1 | -3.655945 | -1.171199 | 0.850752 |
|  |  | 1 | -5.253114 | -1.353513 | -1.066600 |
|  |  | 1 | -4.283111 | -0.213669 | -2.007895 |
|  |  | 1 | -3.623381 | -1.799969 | -1.596096 |
|  |  | 6 | -1.299484 | -1.912559 | 0.685631 |
|  |  | 1 | -0.315344 | -2.375482 | 0.684124 |
|  |  | 1 | -1.688112 | -1.941007 | 1.708461 |
|  |  | 1 | -1.972850 | -2.493490 | 0.053675 |
|  |  | 6 | 3.572094 | -0.091346 | -1.271834 |
|  |  | 6 | 4.650806 | 0.655370 | -0.506226 |
|  |  | 1 | 3.635901 | 0.112324 | -2.341566 |
|  |  | 1 | 3.639938 | -1.169583 | -1.111821 |
|  |  | 1 | 5.635407 | 0.353507 | -0.876441 |
|  |  | 1 | 4.546740 | 1.734223 | -0.641941 |
|  |  | 1 | 4.606673 | 0.433768 | 0.562098 |
|  |  |  |  |  |  |
|  |  |  |  |  |  |
| **4** | -1017.16585 | 7 | -2.335275 | 0.235600 | -0.066885 |
|  |  | 6 | -1.181587 | -0.463761 | -0.199739 |
|  |  | 7 | -0.084509 | 0.099614 | 0.222783 |
|  |  | 15 | 1.433970 | -0.479979 | 0.233441 |
|  |  | 8 | 1.793211 | -1.846279 | -0.220645 |
|  |  | 9 | 1.837189 | -0.288807 | 1.777085 |
|  |  | 8 | 2.268185 | 0.696388 | -0.471147 |
|  |  | 6 | -3.641738 | -0.245381 | -0.529787 |
|  |  | 6 | -4.477805 | -0.904208 | 0.571948 |
|  |  | 1 | -3.503058 | -0.934045 | -1.361769 |
|  |  | 1 | -4.177368 | 0.617565 | -0.935692 |
|  |  | 1 | -5.454127 | -1.198795 | 0.177058 |
|  |  | 1 | -3.985085 | -1.796928 | 0.964177 |
|  |  | 1 | -4.645534 | -0.219278 | 1.406430 |
|  |  | 6 | -2.312142 | 1.570113 | 0.556560 |
|  |  | 6 | -1.997332 | 2.687042 | -0.439868 |
|  |  | 1 | -1.565774 | 1.555925 | 1.349462 |
|  |  | 1 | -3.291672 | 1.728709 | 1.014668 |
|  |  | 1 | -2.025095 | 3.656319 | 0.065782 |
|  |  | 1 | -1.000195 | 2.549268 | -0.860356 |
|  |  | 1 | -2.724322 | 2.714045 | -1.257006 |
|  |  | 6 | -1.259493 | -1.833204 | -0.844240 |
|  |  | 1 | -1.469167 | -1.731144 | -1.913555 |
|  |  | 1 | -0.308380 | -2.348384 | -0.732620 |
|  |  | 1 | -2.052323 | -2.439363 | -0.402982 |
|  |  | 6 | 3.612111 | 0.465384 | -0.973115 |
|  |  | 6 | 4.660897 | 0.670728 | 0.106411 |
|  |  | 1 | 3.728585 | 1.191935 | -1.778328 |
|  |  | 1 | 3.662216 | -0.541395 | -1.393665 |
|  |  | 1 | 5.658809 | 0.573448 | -0.332135 |
|  |  | 1 | 4.573189 | 1.665740 | 0.548460 |
|  |  | 1 | 4.564898 | -0.072326 | 0.900709 |
|  |  |  |  |  |  |
|  |  |  |  |  |  |
| **5** | -1017.16514 | 7 | 2.218749 | -0.177823 | -0.564962 |
|  |  | 6 | 1.109449 | 0.532597 | -0.241451 |
|  |  | 7 | 0.045827 | -0.142129 | 0.096768 |
|  |  | 15 | -1.415545 | 0.401830 | 0.555670 |
|  |  | 8 | -1.742504 | 1.840364 | 0.708968 |
|  |  | 9 | -1.622732 | -0.356950 | 1.959395 |
|  |  | 8 | -2.409561 | -0.420904 | -0.399063 |
|  |  | 6 | 2.171395 | -1.651965 | -0.503427 |
|  |  | 6 | 2.287416 | -2.226669 | 0.910236 |
|  |  | 1 | 2.987370 | -2.018028 | -1.131046 |
|  |  | 1 | 1.229282 | -1.972678 | -0.950163 |
|  |  | 1 | 2.254438 | -3.318975 | 0.863108 |
|  |  | 1 | 3.225337 | -1.937081 | 1.389503 |
|  |  | 1 | 1.455981 | -1.888694 | 1.528874 |
|  |  | 6 | 3.471437 | 0.436444 | -1.020923 |
|  |  | 6 | 4.565150 | 0.506231 | 0.049302 |
|  |  | 1 | 3.261655 | 1.433756 | -1.401579 |
|  |  | 1 | 3.827845 | -0.148511 | -1.874309 |
|  |  | 1 | 5.455571 | 0.989988 | -0.362178 |
|  |  | 1 | 4.233664 | 1.082121 | 0.916814 |
|  |  | 1 | 4.854262 | -0.488751 | 0.393567 |
|  |  | 6 | 1.188421 | 2.044782 | -0.310465 |
|  |  | 1 | 0.287553 | 2.480237 | 0.115112 |
|  |  | 1 | 2.059233 | 2.421460 | 0.229475 |
|  |  | 1 | 1.268950 | 2.372271 | -1.351387 |
|  |  | 6 | -3.837075 | -0.156835 | -0.363492 |
|  |  | 6 | -4.467910 | -0.856363 | -1.549708 |
|  |  | 1 | -3.996881 | 0.923461 | -0.397177 |
|  |  | 1 | -4.235695 | -0.539784 | 0.580821 |
|  |  | 1 | -5.548873 | -0.689900 | -1.544191 |
|  |  | 1 | -4.063430 | -0.469015 | -2.487426 |
|  |  | 1 | -4.283699 | -1.932128 | -1.509730 |
|  |  |  |  |  |  |
|  |  |  |  |  |  |
| **6** | -1017.16531 | 7 | -2.319401 | 0.057957 | 0.349269 |
|  |  | 6 | -1.201481 | -0.645535 | 0.040469 |
|  |  | 7 | -0.059926 | -0.158793 | 0.441646 |
|  |  | 15 | 1.440534 | -0.742848 | 0.220947 |
|  |  | 8 | 1.748729 | -1.884446 | -0.674330 |
|  |  | 9 | 1.905851 | -1.081452 | 1.724287 |
|  |  | 8 | 2.279523 | 0.592506 | -0.076945 |
|  |  | 6 | -2.187366 | 1.324926 | 1.095106 |
|  |  | 6 | -1.742143 | 2.514143 | 0.240788 |
|  |  | 1 | -1.466164 | 1.163932 | 1.897581 |
|  |  | 1 | -3.161362 | 1.524965 | 1.547759 |
|  |  | 1 | -1.682281 | 3.409566 | 0.866219 |
|  |  | 1 | -0.754845 | 2.330167 | -0.183122 |
|  |  | 1 | -2.445425 | 2.714839 | -0.570673 |
|  |  | 6 | -3.677322 | -0.390469 | 0.019019 |
|  |  | 6 | -4.295397 | 0.312610 | -1.193493 |
|  |  | 1 | -4.299922 | -0.218549 | 0.902304 |
|  |  | 1 | -3.667948 | -1.466392 | -0.141828 |
|  |  | 1 | -5.295438 | -0.087320 | -1.383621 |
|  |  | 1 | -4.391169 | 1.387866 | -1.029325 |
|  |  | 1 | -3.689946 | 0.158966 | -2.090185 |
|  |  | 6 | -1.365606 | -1.941551 | -0.728093 |
|  |  | 1 | -0.391816 | -2.305573 | -1.047361 |
|  |  | 1 | -1.823749 | -2.703155 | -0.089773 |
|  |  | 1 | -2.003718 | -1.807307 | -1.603455 |
|  |  | 6 | 3.681842 | 0.503342 | -0.443170 |
|  |  | 6 | 4.118976 | 1.865526 | -0.940925 |
|  |  | 1 | 3.797920 | -0.265677 | -1.210457 |
|  |  | 1 | 4.251720 | 0.203310 | 0.441478 |
|  |  | 1 | 5.178116 | 1.836560 | -1.212253 |
|  |  | 1 | 3.544439 | 2.158253 | -1.822494 |
|  |  | 1 | 3.981183 | 2.624756 | -0.167988 |
|  |  |  |  |  |  |
|  |  |  |  |  |  |
| **7** | -1017.16489 | 7 | 2.116714 | 0.387801 | 0.053815 |
|  |  | 6 | 1.118145 | -0.508889 | 0.255920 |
|  |  | 7 | -0.088972 | -0.166655 | -0.103839 |
|  |  | 15 | -1.462094 | -1.057899 | -0.058946 |
|  |  | 8 | -1.549058 | -2.343578 | 0.660824 |
|  |  | 9 | -1.771524 | -1.235004 | -1.631770 |
|  |  | 8 | -2.623310 | -0.045498 | 0.369502 |
|  |  | 6 | 1.824943 | 1.682134 | -0.581942 |
|  |  | 6 | 1.417515 | 2.760353 | 0.423905 |
|  |  | 1 | 1.030552 | 1.524799 | -1.309778 |
|  |  | 1 | 2.723280 | 1.987276 | -1.124500 |
|  |  | 1 | 1.223271 | 3.703466 | -0.094638 |
|  |  | 1 | 0.509997 | 2.464787 | 0.952809 |
|  |  | 1 | 2.204631 | 2.940840 | 1.161539 |
|  |  | 6 | 3.512909 | 0.158873 | 0.444546 |
|  |  | 6 | 4.391404 | -0.363106 | -0.696746 |
|  |  | 1 | 3.546952 | -0.525775 | 1.290252 |
|  |  | 1 | 3.906358 | 1.112128 | 0.809084 |
|  |  | 1 | 5.424211 | -0.472874 | -0.354541 |
|  |  | 1 | 4.044693 | -1.336507 | -1.051143 |
|  |  | 1 | 4.391047 | 0.323688 | -1.546667 |
|  |  | 6 | 1.488224 | -1.827873 | 0.904024 |
|  |  | 1 | 0.633893 | -2.499807 | 0.894556 |
|  |  | 1 | 2.330013 | -2.299395 | 0.393717 |
|  |  | 1 | 1.775357 | -1.664533 | 1.947109 |
|  |  | 6 | -2.871216 | 1.220497 | -0.288917 |
|  |  | 6 | -4.003836 | 1.909936 | 0.444200 |
|  |  | 1 | -3.132797 | 1.023973 | -1.332366 |
|  |  | 1 | -1.953595 | 1.813658 | -0.264130 |
|  |  | 1 | -4.223580 | 2.869270 | -0.033156 |
|  |  | 1 | -4.907322 | 1.297195 | 0.422896 |
|  |  | 1 | -3.735606 | 2.094366 | 1.486706 |
|  |  |  |  |  |  |
|  |  |  |  |  |  |
| **8** | -1017.16471 | 7 | 2.344360 | 0.065345 | -0.380554 |
|  |  | 6 | 1.193654 | -0.610705 | -0.136482 |
|  |  | 7 | 0.072975 | 0.019866 | -0.350712 |
|  |  | 15 | -1.454610 | -0.498183 | -0.148998 |
|  |  | 8 | -1.805826 | -1.757832 | 0.552027 |
|  |  | 9 | -1.995414 | -0.539645 | -1.663609 |
|  |  | 8 | -2.186576 | 0.808050 | 0.425533 |
|  |  | 6 | 2.270448 | 1.461699 | -0.852843 |
|  |  | 6 | 1.958807 | 2.480574 | 0.245650 |
|  |  | 1 | 1.500618 | 1.512206 | -1.624289 |
|  |  | 1 | 3.233188 | 1.686278 | -1.317989 |
|  |  | 1 | 1.938525 | 3.485919 | -0.184898 |
|  |  | 1 | 0.981337 | 2.279655 | 0.684306 |
|  |  | 1 | 2.712779 | 2.465887 | 1.036083 |
|  |  | 6 | 3.679658 | -0.530368 | -0.256277 |
|  |  | 6 | 4.446378 | -0.098187 | 0.997364 |
|  |  | 1 | 4.246126 | -0.249787 | -1.149909 |
|  |  | 1 | 3.590447 | -1.614214 | -0.280095 |
|  |  | 1 | 5.421278 | -0.593157 | 1.024113 |
|  |  | 1 | 4.618944 | 0.979995 | 1.011700 |
|  |  | 1 | 3.901554 | -0.366491 | 1.905885 |
|  |  | 6 | 1.297590 | -2.042469 | 0.350248 |
|  |  | 1 | 0.318507 | -2.394484 | 0.666882 |
|  |  | 1 | 1.650698 | -2.691050 | -0.457282 |
|  |  | 1 | 1.996640 | -2.131323 | 1.183933 |
|  |  | 6 | -3.480720 | 0.714709 | 1.079780 |
|  |  | 6 | -4.623468 | 0.810768 | 0.083597 |
|  |  | 1 | -3.493398 | 1.554582 | 1.775660 |
|  |  | 1 | -3.521375 | -0.217106 | 1.648063 |
|  |  | 1 | -5.576099 | 0.821191 | 0.622082 |
|  |  | 1 | -4.549332 | 1.728027 | -0.504879 |
|  |  | 1 | -4.628775 | -0.040909 | -0.599751 |
|  |  |  |  |  |  |
|  |  |  |  |  |  |
| **9** | -1017.16432 | 7 | -2.219206 | 0.253654 | -0.061537 |
|  |  | 6 | -1.126132 | -0.539609 | 0.058888 |
|  |  | 7 | 0.021729 | 0.043725 | 0.276220 |
|  |  | 15 | 1.492780 | -0.644358 | 0.469690 |
|  |  | 8 | 1.783398 | -1.993157 | -0.074017 |
|  |  | 9 | 1.711303 | -0.567185 | 2.050678 |
|  |  | 8 | 2.549149 | 0.487630 | 0.051967 |
|  |  | 6 | -2.079010 | 1.715434 | 0.051425 |
|  |  | 6 | -2.160468 | 2.209038 | 1.497043 |
|  |  | 1 | -2.872112 | 2.163230 | -0.552951 |
|  |  | 1 | -1.121256 | 1.994510 | -0.384690 |
|  |  | 1 | -2.081442 | 3.299369 | 1.525034 |
|  |  | 1 | -3.109164 | 1.929336 | 1.964239 |
|  |  | 1 | -1.342962 | 1.791331 | 2.086339 |
|  |  | 6 | -3.580629 | -0.255000 | -0.263325 |
|  |  | 6 | -4.015500 | -0.276606 | -1.731943 |
|  |  | 1 | -4.252309 | 0.387599 | 0.312692 |
|  |  | 1 | -3.666791 | -1.249503 | 0.172124 |
|  |  | 1 | -5.048855 | -0.625239 | -1.812982 |
|  |  | 1 | -3.961416 | 0.719725 | -2.177614 |
|  |  | 1 | -3.383323 | -0.943620 | -2.322810 |
|  |  | 6 | -1.329102 | -2.037381 | -0.050196 |
|  |  | 1 | -0.366824 | -2.528359 | -0.176969 |
|  |  | 1 | -1.792533 | -2.416217 | 0.866447 |
|  |  | 1 | -1.976944 | -2.299649 | -0.887498 |
|  |  | 6 | 3.106311 | 0.516795 | -1.284436 |
|  |  | 6 | 4.264581 | 1.493231 | -1.283635 |
|  |  | 1 | 2.324171 | 0.834187 | -1.982468 |
|  |  | 1 | 3.428499 | -0.491503 | -1.555103 |
|  |  | 1 | 4.703011 | 1.547234 | -2.284254 |
|  |  | 1 | 3.930581 | 2.492894 | -0.997389 |
|  |  | 1 | 5.037888 | 1.171834 | -0.582914 |
|  |  |  |  |  |  |
|  |  |  |  |  |  |
| **10** | -1017.16459 | 7 | 2.291746 | 0.069419 | 0.503882 |
|  |  | 6 | 1.147123 | -0.574306 | 0.162939 |
|  |  | 7 | 0.065313 | 0.145518 | 0.059127 |
|  |  | 15 | -1.444296 | -0.309690 | -0.336909 |
|  |  | 8 | -1.814729 | -1.701376 | -0.694494 |
|  |  | 9 | -1.759581 | 0.674614 | -1.569048 |
|  |  | 8 | -2.327280 | 0.337497 | 0.835309 |
|  |  | 6 | 2.255973 | 1.527941 | 0.728319 |
|  |  | 6 | 2.237633 | 2.362062 | -0.554730 |
|  |  | 1 | 3.135028 | 1.773653 | 1.328992 |
|  |  | 1 | 1.367077 | 1.751310 | 1.320101 |
|  |  | 1 | 2.223797 | 3.425164 | -0.297603 |
|  |  | 1 | 3.119275 | 2.174700 | -1.171904 |
|  |  | 1 | 1.344093 | 2.143019 | -1.139375 |
|  |  | 6 | 3.575238 | -0.609007 | 0.716036 |
|  |  | 6 | 4.581439 | -0.418527 | -0.422953 |
|  |  | 1 | 3.395541 | -1.669673 | 0.875848 |
|  |  | 1 | 3.997461 | -0.224539 | 1.650082 |
|  |  | 1 | 5.500177 | -0.968887 | -0.201268 |
|  |  | 1 | 4.180393 | -0.789012 | -1.369571 |
|  |  | 1 | 4.845770 | 0.632645 | -0.554901 |
|  |  | 6 | 1.211109 | -2.072520 | -0.059448 |
|  |  | 1 | 0.259756 | -2.426510 | -0.449192 |
|  |  | 1 | 2.006430 | -2.335043 | -0.760077 |
|  |  | 1 | 1.408944 | -2.589042 | 0.884501 |
|  |  | 6 | -3.704322 | -0.073812 | 1.052392 |
|  |  | 6 | -4.672806 | 0.741738 | 0.213223 |
|  |  | 1 | -3.871385 | 0.090217 | 2.117742 |
|  |  | 1 | -3.790787 | -1.141906 | 0.840814 |
|  |  | 1 | -5.699834 | 0.459461 | 0.464461 |
|  |  | 1 | -4.550589 | 1.809627 | 0.407654 |
|  |  | 1 | -4.522132 | 0.563697 | -0.853497 |
|  |  |  |  |  |  |
|  |  |  |  |  |  |
| **11** | -1017.16421 | 7 | -2.150527 | 0.338760 | 0.032714 |
|  |  | 6 | -1.130625 | -0.550677 | 0.128833 |
|  |  | 7 | 0.085850 | -0.100842 | -0.019080 |
|  |  | 15 | 1.489045 | -0.945959 | 0.017775 |
|  |  | 8 | 1.539286 | -2.402045 | -0.218062 |
|  |  | 9 | 2.055613 | -0.562989 | 1.482489 |
|  |  | 8 | 2.494780 | -0.144882 | -0.927589 |
|  |  | 6 | -3.562808 | -0.013943 | 0.221407 |
|  |  | 6 | -4.302265 | -0.307420 | -1.087534 |
|  |  | 1 | -4.037860 | 0.826586 | 0.735001 |
|  |  | 1 | -3.638338 | -0.863176 | 0.898984 |
|  |  | 1 | -5.354785 | -0.523318 | -0.883930 |
|  |  | 1 | -4.260160 | 0.546331 | -1.768372 |
|  |  | 1 | -3.870746 | -1.168898 | -1.602298 |
|  |  | 6 | -1.864466 | 1.753854 | -0.251330 |
|  |  | 6 | -1.572126 | 2.567719 | 1.010699 |
|  |  | 1 | -2.732824 | 2.160934 | -0.775480 |
|  |  | 1 | -1.014061 | 1.793010 | -0.930658 |
|  |  | 1 | -1.382873 | 3.612364 | 0.747845 |
|  |  | 1 | -2.415694 | 2.545711 | 1.706456 |
|  |  | 1 | -0.691093 | 2.175370 | 1.520636 |
|  |  | 6 | -1.484902 | -1.994337 | 0.422239 |
|  |  | 1 | -1.764557 | -2.099692 | 1.475457 |
|  |  | 1 | -2.322481 | -2.339188 | -0.184878 |
|  |  | 1 | -0.623014 | -2.629656 | 0.231140 |
|  |  | 6 | 2.748140 | 1.280551 | -0.843810 |
|  |  | 6 | 4.087536 | 1.544334 | -0.179553 |
|  |  | 1 | 1.927015 | 1.765880 | -0.311520 |
|  |  | 1 | 2.743044 | 1.633376 | -1.876898 |
|  |  | 1 | 4.292794 | 2.619414 | -0.178296 |
|  |  | 1 | 4.087679 | 1.190162 | 0.852938 |
|  |  | 1 | 4.891051 | 1.040582 | -0.721096 |
|  |  |  |  |  |  |
|  |  |  |  |  |  |
| **12** | -1017.16353 | 7 | -2.250594 | 0.144946 | -0.087459 |
|  |  | 6 | -0.962361 | 0.213526 | 0.323087 |
|  |  | 7 | -0.125048 | -0.659792 | -0.163644 |
|  |  | 15 | 1.456997 | -0.872319 | 0.129151 |
|  |  | 8 | 2.103601 | -2.053888 | -0.466643 |
|  |  | 9 | 1.592335 | -0.906506 | 1.747817 |
|  |  | 8 | 2.177729 | 0.537016 | -0.200098 |
|  |  | 6 | -2.648253 | -0.880477 | -1.069170 |
|  |  | 6 | -3.012850 | -2.216381 | -0.420186 |
|  |  | 1 | -1.820078 | -1.016471 | -1.762771 |
|  |  | 1 | -3.498076 | -0.478249 | -1.626597 |
|  |  | 1 | -3.331852 | -2.927221 | -1.187293 |
|  |  | 1 | -2.147689 | -2.636208 | 0.094442 |
|  |  | 1 | -3.831685 | -2.106389 | 0.296955 |
|  |  | 6 | -3.323761 | 1.002165 | 0.429506 |
|  |  | 6 | -3.610339 | 2.223460 | -0.449236 |
|  |  | 1 | -3.086618 | 1.310657 | 1.446938 |
|  |  | 1 | -4.220657 | 0.381209 | 0.504018 |
|  |  | 1 | -4.447972 | 2.793748 | -0.038252 |
|  |  | 1 | -2.742426 | 2.884562 | -0.506329 |
|  |  | 1 | -3.872842 | 1.926160 | -1.467275 |
|  |  | 6 | -0.597063 | 1.285018 | 1.330841 |
|  |  | 1 | 0.475071 | 1.459245 | 1.310626 |
|  |  | 1 | -1.102842 | 2.229503 | 1.131060 |
|  |  | 1 | -0.868787 | 0.955733 | 2.338406 |
|  |  | 6 | 3.619321 | 0.602527 | -0.378336 |
|  |  | 6 | 3.956026 | 1.950983 | -0.980412 |
|  |  | 1 | 3.927148 | -0.220694 | -1.026180 |
|  |  | 1 | 4.094654 | 0.472410 | 0.598590 |
|  |  | 1 | 5.037852 | 2.033059 | -1.118731 |
|  |  | 1 | 3.473990 | 2.072021 | -1.953034 |
|  |  | 1 | 3.631375 | 2.764239 | -0.327095 |
|  |  |  |  |  |  |
|  |  |  |  |  |  |
| **13** | -1017.16334 | 7 | -2.245541 | 0.032247 | 0.197760 |
|  |  | 6 | -0.974785 | -0.362430 | -0.054683 |
|  |  | 7 | -0.118944 | 0.550707 | -0.419226 |
|  |  | 15 | 1.453016 | 0.432177 | -0.802813 |
|  |  | 8 | 2.124274 | 1.646880 | -1.295818 |
|  |  | 9 | 1.547965 | -0.753461 | -1.910972 |
|  |  | 8 | 2.167377 | -0.281055 | 0.458882 |
|  |  | 6 | -2.616428 | 1.448559 | 0.028685 |
|  |  | 6 | -2.362198 | 2.284570 | 1.283842 |
|  |  | 1 | -3.675228 | 1.473633 | -0.241433 |
|  |  | 1 | -2.042463 | 1.845548 | -0.806961 |
|  |  | 1 | -2.684259 | 3.315736 | 1.115094 |
|  |  | 1 | -2.914106 | 1.897329 | 2.145415 |
|  |  | 1 | -1.298113 | 2.294951 | 1.523551 |
|  |  | 6 | -3.305128 | -0.866154 | 0.670364 |
|  |  | 6 | -4.201956 | -1.398916 | -0.451472 |
|  |  | 1 | -3.906112 | -0.302701 | 1.389560 |
|  |  | 1 | -2.863286 | -1.690974 | 1.227887 |
|  |  | 1 | -4.998127 | -2.020567 | -0.032571 |
|  |  | 1 | -4.669611 | -0.582382 | -1.006542 |
|  |  | 1 | -3.633767 | -2.004548 | -1.161375 |
|  |  | 6 | -0.648781 | -1.831481 | 0.126725 |
|  |  | 1 | 0.307259 | -2.064403 | -0.331688 |
|  |  | 1 | -0.576416 | -2.067791 | 1.192461 |
|  |  | 1 | -1.410832 | -2.475117 | -0.314247 |
|  |  | 6 | 3.612449 | -0.233880 | 0.610446 |
|  |  | 6 | 3.950151 | -0.680329 | 2.018009 |
|  |  | 1 | 3.950297 | 0.785849 | 0.414800 |
|  |  | 1 | 4.060340 | -0.897447 | -0.135439 |
|  |  | 1 | 5.034536 | -0.665113 | 2.159653 |
|  |  | 1 | 3.496765 | -0.013680 | 2.754897 |
|  |  | 1 | 3.594425 | -1.696746 | 2.202144 |
|  |  |  |  |  |  |
|  |  |  |  |  |  |
| **14** | -1017.16367 | 7 | 2.131098 | 0.203229 | -0.426279 |
|  |  | 6 | 1.123451 | -0.650712 | -0.109653 |
|  |  | 7 | -0.100923 | -0.212650 | -0.222858 |
|  |  | 15 | -1.498218 | -1.021034 | 0.060475 |
|  |  | 8 | -1.551061 | -2.225089 | 0.912578 |
|  |  | 9 | -2.009083 | -1.328639 | -1.439517 |
|  |  | 8 | -2.549808 | 0.090854 | 0.520115 |
|  |  | 6 | 1.804388 | 1.570181 | -0.870526 |
|  |  | 6 | 1.475950 | 2.540880 | 0.266838 |
|  |  | 1 | 0.954512 | 1.502154 | -1.550705 |
|  |  | 1 | 2.663743 | 1.932170 | -1.439724 |
|  |  | 1 | 1.225299 | 3.521300 | -0.148797 |
|  |  | 1 | 0.619833 | 2.183994 | 0.840238 |
|  |  | 1 | 2.320822 | 2.670319 | 0.946565 |
|  |  | 6 | 3.550004 | -0.173622 | -0.390130 |
|  |  | 6 | 4.295656 | 0.287046 | 0.866295 |
|  |  | 1 | 4.021316 | 0.258033 | -1.277956 |
|  |  | 1 | 3.634346 | -1.253274 | -0.496641 |
|  |  | 1 | 5.333622 | -0.055443 | 0.827848 |
|  |  | 1 | 4.305995 | 1.375498 | 0.951521 |
|  |  | 1 | 3.835283 | -0.119090 | 1.770281 |
|  |  | 6 | 1.497850 | -2.049360 | 0.338090 |
|  |  | 1 | 0.624643 | -2.546844 | 0.753399 |
|  |  | 1 | 1.856360 | -2.633627 | -0.515213 |
|  |  | 1 | 2.287779 | -2.031739 | 1.090512 |
|  |  | 6 | -2.819912 | 1.301095 | -0.228351 |
|  |  | 6 | -3.804733 | 2.129660 | 0.571053 |
|  |  | 1 | -3.231846 | 1.021950 | -1.202126 |
|  |  | 1 | -1.878216 | 1.832797 | -0.386036 |
|  |  | 1 | -4.039801 | 3.050040 | 0.028726 |
|  |  | 1 | -4.732127 | 1.576700 | 0.733424 |
|  |  | 1 | -3.386743 | 2.397131 | 1.543927 |
|  |  |  |  |  |  |
|  |  |  |  |  |  |
| **15** | -1017.16311 | 7 | 2.307956 | -0.085039 | -0.041375 |
|  |  | 6 | 0.998705 | -0.300732 | 0.229855 |
|  |  | 7 | 0.144528 | 0.609240 | -0.146439 |
|  |  | 15 | -1.467484 | 0.679635 | 0.031384 |
|  |  | 8 | -2.146920 | 1.899639 | -0.438717 |
|  |  | 9 | -1.719013 | 0.461047 | 1.619560 |
|  |  | 8 | -2.068342 | -0.697365 | -0.564490 |
|  |  | 6 | 2.711635 | 1.138755 | -0.756738 |
|  |  | 6 | 2.944934 | 2.325935 | 0.178830 |
|  |  | 1 | 1.929072 | 1.377968 | -1.474819 |
|  |  | 1 | 3.623050 | 0.900719 | -1.311169 |
|  |  | 1 | 3.273901 | 3.194627 | -0.397963 |
|  |  | 1 | 2.020750 | 2.589673 | 0.694665 |
|  |  | 1 | 3.715114 | 2.106803 | 0.924209 |
|  |  | 6 | 3.392205 | -0.978339 | 0.382987 |
|  |  | 6 | 3.831087 | -1.966693 | -0.701730 |
|  |  | 1 | 3.095744 | -1.510443 | 1.286129 |
|  |  | 1 | 4.235692 | -0.344143 | 0.669604 |
|  |  | 1 | 4.669853 | -2.570229 | -0.343855 |
|  |  | 1 | 3.017301 | -2.641907 | -0.976514 |
|  |  | 1 | 4.153682 | -1.445261 | -1.606001 |
|  |  | 6 | 0.630601 | -1.573119 | 0.966444 |
|  |  | 1 | -0.427068 | -1.783575 | 0.835197 |
|  |  | 1 | 1.198445 | -2.433140 | 0.611210 |
|  |  | 1 | 0.824626 | -1.452711 | 2.036673 |
|  |  | 6 | -3.433058 | -0.772666 | -1.062936 |
|  |  | 6 | -4.432549 | -1.024294 | 0.052523 |
|  |  | 1 | -3.416945 | -1.599382 | -1.774453 |
|  |  | 1 | -3.658245 | 0.152464 | -1.597157 |
|  |  | 1 | -5.431917 | -1.149114 | -0.375540 |
|  |  | 1 | -4.180585 | -1.931444 | 0.607109 |
|  |  | 1 | -4.465740 | -0.185531 | 0.750824 |
|  |  |  |  |  |  |
|  |  |  |  |  |  |
| **16** | -1017.16297 | 7 | -2.306655 | 0.026917 | 0.127491 |
|  |  | 6 | -1.013554 | -0.368177 | 0.044878 |
|  |  | 7 | -0.140921 | 0.500483 | -0.382060 |
|  |  | 15 | 1.462280 | 0.371564 | -0.599166 |
|  |  | 8 | 2.152430 | 1.505907 | -1.237868 |
|  |  | 9 | 1.672934 | -0.983529 | -1.471335 |
|  |  | 8 | 2.077859 | -0.081952 | 0.823155 |
|  |  | 6 | -3.392247 | -0.820095 | 0.634398 |
|  |  | 6 | -4.168262 | -1.546539 | -0.468686 |
|  |  | 1 | -4.068867 | -0.171298 | 1.197442 |
|  |  | 1 | -2.992972 | -1.534849 | 1.352931 |
|  |  | 1 | -4.988367 | -2.124141 | -0.033200 |
|  |  | 1 | -4.597141 | -0.839924 | -1.183223 |
|  |  | 1 | -3.523441 | -2.232965 | -1.022388 |
|  |  | 6 | -2.678325 | 1.393520 | -0.279614 |
|  |  | 6 | -2.546378 | 2.407200 | 0.857905 |
|  |  | 1 | -3.709052 | 1.353054 | -0.641058 |
|  |  | 1 | -2.037186 | 1.679739 | -1.111723 |
|  |  | 1 | -2.858783 | 3.396726 | 0.513349 |
|  |  | 1 | -3.171899 | 2.136438 | 1.713670 |
|  |  | 1 | -1.508727 | 2.472861 | 1.187953 |
|  |  | 6 | -0.682227 | -1.781940 | 0.479204 |
|  |  | 1 | -0.671256 | -1.839951 | 1.571764 |
|  |  | 1 | -1.406260 | -2.506809 | 0.106067 |
|  |  | 1 | 0.302927 | -2.063257 | 0.119948 |
|  |  | 6 | 3.450849 | 0.234477 | 1.184755 |
|  |  | 6 | 4.423835 | -0.817666 | 0.682450 |
|  |  | 1 | 3.442860 | 0.278506 | 2.274809 |
|  |  | 1 | 3.694596 | 1.222812 | 0.790099 |
|  |  | 1 | 5.431607 | -0.581723 | 1.037836 |
|  |  | 1 | 4.152534 | -1.809433 | 1.052102 |
|  |  | 1 | 4.447398 | -0.845605 | -0.408928 |
|  |  |  |  |  |  |
|  |  |  |  |  |  |
| **17** | -1017.16295 | 7 | 2.255635 | -0.054530 | -0.127706 |
|  |  | 6 | 0.998333 | -0.246722 | 0.336288 |
|  |  | 7 | 0.070167 | 0.573696 | -0.071255 |
|  |  | 15 | -1.509296 | 0.637116 | 0.296884 |
|  |  | 8 | -2.285007 | 1.771377 | -0.234776 |
|  |  | 9 | -1.569401 | 0.625755 | 1.919653 |
|  |  | 8 | -2.118374 | -0.822065 | -0.033505 |
|  |  | 6 | 2.522753 | 1.040528 | -1.077417 |
|  |  | 6 | 2.824812 | 2.369537 | -0.383613 |
|  |  | 1 | 1.649514 | 1.144568 | -1.719174 |
|  |  | 1 | 3.367382 | 0.728736 | -1.697287 |
|  |  | 1 | 3.050227 | 3.135842 | -1.130255 |
|  |  | 1 | 1.961035 | 2.700567 | 0.194280 |
|  |  | 1 | 3.686809 | 2.288696 | 0.285145 |
|  |  | 6 | 3.416803 | -0.845529 | 0.296810 |
|  |  | 6 | 3.752304 | -1.999893 | -0.652754 |
|  |  | 1 | 3.256206 | -1.217387 | 1.308091 |
|  |  | 1 | 4.265627 | -0.159158 | 0.358702 |
|  |  | 1 | 4.652745 | -2.517838 | -0.311068 |
|  |  | 1 | 2.937623 | -2.726414 | -0.699427 |
|  |  | 1 | 3.936970 | -1.637511 | -1.666828 |
|  |  | 6 | 0.770557 | -1.386615 | 1.308599 |
|  |  | 1 | -0.283804 | -1.647784 | 1.330620 |
|  |  | 1 | 1.340239 | -2.276361 | 1.041319 |
|  |  | 1 | 1.066141 | -1.078989 | 2.316320 |
|  |  | 6 | -3.551657 | -1.015612 | -0.205401 |
|  |  | 6 | -3.935894 | -0.936293 | -1.670886 |
|  |  | 1 | -4.093300 | -0.272301 | 0.383602 |
|  |  | 1 | -3.753259 | -2.004201 | 0.210708 |
|  |  | 1 | -5.004388 | -1.144739 | -1.783457 |
|  |  | 1 | -3.734698 | 0.061354 | -2.064627 |
|  |  | 1 | -3.378649 | -1.670673 | -2.257571 |
|  |  |  |  |  |  |
|  |  |  |  |  |  |
| **18** | -1017.16276 | 7 | -2.248255 | 0.185902 | 0.131176 |
|  |  | 6 | -1.013535 | -0.370575 | 0.122181 |
|  |  | 7 | -0.075395 | 0.248887 | -0.538135 |
|  |  | 15 | 1.485483 | -0.135342 | -0.760823 |
|  |  | 8 | 2.274573 | 0.722949 | -1.661543 |
|  |  | 9 | 1.486204 | -1.666759 | -1.305868 |
|  |  | 8 | 2.110770 | -0.344243 | 0.712560 |
|  |  | 6 | -2.491986 | 1.431954 | -0.616942 |
|  |  | 6 | -2.189399 | 2.687044 | 0.202831 |
|  |  | 1 | -3.539506 | 1.420338 | -0.928832 |
|  |  | 1 | -1.870704 | 1.410333 | -1.510819 |
|  |  | 1 | -2.415064 | 3.579645 | -0.386969 |
|  |  | 1 | -2.790537 | 2.727159 | 1.115969 |
|  |  | 1 | -1.133615 | 2.716618 | 0.475378 |
|  |  | 6 | -3.390885 | -0.366504 | 0.867901 |
|  |  | 6 | -4.299304 | -1.254280 | 0.011572 |
|  |  | 1 | -3.961299 | 0.481371 | 1.256944 |
|  |  | 1 | -3.033106 | -0.914477 | 1.738524 |
|  |  | 1 | -5.155173 | -1.595462 | 0.600533 |
|  |  | 1 | -4.682204 | -0.709754 | -0.854658 |
|  |  | 1 | -3.764380 | -2.133495 | -0.354997 |
|  |  | 6 | -0.817692 | -1.657227 | 0.899115 |
|  |  | 1 | 0.111255 | -2.135459 | 0.604333 |
|  |  | 1 | -0.756986 | -1.438669 | 1.969448 |
|  |  | 1 | -1.635485 | -2.360782 | 0.740069 |
|  |  | 6 | 3.550631 | -0.308339 | 0.927129 |
|  |  | 6 | 3.989431 | 1.064139 | 1.401725 |
|  |  | 1 | 4.065058 | -0.586859 | 0.004913 |
|  |  | 1 | 3.743365 | -1.074977 | 1.679686 |
|  |  | 1 | 5.061829 | 1.053501 | 1.619932 |
|  |  | 1 | 3.801564 | 1.813618 | 0.630936 |
|  |  | 1 | 3.456153 | 1.347877 | 2.312273 |
|  |  |  |  |  |  |
|  |  |  |  |  |  |
| **19** | -1017.16284 | 7 | -2.125423 | 0.283432 | -0.455215 |
|  |  | 6 | -1.106784 | -0.597065 | -0.276495 |
|  |  | 7 | 0.083293 | -0.105879 | -0.066659 |
|  |  | 15 | 1.486263 | -0.890124 | 0.244687 |
|  |  | 8 | 1.587737 | -2.362425 | 0.273653 |
|  |  | 9 | 1.873537 | -0.241098 | 1.669374 |
|  |  | 8 | 2.585350 | -0.225223 | -0.705111 |
|  |  | 6 | -1.851767 | 1.729585 | -0.370698 |
|  |  | 6 | -1.703509 | 2.259324 | 1.057851 |
|  |  | 1 | -2.676442 | 2.235653 | -0.878133 |
|  |  | 1 | -0.938032 | 1.930450 | -0.932517 |
|  |  | 1 | -1.508595 | 3.335457 | 1.030703 |
|  |  | 1 | -2.610021 | 2.095712 | 1.644991 |
|  |  | 1 | -0.867754 | 1.773865 | 1.562152 |
|  |  | 6 | -3.498027 | -0.116511 | -0.787264 |
|  |  | 6 | -4.483210 | -0.003969 | 0.379955 |
|  |  | 1 | -3.490191 | -1.135224 | -1.167542 |
|  |  | 1 | -3.832093 | 0.517768 | -1.614792 |
|  |  | 1 | -5.476643 | -0.329793 | 0.059078 |
|  |  | 1 | -4.173008 | -0.629628 | 1.220457 |
|  |  | 1 | -4.568527 | 1.024750 | 0.736215 |
|  |  | 6 | -1.428211 | -2.076261 | -0.350372 |
|  |  | 1 | -2.268708 | -2.331120 | 0.298322 |
|  |  | 1 | -1.694661 | -2.355569 | -1.374081 |
|  |  | 1 | -0.557309 | -2.657275 | -0.057106 |
|  |  | 6 | 2.818963 | 1.202262 | -0.815715 |
|  |  | 6 | 4.235608 | 1.518913 | -0.375335 |
|  |  | 1 | 2.081226 | 1.744934 | -0.221230 |
|  |  | 1 | 2.663549 | 1.453140 | -1.867434 |
|  |  | 1 | 4.437253 | 2.585696 | -0.513175 |
|  |  | 1 | 4.378141 | 1.272140 | 0.678633 |
|  |  | 1 | 4.957866 | 0.951436 | -0.965993 |
|  |  |  |  |  |  |
|  |  |  |  |  |  |
| **20** | -1017.16270 | 7 | -2.141711 | 0.212477 | 0.445175 |
|  |  | 6 | -1.118439 | -0.635644 | 0.164427 |
|  |  | 7 | 0.089931 | -0.143017 | 0.151602 |
|  |  | 15 | 1.503669 | -0.918755 | -0.141000 |
|  |  | 8 | 1.559217 | -2.235252 | -0.806208 |
|  |  | 9 | 2.148098 | -0.971145 | 1.338748 |
|  |  | 8 | 2.443275 | 0.156934 | -0.854244 |
|  |  | 6 | -3.541460 | -0.215922 | 0.557770 |
|  |  | 6 | -4.402081 | 0.112658 | -0.665948 |
|  |  | 1 | -3.959068 | 0.271105 | 1.444339 |
|  |  | 1 | -3.572354 | -1.285014 | 0.756310 |
|  |  | 1 | -5.421019 | -0.253574 | -0.511843 |
|  |  | 1 | -4.457288 | 1.189032 | -0.841394 |
|  |  | 1 | -4.002596 | -0.357858 | -1.567747 |
|  |  | 6 | -1.850734 | 1.634391 | 0.702864 |
|  |  | 6 | -1.604133 | 2.464967 | -0.559082 |
|  |  | 1 | -0.974463 | 1.682226 | 1.351086 |
|  |  | 1 | -2.701936 | 2.035332 | 1.257934 |
|  |  | 1 | -1.389638 | 3.500788 | -0.280050 |
|  |  | 1 | -0.748829 | 2.076110 | -1.111959 |
|  |  | 1 | -2.474651 | 2.467212 | -1.218875 |
|  |  | 6 | -1.458287 | -2.088255 | -0.102695 |
|  |  | 1 | -1.751870 | -2.583979 | 0.827835 |
|  |  | 1 | -2.284191 | -2.181454 | -0.809786 |
|  |  | 1 | -0.587637 | -2.600649 | -0.504990 |
|  |  | 6 | 2.701381 | 1.491595 | -0.348109 |
|  |  | 6 | 4.093159 | 1.569751 | 0.252609 |
|  |  | 1 | 1.929767 | 1.763305 | 0.375445 |
|  |  | 1 | 2.606719 | 2.150472 | -1.213462 |
|  |  | 1 | 4.299212 | 2.595587 | 0.573711 |
|  |  | 1 | 4.181825 | 0.911133 | 1.118580 |
|  |  | 1 | 4.846194 | 1.280933 | -0.483708 |
|  |  |  |  |  |  |
|  |  |  |  |  |  |
| **21** | -1017.16214 | 7 | 2.283880 | -0.097999 | -0.257862 |
|  |  | 6 | 0.978009 | 0.155807 | -0.517450 |
|  |  | 7 | 0.108677 | -0.748845 | -0.163901 |
|  |  | 15 | -1.500147 | -0.805577 | -0.371191 |
|  |  | 8 | -2.189309 | -2.046642 | 0.021042 |
|  |  | 9 | -1.743465 | -0.494398 | -1.946272 |
|  |  | 8 | -2.092379 | 0.542625 | 0.295953 |
|  |  | 6 | 3.382895 | 0.782167 | -0.671923 |
|  |  | 6 | 3.972535 | 1.627845 | 0.460692 |
|  |  | 1 | 3.040625 | 1.427074 | -1.478991 |
|  |  | 1 | 4.162734 | 0.145997 | -1.101353 |
|  |  | 1 | 4.776454 | 2.259402 | 0.072136 |
|  |  | 1 | 3.214308 | 2.275933 | 0.907389 |
|  |  | 1 | 4.391401 | 1.002577 | 1.251782 |
|  |  | 6 | 2.646465 | -1.362323 | 0.414484 |
|  |  | 6 | 2.416754 | -1.356714 | 1.927111 |
|  |  | 1 | 3.699641 | -1.546399 | 0.190073 |
|  |  | 1 | 2.056355 | -2.159398 | -0.039619 |
|  |  | 1 | 2.713252 | -2.323703 | 2.343139 |
|  |  | 1 | 3.002997 | -0.580065 | 2.423942 |
|  |  | 1 | 1.361357 | -1.201949 | 2.151142 |
|  |  | 6 | 0.629570 | 1.460575 | -1.206299 |
|  |  | 1 | -0.433848 | 1.658343 | -1.109551 |
|  |  | 1 | 1.173796 | 2.302291 | -0.775945 |
|  |  | 1 | 0.872511 | 1.403644 | -2.271483 |
|  |  | 6 | -3.511812 | 0.656793 | 0.590861 |
|  |  | 6 | -3.706091 | 1.848026 | 1.506037 |
|  |  | 1 | -3.846123 | -0.272573 | 1.056345 |
|  |  | 1 | -4.050732 | 0.786873 | -0.352463 |
|  |  | 1 | -4.768333 | 1.964232 | 1.739119 |
|  |  | 1 | -3.160742 | 1.709835 | 2.442208 |
|  |  | 1 | -3.356619 | 2.768102 | 1.031756 |
|  |  |  |  |  |  |
|  |  |  |  |  |  |
| **22** | -1017.16213 | 7 | 2.159347 | -0.329003 | -0.493064 |
|  |  | 6 | 0.920096 | -0.414162 | 0.049520 |
|  |  | 7 | 0.107061 | 0.584238 | -0.151542 |
|  |  | 15 | -1.430688 | 0.810116 | 0.315561 |
|  |  | 8 | -2.042571 | 2.116726 | 0.019707 |
|  |  | 9 | -1.444533 | 0.530007 | 1.916528 |
|  |  | 8 | -2.256908 | -0.470359 | -0.219997 |
|  |  | 6 | 2.523702 | 0.879365 | -1.260482 |
|  |  | 6 | 2.884416 | 2.089313 | -0.395852 |
|  |  | 1 | 1.677625 | 1.131331 | -1.901028 |
|  |  | 1 | 3.365284 | 0.602384 | -1.899745 |
|  |  | 1 | 3.136870 | 2.934679 | -1.042179 |
|  |  | 1 | 2.036765 | 2.380196 | 0.224460 |
|  |  | 1 | 3.743737 | 1.887437 | 0.247770 |
|  |  | 6 | 3.165106 | -1.393058 | -0.393176 |
|  |  | 6 | 4.246203 | -1.141368 | 0.662417 |
|  |  | 1 | 3.628696 | -1.493716 | -1.379077 |
|  |  | 1 | 2.665457 | -2.339328 | -0.194626 |
|  |  | 1 | 4.942674 | -1.984222 | 0.687367 |
|  |  | 1 | 4.820353 | -0.239425 | 0.441519 |
|  |  | 1 | 3.809335 | -1.029147 | 1.657802 |
|  |  | 6 | 0.575317 | -1.665086 | 0.833573 |
|  |  | 1 | 0.462535 | -2.517402 | 0.157488 |
|  |  | 1 | 1.350519 | -1.908494 | 1.562328 |
|  |  | 1 | -0.362727 | -1.530482 | 1.362542 |
|  |  | 6 | -3.708083 | -0.432105 | -0.301215 |
|  |  | 6 | -4.160382 | -1.606496 | -1.144259 |
|  |  | 1 | -4.007504 | 0.522365 | -0.738915 |
|  |  | 1 | -4.112897 | -0.491308 | 0.713580 |
|  |  | 1 | -5.251635 | -1.609363 | -1.217463 |
|  |  | 1 | -3.747212 | -1.540445 | -2.153158 |
|  |  | 1 | -3.844031 | -2.552865 | -0.699430 |
|  |  |  |  |  |  |
|  |  |  |  |  |  |
| **23** | -1017.16174 | 7 | -2.318611 | -0.036901 | -0.331036 |
|  |  | 6 | -1.006383 | -0.376941 | -0.351923 |
|  |  | 7 | -0.134096 | 0.591125 | -0.355566 |
|  |  | 15 | 1.487581 | 0.547523 | -0.432093 |
|  |  | 8 | 2.191829 | 1.832260 | -0.588357 |
|  |  | 9 | 1.824561 | -0.443815 | -1.672394 |
|  |  | 8 | 1.985143 | -0.346808 | 0.817829 |
|  |  | 6 | -2.686413 | 1.393140 | -0.303897 |
|  |  | 6 | -2.575331 | 2.046058 | 1.075476 |
|  |  | 1 | -3.712047 | 1.460470 | -0.674115 |
|  |  | 1 | -2.035070 | 1.915102 | -1.006125 |
|  |  | 1 | -2.873295 | 3.096006 | 1.004865 |
|  |  | 1 | -3.222595 | 1.559353 | 1.808852 |
|  |  | 1 | -1.546263 | 2.010868 | 1.433018 |
|  |  | 6 | -3.412909 | -1.012436 | -0.404222 |
|  |  | 6 | -4.090519 | -1.298818 | 0.939018 |
|  |  | 1 | -3.039435 | -1.937587 | -0.838831 |
|  |  | 1 | -4.148926 | -0.619854 | -1.112201 |
|  |  | 1 | -4.884129 | -2.039273 | 0.804617 |
|  |  | 1 | -3.375988 | -1.691160 | 1.666854 |
|  |  | 1 | -4.540926 | -0.397602 | 1.359576 |
|  |  | 6 | -0.653913 | -1.851453 | -0.384446 |
|  |  | 1 | 0.394218 | -1.989165 | -0.135927 |
|  |  | 1 | -1.251524 | -2.426486 | 0.324183 |
|  |  | 1 | -0.823831 | -2.258780 | -1.385479 |
|  |  | 6 | 3.305434 | -0.163520 | 1.400621 |
|  |  | 6 | 4.359630 | -0.986856 | 0.681845 |
|  |  | 1 | 3.192422 | -0.482652 | 2.437692 |
|  |  | 1 | 3.548942 | 0.900467 | 1.380063 |
|  |  | 1 | 5.319645 | -0.877971 | 1.195742 |
|  |  | 1 | 4.091739 | -2.046136 | 0.675454 |
|  |  | 1 | 4.487884 | -0.652331 | -0.349689 |
|  |  |  |  |  |  |
|  |  |  |  |  |  |
| **24** | -1017.16170 | 7 | 2.248372 | -0.181176 | -0.483047 |
|  |  | 6 | 0.969147 | -0.423888 | -0.105009 |
|  |  | 7 | 0.132087 | 0.574193 | -0.140325 |
|  |  | 15 | -1.452609 | 0.637036 | 0.207870 |
|  |  | 8 | -2.107606 | 1.955336 | 0.144756 |
|  |  | 9 | -1.582592 | 0.009991 | 1.700614 |
|  |  | 8 | -2.162886 | -0.515715 | -0.671809 |
|  |  | 6 | 3.285669 | -1.217237 | -0.554093 |
|  |  | 6 | 4.255875 | -1.218399 | 0.631069 |
|  |  | 1 | 3.839325 | -1.057096 | -1.484022 |
|  |  | 1 | 2.811350 | -2.192330 | -0.647931 |
|  |  | 1 | 4.988495 | -2.021527 | 0.510761 |
|  |  | 1 | 4.801339 | -0.275363 | 0.702420 |
|  |  | 1 | 3.727938 | -1.375804 | 1.574879 |
|  |  | 6 | 2.626280 | 1.186068 | -0.894621 |
|  |  | 6 | 2.847688 | 2.155400 | 0.268359 |
|  |  | 1 | 1.833366 | 1.568023 | -1.539102 |
|  |  | 1 | 3.536074 | 1.093054 | -1.492189 |
|  |  | 1 | 3.124666 | 3.137350 | -0.125572 |
|  |  | 1 | 1.932012 | 2.270348 | 0.848138 |
|  |  | 1 | 3.648207 | 1.818920 | 0.931506 |
|  |  | 6 | 0.611411 | -1.836399 | 0.313810 |
|  |  | 1 | 0.572837 | -2.491451 | -0.561363 |
|  |  | 1 | 1.340822 | -2.245515 | 1.014682 |
|  |  | 1 | -0.365680 | -1.853284 | 0.786274 |
|  |  | 6 | -3.566545 | -0.424856 | -1.043203 |
|  |  | 6 | -4.475316 | -0.978505 | 0.040267 |
|  |  | 1 | -3.643618 | -1.008675 | -1.961498 |
|  |  | 1 | -3.800480 | 0.618457 | -1.264221 |
|  |  | 1 | -5.512158 | -0.960755 | -0.309260 |
|  |  | 1 | -4.211638 | -2.010966 | 0.281847 |
|  |  | 1 | -4.414926 | -0.380032 | 0.951630 |
|  |  |  |  |  |  |
|  |  |  |  |  |  |
| **25** | -1017.16150 | 7 | -2.285213 | -0.058396 | -0.271531 |
|  |  | 6 | -1.013905 | -0.523191 | -0.202199 |
|  |  | 7 | -0.063339 | 0.260413 | -0.629004 |
|  |  | 15 | 1.536055 | 0.004559 | -0.744888 |
|  |  | 8 | 2.341799 | 1.042746 | -1.410948 |
|  |  | 9 | 1.689182 | -1.413046 | -1.521658 |
|  |  | 8 | 2.040952 | -0.409542 | 0.732205 |
|  |  | 6 | -2.523859 | 1.295806 | -0.810797 |
|  |  | 6 | -2.233866 | 2.425997 | 0.178941 |
|  |  | 1 | -3.568705 | 1.326970 | -1.128260 |
|  |  | 1 | -1.895219 | 1.416218 | -1.694110 |
|  |  | 1 | -2.447951 | 3.388042 | -0.295145 |
|  |  | 1 | -2.850159 | 2.346482 | 1.077506 |
|  |  | 1 | -1.183123 | 2.418402 | 0.468460 |
|  |  | 6 | -3.464367 | -0.854242 | 0.089035 |
|  |  | 6 | -4.084132 | -0.482032 | 1.439317 |
|  |  | 1 | -3.200701 | -1.909803 | 0.079169 |
|  |  | 1 | -4.203283 | -0.717694 | -0.706584 |
|  |  | 1 | -4.943902 | -1.126474 | 1.643152 |
|  |  | 1 | -3.363740 | -0.604830 | 2.251937 |
|  |  | 1 | -4.432379 | 0.552848 | 1.449492 |
|  |  | 6 | -0.794936 | -1.916468 | 0.353905 |
|  |  | 1 | -1.086612 | -2.670050 | -0.383577 |
|  |  | 1 | 0.254847 | -2.060762 | 0.591137 |
|  |  | 1 | -1.376553 | -2.083841 | 1.261515 |
|  |  | 6 | 3.447608 | -0.318291 | 1.098569 |
|  |  | 6 | 3.723133 | 0.972207 | 1.846583 |
|  |  | 1 | 4.064576 | -0.390570 | 0.200514 |
|  |  | 1 | 3.635064 | -1.193614 | 1.723054 |
|  |  | 1 | 4.768598 | 0.995689 | 2.169360 |
|  |  | 1 | 3.541530 | 1.833876 | 1.201826 |
|  |  | 1 | 3.088036 | 1.049674 | 2.732368 |
|  |  |  |  |  |  |
|  |  |  |  |  |  |
| **26** | -1017.16147 | 7 | 2.171746 | -0.160652 | -0.547498 |
|  |  | 6 | 0.956603 | -0.445673 | -0.018999 |
|  |  | 7 | 0.060067 | 0.500487 | -0.030642 |
|  |  | 15 | -1.477091 | 0.504950 | 0.490713 |
|  |  | 8 | -2.217214 | 1.776076 | 0.405849 |
|  |  | 9 | -1.410320 | -0.002075 | 2.032973 |
|  |  | 8 | -2.198972 | -0.758723 | -0.207454 |
|  |  | 6 | 2.420600 | 1.193202 | -1.083262 |
|  |  | 6 | 2.704148 | 2.251129 | -0.014606 |
|  |  | 1 | 1.543121 | 1.486648 | -1.660976 |
|  |  | 1 | 3.267490 | 1.107899 | -1.768119 |
|  |  | 1 | 2.886579 | 3.215071 | -0.498017 |
|  |  | 1 | 1.846750 | 2.363292 | 0.648672 |
|  |  | 1 | 3.584225 | 1.999903 | 0.582107 |
|  |  | 6 | 3.258241 | -1.140399 | -0.666076 |
|  |  | 6 | 4.357502 | -0.989957 | 0.389778 |
|  |  | 1 | 3.688975 | -1.026726 | -1.665536 |
|  |  | 1 | 2.840688 | -2.144536 | -0.628206 |
|  |  | 1 | 5.120896 | -1.759298 | 0.243196 |
|  |  | 1 | 4.846320 | -0.016109 | 0.322295 |
|  |  | 1 | 3.953519 | -1.096735 | 1.399552 |
|  |  | 6 | 0.731830 | -1.841451 | 0.528418 |
|  |  | 1 | -0.189108 | -1.877197 | 1.101757 |
|  |  | 1 | 0.644226 | -2.558937 | -0.292537 |
|  |  | 1 | 1.553091 | -2.158677 | 1.173195 |
|  |  | 6 | -3.650684 | -0.842396 | -0.288455 |
|  |  | 6 | -4.147892 | -0.323395 | -1.624472 |
|  |  | 1 | -4.095256 | -0.285714 | 0.539362 |
|  |  | 1 | -3.878728 | -1.901734 | -0.158433 |
|  |  | 1 | -5.232387 | -0.455047 | -1.690320 |
|  |  | 1 | -3.921812 | 0.738956 | -1.730522 |
|  |  | 1 | -3.684636 | -0.870658 | -2.449058 |

Table S8: Cartesian coordinates (in Å), total electronic energies (in Hartree) and relative energy (ΔE^ZPE^, kJ.mol^-1^) of selected gas-phase optimised geometries of VR (Russian VX) obtained using the B3LYP/6-311++G(d,p) method.

| VR  Conformers | ΔE^ZPE^  (kJ.mol^-1^) | Total Electronic  Energy (Hartree) | Cartesian Coordinates (in Å) | | | |
| --- | --- | --- | --- | --- | --- | --- |
| **VR 1** | 0 | -1379.877404 | 15 | 1.131989 | 0.314588 | -0.992404 |
|  |  |  | 8 | 0.507176 | -0.991304 | -1.347717 |
|  |  |  | 16 | 0.214249 | 1.200685 | 0.726479 |
|  |  |  | 8 | 2.708740 | 0.279819 | -0.622073 |
|  |  |  | 6 | 1.159539 | 1.559982 | -2.306708 |
|  |  |  | 1 | 1.745105 | 1.169635 | -3.141233 |
|  |  |  | 1 | 0.135663 | 1.745374 | -2.632114 |
|  |  |  | 1 | 1.602684 | 2.487986 | -1.944145 |
|  |  |  | 6 | 3.238811 | -0.702572 | 0.304817 |
|  |  |  | 1 | 2.949122 | -1.698054 | -0.041134 |
|  |  |  | 1 | 2.794003 | -0.527036 | 1.290969 |
|  |  |  | 6 | -1.287605 | 0.129895 | 0.882251 |
|  |  |  | 6 | -2.488352 | 0.657211 | 0.094402 |
|  |  |  | 1 | -1.514280 | 0.107521 | 1.948851 |
|  |  |  | 1 | -1.002231 | -0.870905 | 0.562977 |
|  |  |  | 1 | -2.748472 | 1.646787 | 0.474517 |
|  |  |  | 1 | -2.212165 | 0.789512 | -0.965307 |
|  |  |  | 7 | -3.640187 | -0.233862 | 0.241567 |
|  |  |  | 6 | -3.584736 | -1.361410 | -0.701712 |
|  |  |  | 6 | -4.342561 | -2.595414 | -0.213960 |
|  |  |  | 1 | -2.535834 | -1.629421 | -0.846588 |
|  |  |  | 1 | -3.960396 | -1.054641 | -1.695190 |
|  |  |  | 1 | -4.246108 | -3.406968 | -0.941112 |
|  |  |  | 1 | -3.939437 | -2.937596 | 0.742332 |
|  |  |  | 1 | -5.409845 | -2.401363 | -0.079187 |
|  |  |  | 6 | -4.920574 | 0.481843 | 0.191505 |
|  |  |  | 6 | -5.242413 | 1.238302 | 1.480179 |
|  |  |  | 1 | -5.706980 | -0.256191 | 0.019933 |
|  |  |  | 1 | -4.953499 | 1.172826 | -0.671574 |
|  |  |  | 1 | -6.216255 | 1.729899 | 1.396864 |
|  |  |  | 1 | -5.271726 | 0.549930 | 2.328228 |
|  |  |  | 1 | -4.503626 | 2.013292 | 1.700653 |
|  |  |  | 6 | 4.756190 | -0.560652 | 0.359942 |
|  |  |  | 6 | 5.339775 | -1.688842 | 1.222263 |
|  |  |  | 6 | 5.181391 | 0.819586 | 0.874033 |
|  |  |  | 1 | 5.127312 | -0.681366 | -0.664986 |
|  |  |  | 1 | 5.073687 | -2.675547 | 0.832234 |
|  |  |  | 1 | 6.430482 | -1.624771 | 1.251905 |
|  |  |  | 1 | 4.977644 | -1.625585 | 2.254049 |
|  |  |  | 1 | 4.767211 | 1.617455 | 0.254888 |
|  |  |  | 1 | 4.836601 | 0.974874 | 1.902310 |
|  |  |  | 1 | 6.270715 | 0.915923 | 0.869536 |
|  |  |  |  |  |  |  |
|  |  |  |  |  |  |  |
| **VR 2** | 0.06 | -1379.877455 | 15 | -1.140537 | -0.565582 | -0.782523 |
|  |  |  | 8 | -0.578683 | 0.679740 | -1.378175 |
|  |  |  | 16 | -0.121739 | -1.131326 | 1.012586 |
|  |  |  | 8 | -2.699133 | -0.515352 | -0.343134 |
|  |  |  | 6 | -1.181110 | -2.010452 | -1.873295 |
|  |  |  | 1 | -1.816228 | -1.779030 | -2.730552 |
|  |  |  | 1 | -0.166596 | -2.216905 | -2.215166 |
|  |  |  | 1 | -1.573868 | -2.878751 | -1.343485 |
|  |  |  | 6 | -3.216872 | 0.578819 | 0.456664 |
|  |  |  | 1 | -3.030339 | 1.518078 | -0.073559 |
|  |  |  | 1 | -2.677906 | 0.597450 | 1.408877 |
|  |  |  | 6 | 1.350026 | -0.011114 | 0.924249 |
|  |  |  | 6 | 2.538016 | -0.632640 | 0.186859 |
|  |  |  | 1 | 1.613987 | 0.196602 | 1.961926 |
|  |  |  | 1 | 1.024147 | 0.915164 | 0.454297 |
|  |  |  | 1 | 2.856029 | -1.526800 | 0.726469 |
|  |  |  | 1 | 2.223217 | -0.965831 | -0.816243 |
|  |  |  | 7 | 3.653211 | 0.312113 | 0.113486 |
|  |  |  | 6 | 3.517676 | 1.234050 | -1.024845 |
|  |  |  | 6 | 4.216574 | 2.574795 | -0.802879 |
|  |  |  | 1 | 2.453131 | 1.416926 | -1.187196 |
|  |  |  | 1 | 3.890664 | 0.762172 | -1.952293 |
|  |  |  | 1 | 4.068598 | 3.223955 | -1.670863 |
|  |  |  | 1 | 3.808624 | 3.076407 | 0.077938 |
|  |  |  | 1 | 5.294194 | 2.462133 | -0.657238 |
|  |  |  | 6 | 4.963775 | -0.346338 | 0.155816 |
|  |  |  | 6 | 5.357032 | -0.821252 | 1.554326 |
|  |  |  | 1 | 5.709036 | 0.375438 | -0.185346 |
|  |  |  | 1 | 5.005550 | -1.191491 | -0.556482 |
|  |  |  | 1 | 6.349906 | -1.280311 | 1.533046 |
|  |  |  | 1 | 5.377836 | 0.020831 | 2.250302 |
|  |  |  | 1 | 4.661185 | -1.566649 | 1.948393 |
|  |  |  | 6 | -4.709333 | 0.362618 | 0.682482 |
|  |  |  | 6 | -5.489636 | 0.358851 | -0.637047 |
|  |  |  | 6 | -5.236196 | 1.434230 | 1.647460 |
|  |  |  | 1 | -4.824027 | -0.618532 | 1.159082 |
|  |  |  | 1 | -5.113783 | -0.408661 | -1.316053 |
|  |  |  | 1 | -6.551314 | 0.166030 | -0.459253 |
|  |  |  | 1 | -5.405653 | 1.327877 | -1.141271 |
|  |  |  | 1 | -4.707647 | 1.411822 | 2.604997 |
|  |  |  | 1 | -5.123392 | 2.437903 | 1.223818 |
|  |  |  | 1 | -6.299131 | 1.279820 | 1.849639 |
|  |  |  |  |  |  |  |
|  |  |  |  |  |  |  |
| **VR 3** | 1.62 | -1379.877327 | 15 | 0.573631 | 0.385724 | 0.352414 |
|  |  |  | 8 | 0.228441 | 1.622529 | -0.405658 |
|  |  |  | 16 | 0.040804 | -1.396114 | -0.701175 |
|  |  |  | 8 | 2.149233 | 0.155676 | 0.660496 |
|  |  |  | 6 | -0.098838 | 0.271178 | 2.026301 |
|  |  |  | 1 | 0.227485 | 1.143767 | 2.595545 |
|  |  |  | 1 | -1.184706 | 0.260332 | 1.937884 |
|  |  |  | 1 | 0.246871 | -0.642160 | 2.511061 |
|  |  |  | 6 | 3.149213 | 0.351043 | -0.371663 |
|  |  |  | 1 | 3.003706 | 1.339475 | -0.814894 |
|  |  |  | 1 | 3.003896 | -0.408023 | -1.148625 |
|  |  |  | 6 | -1.258147 | -0.745116 | -1.845773 |
|  |  |  | 6 | -2.692541 | -0.898276 | -1.340179 |
|  |  |  | 1 | -1.133044 | -1.327968 | -2.760800 |
|  |  |  | 1 | -0.996708 | 0.289571 | -2.066228 |
|  |  |  | 1 | -3.368419 | -0.745275 | -2.204670 |
|  |  |  | 1 | -2.836050 | -1.930972 | -1.017497 |
|  |  |  | 7 | -3.053131 | -0.004437 | -0.237793 |
|  |  |  | 6 | -4.200723 | -0.530751 | 0.518499 |
|  |  |  | 6 | -3.843955 | -1.672185 | 1.470422 |
|  |  |  | 1 | -5.004843 | -0.852078 | -0.169697 |
|  |  |  | 1 | -4.616694 | 0.289230 | 1.106580 |
|  |  |  | 1 | -4.739996 | -2.010487 | 1.999091 |
|  |  |  | 1 | -3.424379 | -2.535803 | 0.949321 |
|  |  |  | 1 | -3.112325 | -1.343830 | 2.212330 |
|  |  |  | 6 | -3.309448 | 1.363229 | -0.725688 |
|  |  |  | 6 | -3.196064 | 2.437870 | 0.353591 |
|  |  |  | 1 | -4.300728 | 1.413175 | -1.213774 |
|  |  |  | 1 | -2.569959 | 1.588704 | -1.495530 |
|  |  |  | 1 | -3.425104 | 3.416828 | -0.077432 |
|  |  |  | 1 | -3.889903 | 2.277280 | 1.183071 |
|  |  |  | 1 | -2.177937 | 2.472928 | 0.744097 |
|  |  |  | 6 | 4.534756 | 0.228566 | 0.253753 |
|  |  |  | 6 | 5.598339 | 0.567660 | -0.800240 |
|  |  |  | 6 | 4.765812 | -1.159855 | 0.861253 |
|  |  |  | 1 | 4.592367 | 0.974170 | 1.055985 |
|  |  |  | 1 | 5.458765 | 1.573289 | -1.207537 |
|  |  |  | 1 | 6.599888 | 0.519629 | -0.365135 |
|  |  |  | 1 | 5.567269 | -0.140893 | -1.634950 |
|  |  |  | 1 | 4.009496 | -1.393068 | 1.612884 |
|  |  |  | 1 | 4.725236 | -1.934951 | 0.087856 |
|  |  |  | 1 | 5.748583 | -1.215679 | 1.337865 |
|  |  |  |  |  |  |  |
|  |  |  |  |  |  |  |
| **VR 4** | 1.81 | -1379.877327 | 15 | 0.591193 | 0.395591 | 0.130853 |
|  |  |  | 8 | 0.302494 | 0.978794 | -1.210753 |
|  |  |  | 16 | -0.071487 | -1.629662 | 0.299681 |
|  |  |  | 8 | 2.156909 | 0.292486 | 0.542351 |
|  |  |  | 6 | -0.047307 | 1.319878 | 1.547788 |
|  |  |  | 1 | 0.362133 | 2.331554 | 1.517141 |
|  |  |  | 1 | -1.132588 | 1.348901 | 1.456185 |
|  |  |  | 1 | 0.237272 | 0.830276 | 2.479465 |
|  |  |  | 6 | 3.141770 | -0.218434 | -0.391172 |
|  |  |  | 1 | 3.113179 | 0.392846 | -1.298844 |
|  |  |  | 1 | 2.875014 | -1.247214 | -0.651885 |
|  |  |  | 6 | -1.322396 | -1.693719 | -1.061521 |
|  |  |  | 6 | -2.762156 | -1.446973 | -0.612909 |
|  |  |  | 1 | -1.237187 | -2.704592 | -1.465928 |
|  |  |  | 1 | -0.991938 | -0.993945 | -1.828621 |
|  |  |  | 1 | -3.428376 | -1.773886 | -1.435630 |
|  |  |  | 1 | -2.974740 | -2.102356 | 0.233307 |
|  |  |  | 7 | -3.057170 | -0.066206 | -0.225766 |
|  |  |  | 6 | -4.234937 | -0.003453 | 0.654291 |
|  |  |  | 6 | -3.953734 | -0.429694 | 2.095044 |
|  |  |  | 1 | -5.063029 | -0.607354 | 0.238254 |
|  |  |  | 1 | -4.589301 | 1.028741 | 0.664583 |
|  |  |  | 1 | -4.868109 | -0.354864 | 2.690956 |
|  |  |  | 1 | -3.599685 | -1.460799 | 2.164692 |
|  |  |  | 1 | -3.195615 | 0.213154 | 2.548265 |
|  |  |  | 6 | -3.221687 | 0.792528 | -1.413095 |
|  |  |  | 6 | -3.055392 | 2.284130 | -1.130225 |
|  |  |  | 1 | -4.201937 | 0.600742 | -1.888777 |
|  |  |  | 1 | -2.459132 | 0.508417 | -2.139537 |
|  |  |  | 1 | -3.212263 | 2.851530 | -2.052153 |
|  |  |  | 1 | -3.771469 | 2.656996 | -0.393207 |
|  |  |  | 1 | -2.043515 | 2.490354 | -0.778367 |
|  |  |  | 6 | 4.517481 | -0.165194 | 0.264717 |
|  |  |  | 6 | 4.923698 | 1.269488 | 0.620535 |
|  |  |  | 6 | 5.546495 | -0.830979 | -0.659770 |
|  |  |  | 1 | 4.455477 | -0.749539 | 1.190882 |
|  |  |  | 1 | 4.189884 | 1.734722 | 1.281122 |
|  |  |  | 1 | 5.894011 | 1.283866 | 1.124938 |
|  |  |  | 1 | 5.006742 | 1.886190 | -0.281269 |
|  |  |  | 1 | 5.277781 | -1.867339 | -0.884577 |
|  |  |  | 1 | 5.631957 | -0.291716 | -1.609253 |
|  |  |  | 1 | 6.535263 | -0.836237 | -0.193940 |
|  |  |  |  |  |  |  |
|  |  |  |  |  |  |  |
| **VR 5** | 1.82 | -1379.876918 | 15 | -1.153710 | 0.119623 | -0.746214 |
|  |  |  | 8 | -0.528585 | 1.446675 | -0.481183 |
|  |  |  | 16 | -0.177418 | -1.454346 | 0.325154 |
|  |  |  | 8 | -2.711232 | -0.039572 | -0.336500 |
|  |  |  | 6 | -1.249634 | -0.368685 | -2.487762 |
|  |  |  | 1 | -1.853846 | 0.368204 | -3.020479 |
|  |  |  | 1 | -0.241201 | -0.383243 | -2.901840 |
|  |  |  | 1 | -1.699259 | -1.357440 | -2.583066 |
|  |  |  | 6 | -3.201537 | 0.393924 | 0.960706 |
|  |  |  | 1 | -2.877698 | 1.424635 | 1.127562 |
|  |  |  | 1 | -2.758066 | -0.247389 | 1.728813 |
|  |  |  | 6 | 1.374411 | -0.579043 | 0.828860 |
|  |  |  | 6 | 2.477757 | -0.643237 | -0.229203 |
|  |  |  | 1 | 1.697123 | -1.074812 | 1.745272 |
|  |  |  | 1 | 1.101714 | 0.448052 | 1.064334 |
|  |  |  | 1 | 2.762306 | -1.686932 | -0.375543 |
|  |  |  | 1 | 2.089859 | -0.283453 | -1.197117 |
|  |  |  | 7 | 3.649277 | 0.123431 | 0.195678 |
|  |  |  | 6 | 3.503659 | 1.556967 | -0.104976 |
|  |  |  | 6 | 4.309386 | 2.454742 | 0.832926 |
|  |  |  | 1 | 2.446422 | 1.816795 | -0.018138 |
|  |  |  | 1 | 3.781256 | 1.761473 | -1.155227 |
|  |  |  | 1 | 4.153179 | 3.505882 | 0.573243 |
|  |  |  | 1 | 3.995871 | 2.304004 | 1.868750 |
|  |  |  | 1 | 5.383205 | 2.258258 | 0.774507 |
|  |  |  | 6 | 4.908427 | -0.431614 | -0.314306 |
|  |  |  | 6 | 5.366453 | -1.684742 | 0.431318 |
|  |  |  | 1 | 5.677724 | 0.335566 | -0.202769 |
|  |  |  | 1 | 4.837802 | -0.640608 | -1.398218 |
|  |  |  | 1 | 6.319164 | -2.038664 | 0.026135 |
|  |  |  | 1 | 5.499935 | -1.468281 | 1.493956 |
|  |  |  | 1 | 4.649499 | -2.505117 | 0.341885 |
|  |  |  | 6 | -4.724118 | 0.288980 | 0.978626 |
|  |  |  | 6 | -5.199469 | -1.159269 | 0.801622 |
|  |  |  | 6 | -5.373010 | 1.232567 | -0.042564 |
|  |  |  | 1 | -5.013968 | 0.622891 | 1.984429 |
|  |  |  | 1 | -4.762867 | -1.818087 | 1.558148 |
|  |  |  | 1 | -6.287521 | -1.222862 | 0.888994 |
|  |  |  | 1 | -4.916869 | -1.542869 | -0.181821 |
|  |  |  | 1 | -5.052810 | 2.267356 | 0.110902 |
|  |  |  | 1 | -5.104193 | 0.945291 | -1.062083 |
|  |  |  | 1 | -6.462783 | 1.202146 | 0.040349 |
|  |  |  |  |  |  |  |
|  |  |  |  |  |  |  |
| **VR 6** | 1.97 | -1379.876585 | 15 | 1.138077 | -0.950439 | -0.338722 |
|  |  |  | 8 | 0.491745 | -1.126787 | 0.992484 |
|  |  |  | 16 | 0.132437 | 0.513761 | -1.533345 |
|  |  |  | 8 | 2.685831 | -0.471581 | -0.332278 |
|  |  |  | 6 | 1.294031 | -2.446912 | -1.346565 |
|  |  |  | 1 | 1.920442 | -3.161297 | -0.808891 |
|  |  |  | 1 | 0.300847 | -2.870968 | -1.496335 |
|  |  |  | 1 | 1.743048 | -2.214090 | -2.312643 |
|  |  |  | 6 | 3.117089 | 0.665911 | 0.457300 |
|  |  |  | 1 | 2.889779 | 0.467562 | 1.509602 |
|  |  |  | 1 | 2.553897 | 1.546925 | 0.133719 |
|  |  |  | 6 | -1.339355 | 0.866478 | -0.464878 |
|  |  |  | 6 | -2.435853 | -0.196183 | -0.555910 |
|  |  |  | 1 | -1.689184 | 1.837836 | -0.819518 |
|  |  |  | 1 | -1.002517 | 0.971570 | 0.564481 |
|  |  |  | 1 | -2.670195 | -0.410784 | -1.611705 |
|  |  |  | 1 | -2.045537 | -1.116530 | -0.120204 |
|  |  |  | 7 | -3.625868 | 0.219298 | 0.189618 |
|  |  |  | 6 | -4.283794 | -0.899091 | 0.877137 |
|  |  |  | 6 | -3.548881 | -1.335587 | 2.144418 |
|  |  |  | 1 | -4.408921 | -1.759983 | 0.194466 |
|  |  |  | 1 | -5.291594 | -0.578574 | 1.150012 |
|  |  |  | 1 | -4.065252 | -2.181645 | 2.608215 |
|  |  |  | 1 | -2.520413 | -1.643178 | 1.940881 |
|  |  |  | 1 | -3.513320 | -0.514216 | 2.864154 |
|  |  |  | 6 | -4.545975 | 1.013220 | -0.632074 |
|  |  |  | 6 | -5.437237 | 1.950572 | 0.182298 |
|  |  |  | 1 | -5.168890 | 0.359751 | -1.271490 |
|  |  |  | 1 | -3.943862 | 1.618659 | -1.315340 |
|  |  |  | 1 | -6.073017 | 2.540532 | -0.484606 |
|  |  |  | 1 | -6.095264 | 1.406402 | 0.864285 |
|  |  |  | 1 | -4.827709 | 2.634501 | 0.777906 |
|  |  |  | 6 | 4.613357 | 0.872406 | 0.247269 |
|  |  |  | 6 | 5.425573 | -0.341031 | 0.714324 |
|  |  |  | 6 | 5.054925 | 2.156934 | 0.962559 |
|  |  |  | 1 | 4.771329 | 1.004349 | -0.830029 |
|  |  |  | 1 | 5.110255 | -1.249610 | 0.198193 |
|  |  |  | 1 | 6.491505 | -0.191461 | 0.521041 |
|  |  |  | 1 | 5.300324 | -0.502362 | 1.790680 |
|  |  |  | 1 | 4.505684 | 3.031211 | 0.601081 |
|  |  |  | 1 | 4.895859 | 2.082361 | 2.043594 |
|  |  |  | 1 | 6.119742 | 2.341003 | 0.798565 |
|  |  |  |  |  |  |  |
|  |  |  |  |  |  |  |
| **VR 7** | 2.11 | -1379.87673 | 15 | -1.022542 | -0.542864 | 0.935545 |
|  |  |  | 8 | -0.392879 | -1.666257 | 0.184072 |
|  |  |  | 16 | -0.067274 | 1.327831 | 0.530712 |
|  |  |  | 8 | -2.591180 | -0.270705 | 0.633487 |
|  |  |  | 6 | -1.093854 | -0.739381 | 2.734029 |
|  |  |  | 1 | -1.697337 | -1.620414 | 2.960666 |
|  |  |  | 1 | -0.080166 | -0.882669 | 3.108889 |
|  |  |  | 1 | -1.536037 | 0.142248 | 3.199077 |
|  |  |  | 6 | -3.084528 | -0.165331 | -0.726481 |
|  |  |  | 1 | -2.787306 | -1.063092 | -1.274682 |
|  |  |  | 1 | -2.620615 | 0.708282 | -1.198666 |
|  |  |  | 6 | 1.383935 | 0.752393 | -0.470088 |
|  |  |  | 6 | 2.560374 | 0.268512 | 0.382808 |
|  |  |  | 1 | 1.667753 | 1.623248 | -1.062399 |
|  |  |  | 1 | 1.038975 | -0.030261 | -1.142238 |
|  |  |  | 1 | 2.825867 | 1.047769 | 1.105116 |
|  |  |  | 1 | 2.241724 | -0.602073 | 0.967013 |
|  |  |  | 7 | 3.720091 | -0.072885 | -0.449965 |
|  |  |  | 6 | 4.016096 | -1.510387 | -0.474107 |
|  |  |  | 6 | 2.933434 | -2.343333 | -1.159113 |
|  |  |  | 1 | 4.186552 | -1.897410 | 0.548825 |
|  |  |  | 1 | 4.957520 | -1.637884 | -1.015965 |
|  |  |  | 1 | 3.230451 | -3.396382 | -1.170031 |
|  |  |  | 1 | 1.968666 | -2.278719 | -0.650648 |
|  |  |  | 1 | 2.798003 | -2.015511 | -2.193553 |
|  |  |  | 6 | 4.900827 | 0.747162 | -0.166382 |
|  |  |  | 6 | 4.730557 | 2.211962 | -0.567909 |
|  |  |  | 1 | 5.734641 | 0.325034 | -0.733803 |
|  |  |  | 1 | 5.186402 | 0.688823 | 0.901531 |
|  |  |  | 1 | 5.652707 | 2.765708 | -0.368850 |
|  |  |  | 1 | 4.500808 | 2.293580 | -1.633390 |
|  |  |  | 1 | 3.929943 | 2.702680 | -0.007834 |
|  |  |  | 6 | -4.602373 | -0.021483 | -0.692432 |
|  |  |  | 6 | -5.145856 | -0.052300 | -2.128035 |
|  |  |  | 6 | -5.037974 | 1.247691 | 0.048749 |
|  |  |  | 1 | -4.995714 | -0.892584 | -0.154691 |
|  |  |  | 1 | -4.868130 | -0.974618 | -2.646454 |
|  |  |  | 1 | -6.236774 | 0.014855 | -2.127445 |
|  |  |  | 1 | -4.764112 | 0.791086 | -2.713438 |
|  |  |  | 1 | -4.650663 | 1.262324 | 1.069195 |
|  |  |  | 1 | -4.672140 | 2.143315 | -0.465345 |
|  |  |  | 1 | -6.128501 | 1.313192 | 0.097602 |
|  |  |  |  |  |  |  |
|  |  |  |  |  |  |  |
| **VR 8** | 2.57 | -1379.876706 | 15 | -1.031443 | 0.627688 | -0.784930 |
|  |  |  | 8 | -0.475535 | 1.515659 | 0.275738 |
|  |  |  | 16 | 0.035230 | -1.221326 | -0.925333 |
|  |  |  | 8 | -2.580477 | 0.185485 | -0.611264 |
|  |  |  | 6 | -1.106404 | 1.336093 | -2.449546 |
|  |  |  | 1 | -1.755074 | 2.213760 | -2.422788 |
|  |  |  | 1 | -0.100207 | 1.632622 | -2.746552 |
|  |  |  | 1 | -1.498583 | 0.606460 | -3.158704 |
|  |  |  | 6 | -3.071291 | -0.347898 | 0.645172 |
|  |  |  | 1 | -2.898191 | 0.393710 | 1.431598 |
|  |  |  | 1 | -2.504526 | -1.253560 | 0.882784 |
|  |  |  | 6 | 1.450363 | -0.882233 | 0.223133 |
|  |  |  | 6 | 2.589202 | -0.091498 | -0.428119 |
|  |  |  | 1 | 1.792464 | -1.871219 | 0.530713 |
|  |  |  | 1 | 1.060345 | -0.360959 | 1.094640 |
|  |  |  | 1 | 2.896670 | -0.596772 | -1.350028 |
|  |  |  | 1 | 2.213970 | 0.894475 | -0.723602 |
|  |  |  | 7 | 3.731650 | 0.052038 | 0.482333 |
|  |  |  | 6 | 3.943285 | 1.432005 | 0.936363 |
|  |  |  | 6 | 2.819738 | 1.958213 | 1.828539 |
|  |  |  | 1 | 4.082174 | 2.115652 | 0.077086 |
|  |  |  | 1 | 4.880257 | 1.447088 | 1.500291 |
|  |  |  | 1 | 3.055278 | 2.975285 | 2.156205 |
|  |  |  | 1 | 1.856199 | 1.993191 | 1.314777 |
|  |  |  | 1 | 2.712218 | 1.329154 | 2.716492 |
|  |  |  | 6 | 4.956683 | -0.577242 | -0.017859 |
|  |  |  | 6 | 4.875700 | -2.102639 | -0.068942 |
|  |  |  | 1 | 5.767992 | -0.293820 | 0.658009 |
|  |  |  | 1 | 5.231249 | -0.189512 | -1.017668 |
|  |  |  | 1 | 5.829342 | -2.518465 | -0.406963 |
|  |  |  | 1 | 4.652675 | -2.507835 | 0.921398 |
|  |  |  | 1 | 4.105516 | -2.451862 | -0.761985 |
|  |  |  | 6 | -4.557004 | -0.660704 | 0.502481 |
|  |  |  | 6 | -5.376525 | 0.597545 | 0.194804 |
|  |  |  | 6 | -5.052163 | -1.359794 | 1.776605 |
|  |  |  | 1 | -4.659558 | -1.358500 | -0.337465 |
|  |  |  | 1 | -5.022233 | 1.086644 | -0.714332 |
|  |  |  | 1 | -6.432549 | 0.348203 | 0.058698 |
|  |  |  | 1 | -5.306354 | 1.318264 | 1.016918 |
|  |  |  | 1 | -4.496834 | -2.281146 | 1.975508 |
|  |  |  | 1 | -4.947693 | -0.708413 | 2.650912 |
|  |  |  | 1 | -6.109536 | -1.621061 | 1.685422 |
|  |  |  |  |  |  |  |
|  |  |  |  |  |  |  |
| **VR 9** | 3.11 | -1379.876804 | 15 | 0.648794 | 0.346311 | 0.089005 |
|  |  |  | 8 | 0.274230 | 1.339739 | -0.957974 |
|  |  |  | 16 | -0.033811 | -1.625333 | -0.376159 |
|  |  |  | 8 | 2.237497 | 0.127716 | 0.326621 |
|  |  |  | 6 | 0.125620 | 0.738650 | 1.774512 |
|  |  |  | 1 | 0.536579 | 1.711560 | 2.051091 |
|  |  |  | 1 | -0.963323 | 0.773799 | 1.775966 |
|  |  |  | 1 | 0.476688 | -0.026530 | 2.467345 |
|  |  |  | 6 | 3.146568 | -0.043599 | -0.792393 |
|  |  |  | 1 | 2.975079 | 0.765528 | -1.507394 |
|  |  |  | 1 | 2.919687 | -0.998351 | -1.276902 |
|  |  |  | 6 | -1.388792 | -1.244827 | -1.576767 |
|  |  |  | 6 | -2.784973 | -1.167278 | -0.959846 |
|  |  |  | 1 | -1.356443 | -2.066489 | -2.295461 |
|  |  |  | 1 | -1.104363 | -0.330363 | -2.096697 |
|  |  |  | 1 | -3.517882 | -1.211821 | -1.789696 |
|  |  |  | 1 | -2.945740 | -2.064705 | -0.360159 |
|  |  |  | 7 | -3.022421 | 0.009385 | -0.121747 |
|  |  |  | 6 | -4.128700 | -0.224521 | 0.820412 |
|  |  |  | 6 | -3.750470 | -1.094225 | 2.019150 |
|  |  |  | 1 | -4.998459 | -0.663839 | 0.296967 |
|  |  |  | 1 | -4.459256 | 0.746657 | 1.192853 |
|  |  |  | 1 | -4.613455 | -1.209144 | 2.681629 |
|  |  |  | 1 | -3.427284 | -2.095726 | 1.725899 |
|  |  |  | 1 | -2.939296 | -0.636076 | 2.589655 |
|  |  |  | 6 | -3.261351 | 1.208178 | -0.946662 |
|  |  |  | 6 | -3.033930 | 2.525513 | -0.207891 |
|  |  |  | 1 | -4.281910 | 1.178976 | -1.372431 |
|  |  |  | 1 | -2.569225 | 1.178935 | -1.789314 |
|  |  |  | 1 | -3.256675 | 3.363421 | -0.874842 |
|  |  |  | 1 | -3.673570 | 2.631229 | 0.672235 |
|  |  |  | 1 | -1.990367 | 2.609465 | 0.098889 |
|  |  |  | 6 | 4.583344 | -0.023261 | -0.277653 |
|  |  |  | 6 | 4.863799 | -1.185483 | 0.684244 |
|  |  |  | 6 | 4.948866 | 1.332043 | 0.341899 |
|  |  |  | 1 | 5.202044 | -0.166402 | -1.174291 |
|  |  |  | 1 | 4.633240 | -2.150045 | 0.222154 |
|  |  |  | 1 | 5.916575 | -1.197862 | 0.979481 |
|  |  |  | 1 | 4.260119 | -1.094303 | 1.590609 |
|  |  |  | 1 | 4.779039 | 2.151609 | -0.362591 |
|  |  |  | 1 | 4.348010 | 1.526025 | 1.233883 |
|  |  |  | 1 | 6.002508 | 1.352624 | 0.633614 |
|  |  |  |  |  |  |  |
|  |  |  |  |  |  |  |
| **VR 10** | 3.17 | -1379.876331 | 15 | 1.231962 | 0.646753 | -0.944572 |
|  |  |  | 8 | 0.439203 | -0.429922 | -1.602718 |
|  |  |  | 16 | 0.170803 | 1.611332 | 0.640404 |
|  |  |  | 8 | 2.615619 | 0.197797 | -0.235356 |
|  |  |  | 6 | 1.851729 | 1.937970 | -2.053876 |
|  |  |  | 1 | 2.484754 | 1.469176 | -2.809794 |
|  |  |  | 1 | 1.000754 | 2.417528 | -2.539042 |
|  |  |  | 1 | 2.421814 | 2.680742 | -1.495691 |
|  |  |  | 6 | 2.674973 | -0.974673 | 0.621300 |
|  |  |  | 1 | 2.279850 | -1.829120 | 0.065569 |
|  |  |  | 1 | 2.041077 | -0.796506 | 1.495494 |
|  |  |  | 6 | -1.582258 | 1.247762 | 0.156695 |
|  |  |  | 6 | -2.043318 | -0.156051 | 0.555093 |
|  |  |  | 1 | -1.699984 | 1.390366 | -0.916610 |
|  |  |  | 1 | -2.157420 | 2.023685 | 0.665617 |
|  |  |  | 1 | -1.447191 | -0.872389 | -0.008705 |
|  |  |  | 1 | -1.836768 | -0.329002 | 1.624471 |
|  |  |  | 7 | -3.460611 | -0.345396 | 0.238829 |
|  |  |  | 6 | -4.339040 | 0.098515 | 1.326548 |
|  |  |  | 6 | -5.737377 | 0.500438 | 0.857531 |
|  |  |  | 1 | -3.871835 | 0.967080 | 1.799039 |
|  |  |  | 1 | -4.413311 | -0.674315 | 2.114720 |
|  |  |  | 1 | -6.334087 | 0.849984 | 1.705445 |
|  |  |  | 1 | -5.676461 | 1.304458 | 0.120002 |
|  |  |  | 1 | -6.275228 | -0.334012 | 0.401029 |
|  |  |  | 6 | -3.758800 | -1.707596 | -0.222450 |
|  |  |  | 6 | -3.302227 | -1.965946 | -1.658085 |
|  |  |  | 1 | -4.840422 | -1.850140 | -0.167520 |
|  |  |  | 1 | -3.313864 | -2.457510 | 0.457623 |
|  |  |  | 1 | -3.537902 | -2.994225 | -1.949041 |
|  |  |  | 1 | -3.811775 | -1.286341 | -2.345835 |
|  |  |  | 1 | -2.226110 | -1.823108 | -1.781075 |
|  |  |  | 6 | 4.123270 | -1.214040 | 1.038613 |
|  |  |  | 6 | 4.676010 | -0.054695 | 1.878320 |
|  |  |  | 6 | 5.019673 | -1.523943 | -0.167663 |
|  |  |  | 1 | 4.085213 | -2.108731 | 1.675174 |
|  |  |  | 1 | 4.050706 | 0.136289 | 2.755525 |
|  |  |  | 1 | 5.686904 | -0.279261 | 2.229248 |
|  |  |  | 1 | 4.719954 | 0.865206 | 1.289992 |
|  |  |  | 1 | 4.638804 | -2.375171 | -0.739628 |
|  |  |  | 1 | 5.074509 | -0.664455 | -0.840201 |
|  |  |  | 1 | 6.035620 | -1.764502 | 0.156961 |
|  |  |  |  |  |  |  |
|  |  |  |  |  |  |  |
| **VR 11** | 3.42 | -1379.87661 | 15 | 0.652377 | 0.448434 | 0.222427 |
|  |  |  | 8 | 0.264679 | 1.218739 | -0.993887 |
|  |  |  | 16 | 0.148388 | -1.623298 | 0.078285 |
|  |  |  | 8 | 2.241428 | 0.402396 | 0.551011 |
|  |  |  | 6 | 0.022919 | 1.073203 | 1.798512 |
|  |  |  | 1 | 0.339933 | 2.111518 | 1.914598 |
|  |  |  | 1 | -1.064050 | 1.009351 | 1.762673 |
|  |  |  | 1 | 0.407206 | 0.472145 | 2.623076 |
|  |  |  | 6 | 3.208906 | 0.155744 | -0.500695 |
|  |  |  | 1 | 3.015848 | 0.851706 | -1.320984 |
|  |  |  | 1 | 3.073648 | -0.867785 | -0.868382 |
|  |  |  | 6 | -1.197053 | -1.565259 | -1.187984 |
|  |  |  | 6 | -2.607768 | -1.426444 | -0.613392 |
|  |  |  | 1 | -1.112172 | -2.516170 | -1.718704 |
|  |  |  | 1 | -0.947263 | -0.766337 | -1.885384 |
|  |  |  | 1 | -3.314981 | -1.673070 | -1.428126 |
|  |  |  | 1 | -2.743591 | -2.188189 | 0.158989 |
|  |  |  | 7 | -2.892772 | -0.118681 | -0.026646 |
|  |  |  | 6 | -3.898123 | -0.184009 | 1.043514 |
|  |  |  | 6 | -5.330763 | -0.568964 | 0.636730 |
|  |  |  | 1 | -3.918659 | 0.785796 | 1.547839 |
|  |  |  | 1 | -3.528411 | -0.901145 | 1.783349 |
|  |  |  | 1 | -5.971879 | -0.598297 | 1.522766 |
|  |  |  | 1 | -5.765638 | 0.151429 | -0.060903 |
|  |  |  | 1 | -5.369476 | -1.556489 | 0.168739 |
|  |  |  | 6 | -3.150238 | 0.899264 | -1.057633 |
|  |  |  | 6 | -3.248664 | 2.324672 | -0.519338 |
|  |  |  | 1 | -4.059823 | 0.657891 | -1.637245 |
|  |  |  | 1 | -2.312497 | 0.867848 | -1.755948 |
|  |  |  | 1 | -3.339028 | 3.021419 | -1.356819 |
|  |  |  | 1 | -4.118419 | 2.475033 | 0.125434 |
|  |  |  | 1 | -2.347495 | 2.590437 | 0.036955 |
|  |  |  | 6 | 4.612610 | 0.348949 | 0.063314 |
|  |  |  | 6 | 5.636599 | 0.231703 | -1.074608 |
|  |  |  | 6 | 4.913289 | -0.639274 | 1.196041 |
|  |  |  | 1 | 4.658729 | 1.367478 | 0.467420 |
|  |  |  | 1 | 5.446801 | 0.961274 | -1.867324 |
|  |  |  | 1 | 6.649424 | 0.401491 | -0.700209 |
|  |  |  | 1 | 5.614253 | -0.767007 | -1.523813 |
|  |  |  | 1 | 4.180658 | -0.552105 | 2.000423 |
|  |  |  | 1 | 4.891350 | -1.671092 | 0.828080 |
|  |  |  | 1 | 5.905940 | -0.456703 | 1.617288 |
|  |  |  |  |  |  |  |
|  |  |  |  |  |  |  |
| **VR 12** | 3.52 | -1379.876657 | 15 | -0.672590 | -0.324422 | 0.207719 |
|  |  |  | 8 | -0.357033 | -0.818201 | -1.163282 |
|  |  |  | 16 | -0.018490 | 1.685290 | 0.526127 |
|  |  |  | 8 | -2.249792 | -0.249855 | 0.583987 |
|  |  |  | 6 | -0.071529 | -1.335507 | 1.581627 |
|  |  |  | 1 | -0.467429 | -2.346904 | 1.470075 |
|  |  |  | 1 | 1.016456 | -1.346072 | 1.528804 |
|  |  |  | 1 | -0.396782 | -0.910324 | 2.531506 |
|  |  |  | 6 | -3.211603 | 0.298103 | -0.352310 |
|  |  |  | 1 | -3.079477 | -0.195906 | -1.320176 |
|  |  |  | 1 | -3.009068 | 1.366754 | -0.472436 |
|  |  |  | 6 | 1.284077 | 1.833510 | -0.777826 |
|  |  |  | 6 | 2.696223 | 1.476901 | -0.310569 |
|  |  |  | 1 | 1.253623 | 2.884028 | -1.075480 |
|  |  |  | 1 | 0.957559 | 1.232760 | -1.626201 |
|  |  |  | 1 | 3.395157 | 1.847268 | -1.084538 |
|  |  |  | 1 | 2.912221 | 2.041232 | 0.600523 |
|  |  |  | 7 | 2.898427 | 0.056811 | -0.032830 |
|  |  |  | 6 | 3.931964 | -0.182369 | 0.984002 |
|  |  |  | 6 | 5.380076 | 0.177614 | 0.609997 |
|  |  |  | 1 | 3.889014 | -1.237182 | 1.268335 |
|  |  |  | 1 | 3.638771 | 0.382349 | 1.874730 |
|  |  |  | 1 | 6.043522 | -0.032817 | 1.454086 |
|  |  |  | 1 | 5.737840 | -0.403890 | -0.243545 |
|  |  |  | 1 | 5.485628 | 1.237769 | 0.363490 |
|  |  |  | 6 | 3.050132 | -0.727043 | -1.268957 |
|  |  |  | 6 | 3.067200 | -2.238639 | -1.054209 |
|  |  |  | 1 | 3.955071 | -0.422333 | -1.825680 |
|  |  |  | 1 | 2.193907 | -0.493966 | -1.903484 |
|  |  |  | 1 | 3.071980 | -2.740720 | -2.025211 |
|  |  |  | 1 | 3.949757 | -2.579233 | -0.506573 |
|  |  |  | 1 | 2.173232 | -2.563574 | -0.517943 |
|  |  |  | 6 | -4.617784 | 0.070005 | 0.192412 |
|  |  |  | 6 | -4.934332 | -1.422324 | 0.342451 |
|  |  |  | 6 | -5.634386 | 0.774119 | -0.717363 |
|  |  |  | 1 | -4.659475 | 0.536830 | 1.184106 |
|  |  |  | 1 | -4.214110 | -1.914278 | 0.998635 |
|  |  |  | 1 | -5.933259 | -1.566618 | 0.763723 |
|  |  |  | 1 | -4.905500 | -1.925892 | -0.630177 |
|  |  |  | 1 | -5.434111 | 1.846881 | -0.794208 |
|  |  |  | 1 | -5.616030 | 0.354999 | -1.729122 |
|  |  |  | 1 | -6.648436 | 0.651078 | -0.328428 |
|  |  |  |  |  |  |  |
|  |  |  |  |  |  |  |
| **VR 13** | 3.94 | -1379.876149 | 15 | -1.072043 | 0.383296 | -0.717253 |
|  |  |  | 8 | -0.464925 | 1.571149 | -0.051580 |
|  |  |  | 16 | -0.030718 | -1.424034 | -0.243874 |
|  |  |  | 8 | -2.614714 | 0.061374 | -0.346390 |
|  |  |  | 6 | -1.211041 | 0.478771 | -2.520482 |
|  |  |  | 1 | -1.844340 | 1.331294 | -2.773518 |
|  |  |  | 1 | -0.215136 | 0.625172 | -2.939271 |
|  |  |  | 1 | -1.645452 | -0.438159 | -2.919870 |
|  |  |  | 6 | -3.065423 | 0.007841 | 1.033268 |
|  |  |  | 1 | -2.780119 | 0.939822 | 1.528585 |
|  |  |  | 1 | -2.559514 | -0.827433 | 1.527720 |
|  |  |  | 6 | 1.478126 | -0.734678 | 0.584032 |
|  |  |  | 6 | 2.554415 | -0.265602 | -0.399213 |
|  |  |  | 1 | 1.848605 | -1.555894 | 1.199058 |
|  |  |  | 1 | 1.162632 | 0.075784 | 1.237337 |
|  |  |  | 1 | 2.788324 | -1.081110 | -1.091489 |
|  |  |  | 1 | 2.150750 | 0.552730 | -1.006701 |
|  |  |  | 7 | 3.767543 | 0.171536 | 0.302280 |
|  |  |  | 6 | 3.991237 | 1.620719 | 0.228760 |
|  |  |  | 6 | 2.933025 | 2.440653 | 0.965597 |
|  |  |  | 1 | 4.051852 | 1.959263 | -0.823448 |
|  |  |  | 1 | 4.969058 | 1.819822 | 0.676342 |
|  |  |  | 1 | 3.181076 | 3.504340 | 0.900296 |
|  |  |  | 1 | 1.932404 | 2.310379 | 0.546849 |
|  |  |  | 1 | 2.901543 | 2.161728 | 2.022514 |
|  |  |  | 6 | 4.957435 | -0.604927 | -0.056809 |
|  |  |  | 6 | 4.906531 | -2.051527 | 0.433898 |
|  |  |  | 1 | 5.818964 | -0.112002 | 0.401649 |
|  |  |  | 1 | 5.133874 | -0.591974 | -1.149496 |
|  |  |  | 1 | 5.832266 | -2.570950 | 0.169611 |
|  |  |  | 1 | 4.789077 | -2.084388 | 1.520108 |
|  |  |  | 1 | 4.081102 | -2.610480 | -0.015086 |
|  |  |  | 6 | -4.579640 | -0.182477 | 1.056505 |
|  |  |  | 6 | -4.999055 | -1.514551 | 0.420729 |
|  |  |  | 6 | -5.313878 | 1.007952 | 0.425489 |
|  |  |  | 1 | -4.839095 | -0.216990 | 2.123558 |
|  |  |  | 1 | -4.499421 | -2.361075 | 0.901015 |
|  |  |  | 1 | -6.078090 | -1.664570 | 0.514191 |
|  |  |  | 1 | -4.746732 | -1.534259 | -0.642368 |
|  |  |  | 1 | -5.033786 | 1.949880 | 0.906220 |
|  |  |  | 1 | -5.077231 | 1.090196 | -0.638226 |
|  |  |  | 1 | -6.396549 | 0.889988 | 0.522348 |
|  |  |  |  |  |  |  |
|  |  |  |  |  |  |  |
| **VR 14** | 3.98 | -1379.875775 | 15 | -1.069380 | 0.670606 | -0.582135 |
|  |  |  | 8 | -0.535353 | 1.318536 | 0.649326 |
|  |  |  | 16 | -0.072012 | -1.170463 | -1.020075 |
|  |  |  | 8 | -2.640061 | 0.272594 | -0.562515 |
|  |  |  | 6 | -1.035638 | 1.683241 | -2.083303 |
|  |  |  | 1 | -1.653465 | 2.568058 | -1.918359 |
|  |  |  | 1 | -0.006059 | 1.986096 | -2.275348 |
|  |  |  | 1 | -1.416354 | 1.117732 | -2.934327 |
|  |  |  | 6 | -3.217432 | -0.445595 | 0.557722 |
|  |  |  | 1 | -3.003643 | 0.108692 | 1.477110 |
|  |  |  | 1 | -2.742468 | -1.429540 | 0.619596 |
|  |  |  | 6 | 1.351579 | -1.063114 | 0.160813 |
|  |  |  | 6 | 2.552077 | -0.293152 | -0.397072 |
|  |  |  | 1 | 1.624542 | -2.099008 | 0.366485 |
|  |  |  | 1 | 0.979770 | -0.609435 | 1.077767 |
|  |  |  | 1 | 2.903910 | -0.793608 | -1.301735 |
|  |  |  | 1 | 2.228074 | 0.712284 | -0.703273 |
|  |  |  | 7 | 3.632181 | -0.248168 | 0.586184 |
|  |  |  | 6 | 3.530686 | 0.869585 | 1.532923 |
|  |  |  | 6 | 3.881613 | 2.265519 | 0.990963 |
|  |  |  | 1 | 4.181176 | 0.639263 | 2.383841 |
|  |  |  | 1 | 2.509020 | 0.885893 | 1.921846 |
|  |  |  | 1 | 3.745055 | 3.012795 | 1.778043 |
|  |  |  | 1 | 4.920876 | 2.321409 | 0.655906 |
|  |  |  | 1 | 3.238024 | 2.552177 | 0.155175 |
|  |  |  | 6 | 4.974299 | -0.460160 | 0.041207 |
|  |  |  | 6 | 5.234056 | -1.914831 | -0.353347 |
|  |  |  | 1 | 5.687775 | -0.177737 | 0.821434 |
|  |  |  | 1 | 5.183518 | 0.197169 | -0.821320 |
|  |  |  | 1 | 6.255526 | -2.030020 | -0.728383 |
|  |  |  | 1 | 5.102021 | -2.574198 | 0.507978 |
|  |  |  | 1 | 4.556787 | -2.251632 | -1.142947 |
|  |  |  | 6 | -4.720111 | -0.580690 | 0.335898 |
|  |  |  | 6 | -5.410921 | 0.786420 | 0.273207 |
|  |  |  | 6 | -5.318049 | -1.464976 | 1.439327 |
|  |  |  | 1 | -4.859602 | -1.087030 | -0.626992 |
|  |  |  | 1 | -4.986926 | 1.407536 | -0.517855 |
|  |  |  | 1 | -6.481045 | 0.671271 | 0.079593 |
|  |  |  | 1 | -5.298447 | 1.322608 | 1.221965 |
|  |  |  | 1 | -4.855215 | -2.456000 | 1.459567 |
|  |  |  | 1 | -5.183746 | -1.010297 | 2.426646 |
|  |  |  | 1 | -6.391136 | -1.601490 | 1.283011 |
|  |  |  |  |  |  |  |
|  |  |  |  |  |  |  |
| **VR 15** | 4.15 | -1379.875971 | 15 | 1.106290 | -0.730523 | 0.537929 |
|  |  |  | 8 | 0.522530 | 0.328981 | 1.409036 |
|  |  |  | 16 | 0.190786 | -0.772649 | -1.396435 |
|  |  |  | 8 | 2.689867 | -0.608558 | 0.218518 |
|  |  |  | 6 | 1.063862 | -2.412981 | 1.206928 |
|  |  |  | 1 | 1.646982 | -2.433035 | 2.129635 |
|  |  |  | 1 | 0.028256 | -2.676361 | 1.423385 |
|  |  |  | 1 | 1.480373 | -3.120209 | 0.489045 |
|  |  |  | 6 | 3.272144 | 0.648671 | -0.211667 |
|  |  |  | 1 | 3.059363 | 1.409710 | 0.545807 |
|  |  |  | 1 | 2.799906 | 0.946053 | -1.153098 |
|  |  |  | 6 | -1.260973 | 0.336787 | -1.093260 |
|  |  |  | 6 | -2.517726 | -0.419479 | -0.651117 |
|  |  |  | 1 | -1.437777 | 0.847991 | -2.040262 |
|  |  |  | 1 | -0.953079 | 1.070297 | -0.350235 |
|  |  |  | 1 | -2.825909 | -1.092744 | -1.455457 |
|  |  |  | 1 | -2.277394 | -1.055333 | 0.215992 |
|  |  |  | 7 | -3.602972 | 0.517803 | -0.370018 |
|  |  |  | 6 | -3.543412 | 1.059867 | 0.992985 |
|  |  |  | 6 | -4.316667 | 2.366785 | 1.165487 |
|  |  |  | 1 | -2.491954 | 1.242863 | 1.226034 |
|  |  |  | 1 | -3.890606 | 0.322675 | 1.738244 |
|  |  |  | 1 | -4.153766 | 2.764105 | 2.171268 |
|  |  |  | 1 | -3.978359 | 3.112045 | 0.441049 |
|  |  |  | 1 | -5.394321 | 2.234520 | 1.038753 |
|  |  |  | 6 | -4.924269 | 0.053559 | -0.803885 |
|  |  |  | 6 | -5.505908 | -1.171108 | -0.075730 |
|  |  |  | 1 | -4.854345 | -0.164485 | -1.874504 |
|  |  |  | 1 | -5.621700 | 0.890663 | -0.714931 |
|  |  |  | 1 | -6.478355 | -1.431150 | -0.504607 |
|  |  |  | 1 | -4.856145 | -2.045476 | -0.174051 |
|  |  |  | 1 | -5.656673 | -0.981353 | 0.990011 |
|  |  |  | 6 | 4.774223 | 0.459641 | -0.394145 |
|  |  |  | 6 | 5.460368 | 0.058243 | 0.916796 |
|  |  |  | 6 | 5.379589 | 1.743774 | -0.978141 |
|  |  |  | 1 | 4.910824 | -0.349770 | -1.121590 |
|  |  |  | 1 | 5.028995 | -0.857820 | 1.324452 |
|  |  |  | 1 | 6.529467 | -0.109602 | 0.758980 |
|  |  |  | 1 | 5.353519 | 0.847047 | 1.669616 |
|  |  |  | 1 | 4.917442 | 2.011282 | -1.932853 |
|  |  |  | 1 | 5.250768 | 2.588369 | -0.292899 |
|  |  |  | 1 | 6.451801 | 1.619380 | -1.149560 |

Table S9: Cartesian coordinates (in Å), total electronic energies (in Hartree) and relative energy (ΔE^ZPE^, kJ.mol^-1^) of selected gas-phase optimised geometries of VX obtained using the B3LYP/6-311++G(d,p) method.

| VX  Conformers | ΔE^ZPE^  (kJ.mol^-1^) | Total Electronic  Energy (Hartree) | Cartesian Coordinates (in Å) | | | |
| --- | --- | --- | --- | --- | --- | --- |
| **VX 1** | 0 | -1379.87268 | 15 | 2.236911 | 0.149055 | 0.649703 |
|  |  |  | 8 | 1.579677 | 1.440403 | 0.298896 |
|  |  |  | 16 | 1.297963 | -1.516551 | -0.308127 |
|  |  |  | 8 | 3.800077 | 0.004733 | 0.246658 |
|  |  |  | 6 | 2.351764 | -0.221486 | 2.418696 |
|  |  |  | 1 | 2.940495 | 0.562592 | 2.898673 |
|  |  |  | 1 | 1.345620 | -0.234016 | 2.838285 |
|  |  |  | 1 | 2.826705 | -1.190412 | 2.575133 |
|  |  |  | 6 | 4.272172 | 0.365017 | -1.078285 |
|  |  |  | 6 | 5.778258 | 0.204520 | -1.096072 |
|  |  |  | 1 | 3.974040 | 1.395764 | -1.285162 |
|  |  |  | 1 | 3.792403 | -0.292457 | -1.809468 |
|  |  |  | 1 | 6.164448 | 0.467726 | -2.084824 |
|  |  |  | 1 | 6.247465 | 0.858122 | -0.357600 |
|  |  |  | 1 | 6.062406 | -0.827428 | -0.878825 |
|  |  |  | 6 | -0.262924 | -0.708853 | -0.898147 |
|  |  |  | 6 | -1.367781 | -0.663397 | 0.164964 |
|  |  |  | 1 | -0.581518 | -1.304213 | -1.755057 |
|  |  |  | 1 | 0.001760 | 0.289108 | -1.242254 |
|  |  |  | 1 | -1.621060 | -1.687812 | 0.443262 |
|  |  |  | 1 | -0.972755 | -0.184658 | 1.074224 |
|  |  |  | 7 | -2.549368 | 0.015160 | -0.347321 |
|  |  |  | 6 | -2.487942 | 1.488040 | -0.274165 |
|  |  |  | 6 | -2.966534 | 2.100516 | 1.057071 |
|  |  |  | 6 | -3.201584 | 2.138456 | -1.467123 |
|  |  |  | 1 | -1.426203 | 1.734612 | -0.367535 |
|  |  |  | 1 | -2.452801 | 1.652463 | 1.911701 |
|  |  |  | 1 | -2.756671 | 3.173987 | 1.072860 |
|  |  |  | 1 | -4.044095 | 1.972911 | 1.196446 |
|  |  |  | 1 | -2.814058 | 1.740276 | -2.407434 |
|  |  |  | 1 | -4.281706 | 1.964392 | -1.443595 |
|  |  |  | 1 | -3.047282 | 3.221375 | -1.458052 |
|  |  |  | 6 | -3.849581 | -0.640379 | -0.123778 |
|  |  |  | 6 | -4.132851 | -1.086237 | 1.327257 |
|  |  |  | 6 | -4.031621 | -1.820670 | -1.091070 |
|  |  |  | 1 | -4.606006 | 0.104740 | -0.385124 |
|  |  |  | 1 | -4.020617 | -0.263854 | 2.035040 |
|  |  |  | 1 | -5.156522 | -1.464245 | 1.409405 |
|  |  |  | 1 | -3.464720 | -1.894559 | 1.638701 |
|  |  |  | 1 | -3.918626 | -1.488671 | -2.125114 |
|  |  |  | 1 | -3.290784 | -2.605028 | -0.904508 |
|  |  |  | 1 | -5.021712 | -2.272081 | -0.973964 |
|  |  |  |  |  |  |  |
|  |  |  |  |  |  |  |
| **VX 2** | 0.93 | -1379.87233 | 15 | -2.494507 | -0.778959 | -0.179028 |
|  |  |  | 8 | -1.620176 | -0.966211 | -1.371959 |
|  |  |  | 16 | -1.368972 | -0.678588 | 1.636894 |
|  |  |  | 8 | -3.420587 | 0.552215 | -0.169312 |
|  |  |  | 6 | -3.774884 | -2.035396 | 0.065745 |
|  |  |  | 1 | -4.444245 | -2.019435 | -0.796424 |
|  |  |  | 1 | -3.293007 | -3.010820 | 0.138170 |
|  |  |  | 1 | -4.339764 | -1.836126 | 0.976919 |
|  |  |  | 6 | -2.862286 | 1.856884 | -0.468583 |
|  |  |  | 6 | -3.972804 | 2.879924 | -0.347116 |
|  |  |  | 1 | -2.444803 | 1.832190 | -1.478243 |
|  |  |  | 1 | -2.053711 | 2.061100 | 0.240092 |
|  |  |  | 1 | -3.582276 | 3.876272 | -0.572614 |
|  |  |  | 1 | -4.781517 | 2.660452 | -1.047553 |
|  |  |  | 1 | -4.382199 | 2.890751 | 0.665284 |
|  |  |  | 6 | 0.330179 | -1.060539 | 0.998216 |
|  |  |  | 6 | 1.109173 | 0.193858 | 0.581060 |
|  |  |  | 1 | 0.223776 | -1.753111 | 0.165667 |
|  |  |  | 1 | 0.835615 | -1.570810 | 1.819228 |
|  |  |  | 1 | 0.514430 | 0.753579 | -0.154804 |
|  |  |  | 1 | 1.212298 | 0.840895 | 1.454042 |
|  |  |  | 7 | 2.426289 | -0.161618 | 0.071785 |
|  |  |  | 6 | 2.446442 | -0.541756 | -1.354021 |
|  |  |  | 6 | 3.496489 | -1.626032 | -1.632222 |
|  |  |  | 6 | 2.587216 | 0.630395 | -2.345795 |
|  |  |  | 1 | 1.467540 | -0.992810 | -1.541227 |
|  |  |  | 1 | 3.355059 | -2.476698 | -0.961892 |
|  |  |  | 1 | 3.413433 | -1.979145 | -2.664196 |
|  |  |  | 1 | 4.516787 | -1.253685 | -1.498367 |
|  |  |  | 1 | 1.834155 | 1.401480 | -2.163235 |
|  |  |  | 1 | 3.576895 | 1.092973 | -2.287737 |
|  |  |  | 1 | 2.450005 | 0.273108 | -3.370671 |
|  |  |  | 6 | 3.578632 | 0.577239 | 0.615075 |
|  |  |  | 6 | 3.915573 | 0.085089 | 2.031511 |
|  |  |  | 6 | 3.456057 | 2.116660 | 0.597235 |
|  |  |  | 1 | 4.426269 | 0.314854 | -0.023778 |
|  |  |  | 1 | 4.092445 | -0.992375 | 2.030594 |
|  |  |  | 1 | 4.808635 | 0.586637 | 2.416892 |
|  |  |  | 1 | 3.097225 | 0.293562 | 2.728365 |
|  |  |  | 1 | 3.225793 | 2.495236 | -0.399511 |
|  |  |  | 1 | 2.677730 | 2.468342 | 1.280910 |
|  |  |  | 1 | 4.397944 | 2.570718 | 0.920246 |
|  |  |  |  |  |  |  |
|  |  |  |  |  |  |  |
| **VX 3** | 1.28 | -1379.8724 | 15 | 2.402246 | -0.633818 | 0.486401 |
|  |  |  | 8 | 1.534502 | -0.054708 | 1.551592 |
|  |  |  | 16 | 1.305734 | -1.178811 | -1.264037 |
|  |  |  | 8 | 3.577079 | 0.321952 | -0.090189 |
|  |  |  | 6 | 3.388769 | -2.068818 | 0.984339 |
|  |  |  | 1 | 4.032380 | -1.773879 | 1.815345 |
|  |  |  | 1 | 2.712157 | -2.859856 | 1.309745 |
|  |  |  | 1 | 3.994702 | -2.424390 | 0.150788 |
|  |  |  | 6 | 3.309016 | 1.700624 | -0.457931 |
|  |  |  | 6 | 4.581377 | 2.284449 | -1.036298 |
|  |  |  | 1 | 2.987288 | 2.239669 | 0.436436 |
|  |  |  | 1 | 2.494643 | 1.716229 | -1.188335 |
|  |  |  | 1 | 4.414340 | 3.328962 | -1.314017 |
|  |  |  | 1 | 5.392317 | 2.247833 | -0.305665 |
|  |  |  | 1 | 4.890928 | 1.735350 | -1.928090 |
|  |  |  | 6 | -0.411838 | -1.290270 | -0.567064 |
|  |  |  | 6 | -1.109023 | 0.071851 | -0.444873 |
|  |  |  | 1 | -0.373528 | -1.781455 | 0.403999 |
|  |  |  | 1 | -0.934368 | -1.948813 | -1.263400 |
|  |  |  | 1 | -0.540996 | 0.665507 | 0.269815 |
|  |  |  | 1 | -1.053024 | 0.596177 | -1.410900 |
|  |  |  | 7 | -2.479743 | -0.089525 | 0.018363 |
|  |  |  | 6 | -2.879222 | 0.686768 | 1.206787 |
|  |  |  | 6 | -2.582801 | 2.200926 | 1.140751 |
|  |  |  | 6 | -2.263374 | 0.077662 | 2.476294 |
|  |  |  | 1 | -3.964189 | 0.573886 | 1.284609 |
|  |  |  | 1 | -3.004671 | 2.662014 | 0.246070 |
|  |  |  | 1 | -3.013345 | 2.702639 | 2.012780 |
|  |  |  | 1 | -1.507786 | 2.400328 | 1.153194 |
|  |  |  | 1 | -2.562029 | -0.968051 | 2.580791 |
|  |  |  | 1 | -1.170448 | 0.119158 | 2.447094 |
|  |  |  | 1 | -2.593542 | 0.623017 | 3.366109 |
|  |  |  | 6 | -3.472216 | -0.315540 | -1.044007 |
|  |  |  | 6 | -4.034507 | 0.961018 | -1.703054 |
|  |  |  | 6 | -4.607570 | -1.236767 | -0.575538 |
|  |  |  | 1 | -2.930982 | -0.862265 | -1.824550 |
|  |  |  | 1 | -3.232643 | 1.620553 | -2.045804 |
|  |  |  | 1 | -4.647544 | 0.702730 | -2.572100 |
|  |  |  | 1 | -4.667104 | 1.522985 | -1.010126 |
|  |  |  | 1 | -4.201955 | -2.159181 | -0.154324 |
|  |  |  | 1 | -5.230265 | -0.760401 | 0.187004 |
|  |  |  | 1 | -5.261891 | -1.493194 | -1.414100 |
|  |  |  |  |  |  |  |
|  |  |  |  |  |  |  |
| **VX 4** | 1.54 | -1379.87221 | 15 | -2.282741 | 0.555400 | -0.402514 |
|  |  |  | 8 | -1.567574 | 1.275109 | 0.689750 |
|  |  |  | 16 | -1.272538 | -1.238211 | -0.982599 |
|  |  |  | 8 | -3.799178 | 0.085083 | -0.074068 |
|  |  |  | 6 | -2.589070 | 1.505690 | -1.912990 |
|  |  |  | 1 | -3.227625 | 2.355120 | -1.663277 |
|  |  |  | 1 | -1.634551 | 1.864188 | -2.298981 |
|  |  |  | 1 | -3.077898 | 0.883737 | -2.663605 |
|  |  |  | 6 | -4.112207 | -0.635100 | 1.146279 |
|  |  |  | 6 | -5.609398 | -0.862621 | 1.182708 |
|  |  |  | 1 | -3.773966 | -0.039918 | 1.998113 |
|  |  |  | 1 | -3.564710 | -1.582572 | 1.139695 |
|  |  |  | 1 | -5.877631 | -1.404683 | 2.093888 |
|  |  |  | 1 | -6.146578 | 0.088211 | 1.176592 |
|  |  |  | 1 | -5.934352 | -1.452075 | 0.322603 |
|  |  |  | 6 | 0.266405 | -1.100903 | 0.044323 |
|  |  |  | 6 | 1.314372 | -0.143131 | -0.537810 |
|  |  |  | 1 | 0.640305 | -2.125558 | 0.088409 |
|  |  |  | 1 | -0.015470 | -0.788026 | 1.047766 |
|  |  |  | 1 | 1.491057 | -0.391828 | -1.595260 |
|  |  |  | 1 | 0.890864 | 0.861096 | -0.518153 |
|  |  |  | 7 | 2.538372 | -0.171976 | 0.250321 |
|  |  |  | 6 | 3.503154 | -1.211576 | -0.142001 |
|  |  |  | 6 | 4.285262 | -1.747743 | 1.065451 |
|  |  |  | 6 | 4.457245 | -0.825305 | -1.290757 |
|  |  |  | 1 | 2.889299 | -2.042745 | -0.506979 |
|  |  |  | 1 | 3.600336 | -2.069136 | 1.853034 |
|  |  |  | 1 | 4.903559 | -2.600655 | 0.770143 |
|  |  |  | 1 | 4.955758 | -0.992873 | 1.486355 |
|  |  |  | 1 | 3.906409 | -0.453280 | -2.158805 |
|  |  |  | 1 | 5.166356 | -0.053403 | -0.979084 |
|  |  |  | 1 | 5.037965 | -1.695368 | -1.612318 |
|  |  |  | 6 | 3.051483 | 1.121783 | 0.737837 |
|  |  |  | 6 | 2.191695 | 1.631950 | 1.904788 |
|  |  |  | 6 | 3.209849 | 2.219653 | -0.336455 |
|  |  |  | 1 | 4.048979 | 0.915029 | 1.135309 |
|  |  |  | 1 | 2.172804 | 0.897288 | 2.712945 |
|  |  |  | 1 | 2.590893 | 2.571960 | 2.298824 |
|  |  |  | 1 | 1.159436 | 1.813525 | 1.590928 |
|  |  |  | 1 | 3.806327 | 1.878788 | -1.184392 |
|  |  |  | 1 | 2.240435 | 2.555495 | -0.715166 |
|  |  |  | 1 | 3.706406 | 3.094182 | 0.094773 |
|  |  |  |  |  |  |  |
|  |  |  |  |  |  |  |
| **VX 5** | 2.68 | -1379.87179 | 15 | 2.311933 | -0.197022 | 0.614303 |
|  |  |  | 8 | 1.732760 | 1.175131 | 0.534826 |
|  |  |  | 16 | 1.260671 | -1.573651 | -0.639999 |
|  |  |  | 8 | 3.858890 | -0.364298 | 0.163676 |
|  |  |  | 6 | 2.410307 | -0.913639 | 2.274599 |
|  |  |  | 1 | 3.061437 | -0.284294 | 2.884311 |
|  |  |  | 1 | 1.409469 | -0.932207 | 2.706555 |
|  |  |  | 1 | 2.810048 | -1.926988 | 2.229905 |
|  |  |  | 6 | 4.357541 | 0.179235 | -1.088604 |
|  |  |  | 6 | 4.934338 | 1.569217 | -0.889562 |
|  |  |  | 1 | 3.552741 | 0.178949 | -1.828883 |
|  |  |  | 1 | 5.120391 | -0.527984 | -1.417396 |
|  |  |  | 1 | 5.353479 | 1.930567 | -1.833700 |
|  |  |  | 1 | 4.161477 | 2.266362 | -0.561705 |
|  |  |  | 1 | 5.733148 | 1.552324 | -0.144503 |
|  |  |  | 6 | -0.250020 | -0.571477 | -1.023818 |
|  |  |  | 6 | -1.351825 | -0.693511 | 0.036179 |
|  |  |  | 1 | -0.601989 | -0.949339 | -1.984816 |
|  |  |  | 1 | 0.073959 | 0.460081 | -1.148107 |
|  |  |  | 1 | -1.656534 | -1.739515 | 0.097765 |
|  |  |  | 1 | -0.935380 | -0.433939 | 1.021745 |
|  |  |  | 7 | -2.498828 | 0.132525 | -0.312760 |
|  |  |  | 6 | -3.830128 | -0.490114 | -0.204980 |
|  |  |  | 6 | -4.123542 | -1.217538 | 1.125292 |
|  |  |  | 6 | -4.082675 | -1.428858 | -1.395188 |
|  |  |  | 1 | -4.547768 | 0.330494 | -0.288072 |
|  |  |  | 1 | -3.964694 | -0.569761 | 1.988636 |
|  |  |  | 1 | -5.164514 | -1.554417 | 1.145100 |
|  |  |  | 1 | -3.495371 | -2.104740 | 1.247073 |
|  |  |  | 1 | -3.961103 | -0.891798 | -2.338104 |
|  |  |  | 1 | -3.382582 | -2.270796 | -1.392563 |
|  |  |  | 1 | -5.094217 | -1.844854 | -1.356812 |
|  |  |  | 6 | -2.362568 | 1.553998 | 0.059555 |
|  |  |  | 6 | -2.787959 | 1.900096 | 1.500626 |
|  |  |  | 6 | -3.061218 | 2.470226 | -0.954389 |
|  |  |  | 1 | -1.291421 | 1.765436 | -0.009039 |
|  |  |  | 1 | -2.287977 | 1.258398 | 2.230838 |
|  |  |  | 1 | -2.518465 | 2.934489 | 1.733124 |
|  |  |  | 1 | -3.868934 | 1.802490 | 1.638318 |
|  |  |  | 1 | -2.711106 | 2.258334 | -1.966949 |
|  |  |  | 1 | -4.148520 | 2.347223 | -0.937875 |
|  |  |  | 1 | -2.850204 | 3.519087 | -0.726360 |
|  |  |  |  |  |  |  |
|  |  |  |  |  |  |  |
| **VX 6** | 2.82 | -1379.87144 | 15 | 2.167889 | 0.386103 | 0.594961 |
|  |  |  | 8 | 1.534999 | 1.456719 | -0.227830 |
|  |  |  | 16 | 1.129630 | -1.474770 | 0.430481 |
|  |  |  | 8 | 3.706987 | 0.024846 | 0.235538 |
|  |  |  | 6 | 2.355420 | 0.747623 | 2.359519 |
|  |  |  | 1 | 3.005604 | 1.617907 | 2.467042 |
|  |  |  | 1 | 1.373154 | 0.968916 | 2.777633 |
|  |  |  | 1 | 2.791307 | -0.106279 | 2.879123 |
|  |  |  | 6 | 4.120191 | -0.218310 | -1.134389 |
|  |  |  | 6 | 5.612454 | -0.478447 | -1.132464 |
|  |  |  | 1 | 3.864341 | 0.658012 | -1.735083 |
|  |  |  | 1 | 3.565768 | -1.080718 | -1.516932 |
|  |  |  | 1 | 5.955094 | -0.664041 | -2.154333 |
|  |  |  | 1 | 6.156240 | 0.382512 | -0.737795 |
|  |  |  | 1 | 5.854197 | -1.352404 | -0.523722 |
|  |  |  | 6 | -0.364222 | -0.941718 | -0.534271 |
|  |  |  | 6 | -1.468128 | -0.327825 | 0.342470 |
|  |  |  | 1 | -0.708689 | -1.856410 | -1.017025 |
|  |  |  | 1 | -0.036057 | -0.243256 | -1.300273 |
|  |  |  | 1 | -1.707359 | -1.030513 | 1.146902 |
|  |  |  | 1 | -1.062130 | 0.562357 | 0.830632 |
|  |  |  | 7 | -2.658538 | 0.009738 | -0.426896 |
|  |  |  | 6 | -2.893995 | 1.436417 | -0.717956 |
|  |  |  | 6 | -1.800742 | 2.005750 | -1.634701 |
|  |  |  | 6 | -3.079318 | 2.349737 | 0.514037 |
|  |  |  | 1 | -3.833256 | 1.457889 | -1.281258 |
|  |  |  | 1 | -1.717832 | 1.409823 | -2.546894 |
|  |  |  | 1 | -2.046175 | 3.033700 | -1.918240 |
|  |  |  | 1 | -0.823180 | 2.025118 | -1.144782 |
|  |  |  | 1 | -3.896582 | 2.014885 | 1.155105 |
|  |  |  | 1 | -2.169798 | 2.401116 | 1.119906 |
|  |  |  | 1 | -3.306500 | 3.369938 | 0.190327 |
|  |  |  | 6 | -3.840647 | -0.849968 | -0.256682 |
|  |  |  | 6 | -4.452990 | -0.878914 | 1.161339 |
|  |  |  | 6 | -3.561939 | -2.282145 | -0.739375 |
|  |  |  | 1 | -4.597288 | -0.430772 | -0.928476 |
|  |  |  | 1 | -4.759245 | 0.114934 | 1.490783 |
|  |  |  | 1 | -5.338112 | -1.522375 | 1.177847 |
|  |  |  | 1 | -3.748067 | -1.277205 | 1.897262 |
|  |  |  | 1 | -3.174636 | -2.275929 | -1.760595 |
|  |  |  | 1 | -2.835515 | -2.790441 | -0.097416 |
|  |  |  | 1 | -4.480327 | -2.875882 | -0.720396 |
|  |  |  |  |  |  |  |
|  |  |  |  |  |  |  |
| **VX 7** | 3.32 | -1379.87158 | 15 | 2.460737 | -0.470917 | 0.490388 |
|  |  |  | 8 | 1.654751 | 0.554985 | 1.213076 |
|  |  |  | 16 | 1.281070 | -1.652520 | -0.839729 |
|  |  |  | 8 | 3.652272 | 0.067273 | -0.464418 |
|  |  |  | 6 | 3.403377 | -1.596687 | 1.551259 |
|  |  |  | 1 | 4.090562 | -1.004017 | 2.158189 |
|  |  |  | 1 | 2.707221 | -2.126241 | 2.202749 |
|  |  |  | 1 | 3.960504 | -2.313618 | 0.948320 |
|  |  |  | 6 | 3.444363 | 1.128916 | -1.435400 |
|  |  |  | 6 | 3.837760 | 2.476243 | -0.858186 |
|  |  |  | 1 | 2.401938 | 1.119839 | -1.764858 |
|  |  |  | 1 | 4.071237 | 0.859156 | -2.286621 |
|  |  |  | 1 | 3.725579 | 3.250210 | -1.623733 |
|  |  |  | 1 | 3.204887 | 2.733914 | -0.007206 |
|  |  |  | 1 | 4.880199 | 2.466517 | -0.531568 |
|  |  |  | 6 | -0.401437 | -1.455706 | -0.078920 |
|  |  |  | 6 | -1.088589 | -0.132808 | -0.445772 |
|  |  |  | 1 | -0.322051 | -1.552928 | 1.003038 |
|  |  |  | 1 | -0.958423 | -2.315511 | -0.456289 |
|  |  |  | 1 | -0.486555 | 0.673937 | -0.030685 |
|  |  |  | 1 | -1.076233 | -0.009787 | -1.539390 |
|  |  |  | 7 | -2.437095 | -0.084036 | 0.101159 |
|  |  |  | 6 | -2.778143 | 1.082584 | 0.935699 |
|  |  |  | 6 | -2.486410 | 2.460825 | 0.303318 |
|  |  |  | 6 | -2.098970 | 0.974377 | 2.310175 |
|  |  |  | 1 | -3.857713 | 1.024741 | 1.100666 |
|  |  |  | 1 | -2.949540 | 2.566907 | -0.679018 |
|  |  |  | 1 | -2.876849 | 3.255110 | 0.946956 |
|  |  |  | 1 | -1.412171 | 2.633767 | 0.194954 |
|  |  |  | 1 | -2.385926 | 0.043768 | 2.805388 |
|  |  |  | 1 | -1.009120 | 0.990301 | 2.215381 |
|  |  |  | 1 | -2.388754 | 1.812658 | 2.951546 |
|  |  |  | 6 | -3.478577 | -0.665961 | -0.760068 |
|  |  |  | 6 | -4.065279 | 0.287242 | -1.821413 |
|  |  |  | 6 | -4.595450 | -1.326540 | 0.060624 |
|  |  |  | 1 | -2.975133 | -1.473179 | -1.304127 |
|  |  |  | 1 | -3.276628 | 0.757052 | -2.415145 |
|  |  |  | 1 | -4.716876 | -0.262376 | -2.507762 |
|  |  |  | 1 | -4.664893 | 1.078016 | -1.361673 |
|  |  |  | 1 | -4.175882 | -2.036465 | 0.776718 |
|  |  |  | 1 | -5.183723 | -0.590886 | 0.616692 |
|  |  |  | 1 | -5.286212 | -1.861531 | -0.597934 |
|  |  |  |  |  |  |  |
|  |  |  |  |  |  |  |
| **VX 8** | 3.58 | -1379.8716 | 15 | 2.329392 | -0.663643 | -0.337354 |
|  |  |  | 8 | 1.489327 | -1.420028 | 0.635227 |
|  |  |  | 16 | 1.177078 | 0.625374 | -1.589875 |
|  |  |  | 8 | 3.452633 | 0.321913 | 0.289507 |
|  |  |  | 6 | 3.376238 | -1.679449 | -1.410965 |
|  |  |  | 1 | 4.045619 | -2.270088 | -0.782591 |
|  |  |  | 1 | 2.735722 | -2.345980 | -1.989622 |
|  |  |  | 1 | 3.956142 | -1.050002 | -2.086105 |
|  |  |  | 6 | 3.128496 | 1.243710 | 1.363349 |
|  |  |  | 6 | 4.384059 | 2.017680 | 1.708015 |
|  |  |  | 1 | 2.762696 | 0.666779 | 2.216277 |
|  |  |  | 1 | 2.330181 | 1.909499 | 1.022435 |
|  |  |  | 1 | 4.172690 | 2.719182 | 2.519952 |
|  |  |  | 1 | 5.179012 | 1.343283 | 2.033504 |
|  |  |  | 1 | 4.740127 | 2.585316 | 0.845616 |
|  |  |  | 6 | -0.512943 | -0.121477 | -1.391563 |
|  |  |  | 6 | -1.248918 | 0.376845 | -0.136160 |
|  |  |  | 1 | -0.418924 | -1.205092 | -1.382729 |
|  |  |  | 1 | -1.039470 | 0.168702 | -2.300837 |
|  |  |  | 1 | -0.651505 | 0.099752 | 0.734742 |
|  |  |  | 1 | -1.272762 | 1.471143 | -0.157540 |
|  |  |  | 7 | -2.596078 | -0.172860 | -0.040145 |
|  |  |  | 6 | -3.719182 | 0.732607 | -0.330406 |
|  |  |  | 6 | -3.695226 | 1.198724 | -1.794563 |
|  |  |  | 6 | -3.864293 | 1.950693 | 0.608827 |
|  |  |  | 1 | -4.618799 | 0.120093 | -0.207067 |
|  |  |  | 1 | -3.647548 | 0.342700 | -2.471201 |
|  |  |  | 1 | -4.596435 | 1.773793 | -2.026077 |
|  |  |  | 1 | -2.837351 | 1.847833 | -1.996974 |
|  |  |  | 1 | -3.984424 | 1.647806 | 1.649693 |
|  |  |  | 1 | -2.996292 | 2.614176 | 0.548485 |
|  |  |  | 1 | -4.743260 | 2.540001 | 0.329598 |
|  |  |  | 6 | -2.821893 | -1.229467 | 0.964083 |
|  |  |  | 6 | -2.583005 | -0.819224 | 2.433790 |
|  |  |  | 6 | -2.007727 | -2.490541 | 0.635718 |
|  |  |  | 1 | -3.881182 | -1.491000 | 0.865864 |
|  |  |  | 1 | -3.201652 | 0.029763 | 2.729089 |
|  |  |  | 1 | -2.825468 | -1.654517 | 3.097749 |
|  |  |  | 1 | -1.535808 | -0.559532 | 2.612365 |
|  |  |  | 1 | -2.220661 | -2.830242 | -0.381064 |
|  |  |  | 1 | -0.932501 | -2.313479 | 0.729524 |
|  |  |  | 1 | -2.269041 | -3.296898 | 1.327626 |
|  |  |  |  |  |  |  |
|  |  |  |  |  |  |  |
| **VX 9** | 3.72 | -1379.87147 | 15 | 2.552827 | -0.428936 | 0.466763 |
|  |  |  | 8 | 1.716168 | 0.411387 | 1.371495 |
|  |  |  | 16 | 1.360700 | -1.711947 | -0.758972 |
|  |  |  | 8 | 3.511367 | 0.354144 | -0.578281 |
|  |  |  | 6 | 3.790564 | -1.466550 | 1.285554 |
|  |  |  | 1 | 4.483317 | -0.817592 | 1.824685 |
|  |  |  | 1 | 3.280326 | -2.123552 | 1.990606 |
|  |  |  | 1 | 4.335089 | -2.064269 | 0.554192 |
|  |  |  | 6 | 3.018934 | 1.434765 | -1.414556 |
|  |  |  | 6 | 3.221722 | 2.781414 | -0.743334 |
|  |  |  | 1 | 1.966220 | 1.257452 | -1.652372 |
|  |  |  | 1 | 3.592039 | 1.357838 | -2.339614 |
|  |  |  | 1 | 2.887899 | 3.579513 | -1.413615 |
|  |  |  | 1 | 2.650026 | 2.841755 | 0.184187 |
|  |  |  | 1 | 4.277985 | 2.944200 | -0.516820 |
|  |  |  | 6 | -0.297321 | -1.486958 | 0.044020 |
|  |  |  | 6 | -1.107558 | -0.336861 | -0.568592 |
|  |  |  | 1 | -0.134294 | -1.328045 | 1.108156 |
|  |  |  | 1 | -0.813745 | -2.437762 | -0.094392 |
|  |  |  | 1 | -0.500202 | 0.578712 | -0.540570 |
|  |  |  | 1 | -1.282595 | -0.561868 | -1.622379 |
|  |  |  | 7 | -2.378337 | -0.177098 | 0.123997 |
|  |  |  | 6 | -3.594632 | -0.105352 | -0.702844 |
|  |  |  | 6 | -3.980792 | -1.501131 | -1.217287 |
|  |  |  | 6 | -3.552095 | 0.903987 | -1.871141 |
|  |  |  | 1 | -4.392174 | 0.218098 | -0.028370 |
|  |  |  | 1 | -4.099763 | -2.196643 | -0.384010 |
|  |  |  | 1 | -4.918731 | -1.464606 | -1.780023 |
|  |  |  | 1 | -3.213132 | -1.902748 | -1.886718 |
|  |  |  | 1 | -3.280135 | 1.905363 | -1.534587 |
|  |  |  | 1 | -2.837913 | 0.600142 | -2.641986 |
|  |  |  | 1 | -4.534861 | 0.964687 | -2.348845 |
|  |  |  | 6 | -2.307368 | 0.656432 | 1.339732 |
|  |  |  | 6 | -3.280630 | 0.162484 | 2.418895 |
|  |  |  | 6 | -2.464634 | 2.171970 | 1.105962 |
|  |  |  | 1 | -1.296577 | 0.504130 | 1.729350 |
|  |  |  | 1 | -3.126108 | -0.900579 | 2.616417 |
|  |  |  | 1 | -3.126385 | 0.715013 | 3.350331 |
|  |  |  | 1 | -4.325552 | 0.305511 | 2.126795 |
|  |  |  | 1 | -1.761981 | 2.533925 | 0.350872 |
|  |  |  | 1 | -3.478897 | 2.428899 | 0.786450 |
|  |  |  | 1 | -2.262526 | 2.717857 | 2.032223 |
|  |  |  |  |  |  |  |
|  |  |  |  |  |  |  |
| **VX 10** | 4.00 | -1379.87112 | 15 | 2.348163 | 0.167129 | 0.615758 |
|  |  |  | 8 | 1.710016 | 1.248005 | -0.189814 |
|  |  |  | 16 | 1.249008 | -1.663343 | 0.504389 |
|  |  |  | 8 | 3.861352 | -0.246505 | 0.210779 |
|  |  |  | 6 | 2.608460 | 0.537136 | 2.369468 |
|  |  |  | 1 | 3.291789 | 1.385034 | 2.445915 |
|  |  |  | 1 | 1.649483 | 0.797075 | 2.818228 |
|  |  |  | 1 | 3.031974 | -0.326335 | 2.883258 |
|  |  |  | 6 | 4.235438 | -0.554525 | -1.159088 |
|  |  |  | 6 | 4.683639 | 0.691191 | -1.902499 |
|  |  |  | 1 | 3.395140 | -1.043404 | -1.660536 |
|  |  |  | 1 | 5.044361 | -1.280999 | -1.070154 |
|  |  |  | 1 | 5.005789 | 0.418494 | -2.912218 |
|  |  |  | 1 | 3.867916 | 1.411923 | -1.978135 |
|  |  |  | 1 | 5.525023 | 1.164265 | -1.390739 |
|  |  |  | 6 | -0.262833 | -1.091336 | -0.405441 |
|  |  |  | 6 | -1.277359 | -0.353936 | 0.478301 |
|  |  |  | 1 | -0.681874 | -2.013125 | -0.813686 |
|  |  |  | 1 | 0.052590 | -0.454818 | -1.229818 |
|  |  |  | 1 | -1.494906 | -0.960253 | 1.370761 |
|  |  |  | 1 | -0.803825 | 0.562943 | 0.829701 |
|  |  |  | 7 | -2.478049 | -0.021364 | -0.274199 |
|  |  |  | 6 | -2.914575 | 1.386811 | -0.254239 |
|  |  |  | 6 | -2.005389 | 2.242386 | -1.150076 |
|  |  |  | 6 | -3.043067 | 2.020822 | 1.147952 |
|  |  |  | 1 | -3.912282 | 1.393876 | -0.701615 |
|  |  |  | 1 | -2.013827 | 1.862546 | -2.174190 |
|  |  |  | 1 | -2.342527 | 3.283700 | -1.162835 |
|  |  |  | 1 | -0.970376 | 2.230589 | -0.795665 |
|  |  |  | 1 | -3.671979 | 1.423638 | 1.810377 |
|  |  |  | 1 | -2.067083 | 2.144829 | 1.625332 |
|  |  |  | 1 | -3.489191 | 3.016729 | 1.067241 |
|  |  |  | 6 | -3.502931 | -1.076386 | -0.312376 |
|  |  |  | 6 | -4.253441 | -1.090196 | -1.651876 |
|  |  |  | 6 | -4.489476 | -1.079316 | 0.873355 |
|  |  |  | 1 | -2.943414 | -2.017133 | -0.260646 |
|  |  |  | 1 | -3.548605 | -1.144562 | -2.484333 |
|  |  |  | 1 | -4.924167 | -1.952903 | -1.704668 |
|  |  |  | 1 | -4.866878 | -0.194421 | -1.785358 |
|  |  |  | 1 | -3.963332 | -1.085890 | 1.831753 |
|  |  |  | 1 | -5.145794 | -0.204749 | 0.849389 |
|  |  |  | 1 | -5.125966 | -1.968851 | 0.836839 |
|  |  |  |  |  |  |  |
|  |  |  |  |  |  |  |
| **VX 11** | 4.29 | -1379.8718 | 15 | 2.231170 | 0.094416 | 0.705433 |
|  |  |  | 8 | 1.583829 | 1.421574 | 0.498087 |
|  |  |  | 16 | 1.222876 | -1.466091 | -0.350811 |
|  |  |  | 8 | 3.775077 | -0.030399 | 0.227840 |
|  |  |  | 6 | 2.408934 | -0.437624 | 2.427160 |
|  |  |  | 1 | 3.029611 | 0.291524 | 2.951435 |
|  |  |  | 1 | 1.419866 | -0.474559 | 2.884363 |
|  |  |  | 1 | 2.873226 | -1.423028 | 2.476375 |
|  |  |  | 6 | 4.192889 | 0.428113 | -1.084876 |
|  |  |  | 6 | 5.692637 | 0.242571 | -1.187962 |
|  |  |  | 1 | 3.907688 | 1.477367 | -1.193591 |
|  |  |  | 1 | 3.664969 | -0.159386 | -1.842098 |
|  |  |  | 1 | 6.038458 | 0.578457 | -2.169619 |
|  |  |  | 1 | 6.209705 | 0.825243 | -0.422559 |
|  |  |  | 1 | 5.963828 | -0.808624 | -1.068345 |
|  |  |  | 6 | -0.310283 | -0.573938 | -0.896365 |
|  |  |  | 6 | -1.374363 | -0.457192 | 0.201346 |
|  |  |  | 1 | -0.682228 | -1.171944 | -1.728957 |
|  |  |  | 1 | -0.015543 | 0.402654 | -1.272794 |
|  |  |  | 1 | -1.549216 | -1.451259 | 0.619996 |
|  |  |  | 1 | -0.978702 | 0.151690 | 1.021035 |
|  |  |  | 7 | -2.623598 | 0.116818 | -0.316344 |
|  |  |  | 6 | -3.776438 | -0.809552 | -0.258059 |
|  |  |  | 6 | -4.220569 | -1.195066 | 1.167947 |
|  |  |  | 6 | -3.533105 | -2.053644 | -1.122048 |
|  |  |  | 1 | -4.606802 | -0.280944 | -0.726993 |
|  |  |  | 1 | -4.429928 | -0.309750 | 1.774188 |
|  |  |  | 1 | -5.132923 | -1.797942 | 1.133365 |
|  |  |  | 1 | -3.458408 | -1.784033 | 1.686802 |
|  |  |  | 1 | -3.265911 | -1.765024 | -2.141373 |
|  |  |  | 1 | -2.737865 | -2.691009 | -0.724797 |
|  |  |  | 1 | -4.441691 | -2.661096 | -1.164925 |
|  |  |  | 6 | -2.870997 | 1.499709 | 0.160086 |
|  |  |  | 6 | -4.207658 | 2.065214 | -0.337133 |
|  |  |  | 6 | -1.740535 | 2.437627 | -0.288537 |
|  |  |  | 1 | -2.894248 | 1.514500 | 1.266284 |
|  |  |  | 1 | -5.076899 | 1.571606 | 0.101600 |
|  |  |  | 1 | -4.268067 | 3.122533 | -0.068280 |
|  |  |  | 1 | -4.278173 | 1.991050 | -1.426868 |
|  |  |  | 1 | -0.756334 | 2.133768 | 0.070026 |
|  |  |  | 1 | -1.709376 | 2.490700 | -1.381494 |
|  |  |  | 1 | -1.927729 | 3.444269 | 0.096359 |
|  |  |  |  |  |  |  |
|  |  |  |  |  |  |  |
| **VX 12** | 4.42 | -1379.87183 | 15 | -2.410110 | -0.720491 | 0.278344 |
|  |  |  | 8 | -1.556502 | -1.459244 | -0.695600 |
|  |  |  | 16 | -1.279230 | 0.575103 | 1.543890 |
|  |  |  | 8 | -3.543630 | 0.253692 | -0.347395 |
|  |  |  | 6 | -3.447171 | -1.755258 | 1.343306 |
|  |  |  | 1 | -4.107650 | -2.350440 | 0.709844 |
|  |  |  | 1 | -2.799993 | -2.417129 | 1.919896 |
|  |  |  | 1 | -4.036425 | -1.136719 | 2.020444 |
|  |  |  | 6 | -3.228544 | 1.179349 | -1.420794 |
|  |  |  | 6 | -4.496512 | 1.926903 | -1.778075 |
|  |  |  | 1 | -2.843581 | 0.608050 | -2.269021 |
|  |  |  | 1 | -2.446743 | 1.861645 | -1.074285 |
|  |  |  | 1 | -4.292601 | 2.630598 | -2.590015 |
|  |  |  | 1 | -5.274660 | 1.235561 | -2.108587 |
|  |  |  | 1 | -4.871531 | 2.489179 | -0.920215 |
|  |  |  | 6 | 0.425605 | -0.135559 | 1.344296 |
|  |  |  | 6 | 1.169019 | 0.410997 | 0.118987 |
|  |  |  | 1 | 0.354492 | -1.220604 | 1.309392 |
|  |  |  | 1 | 0.939640 | 0.142619 | 2.264748 |
|  |  |  | 1 | 0.620763 | 0.125991 | -0.782642 |
|  |  |  | 1 | 1.144125 | 1.503315 | 0.157794 |
|  |  |  | 7 | 2.552932 | -0.079920 | 0.073336 |
|  |  |  | 6 | 3.572064 | 0.976481 | 0.255895 |
|  |  |  | 6 | 3.433590 | 1.653745 | 1.625793 |
|  |  |  | 6 | 3.628131 | 2.019735 | -0.879269 |
|  |  |  | 1 | 4.534121 | 0.463055 | 0.275418 |
|  |  |  | 1 | 3.444746 | 0.908343 | 2.424467 |
|  |  |  | 1 | 4.268924 | 2.340983 | 1.787205 |
|  |  |  | 1 | 2.513334 | 2.238648 | 1.712961 |
|  |  |  | 1 | 3.769662 | 1.543732 | -1.853213 |
|  |  |  | 1 | 2.713209 | 2.617687 | -0.928578 |
|  |  |  | 1 | 4.462386 | 2.710075 | -0.722036 |
|  |  |  | 6 | 2.794054 | -1.039841 | -1.031590 |
|  |  |  | 6 | 1.894886 | -2.275677 | -0.879177 |
|  |  |  | 6 | 4.256164 | -1.497885 | -1.106251 |
|  |  |  | 1 | 2.542862 | -0.566498 | -1.999477 |
|  |  |  | 1 | 0.831476 | -2.033975 | -0.866986 |
|  |  |  | 1 | 2.061989 | -2.954847 | -1.720206 |
|  |  |  | 1 | 2.143041 | -2.809372 | 0.044181 |
|  |  |  | 1 | 4.943068 | -0.704045 | -1.405679 |
|  |  |  | 1 | 4.586119 | -1.898824 | -0.142638 |
|  |  |  | 1 | 4.341374 | -2.295402 | -1.848293 |
|  |  |  |  |  |  |  |
|  |  |  |  |  |  |  |
| **VX 13** | 4.51 | -1379.87185 | 15 | -2.377956 | 0.118294 | -0.755321 |
|  |  |  | 8 | -1.546988 | 1.356043 | -0.772134 |
|  |  |  | 16 | -1.237125 | -1.642153 | -0.359367 |
|  |  |  | 8 | -3.556785 | 0.051957 | 0.354517 |
|  |  |  | 6 | -3.348724 | -0.180677 | -2.254988 |
|  |  |  | 1 | -4.016248 | 0.669258 | -2.409424 |
|  |  |  | 1 | -2.665138 | -0.265282 | -3.100615 |
|  |  |  | 1 | -3.928483 | -1.098689 | -2.158405 |
|  |  |  | 6 | -3.305766 | 0.400829 | 1.741490 |
|  |  |  | 6 | -4.618622 | 0.303330 | 2.490488 |
|  |  |  | 1 | -2.895533 | 1.413061 | 1.776042 |
|  |  |  | 1 | -2.562575 | -0.293047 | 2.145065 |
|  |  |  | 1 | -4.462894 | 0.561223 | 3.541810 |
|  |  |  | 1 | -5.356336 | 0.991511 | 2.072326 |
|  |  |  | 1 | -5.020653 | -0.710908 | 2.440358 |
|  |  |  | 6 | 0.469001 | -1.076524 | -0.826981 |
|  |  |  | 6 | 1.175659 | -0.290780 | 0.285041 |
|  |  |  | 1 | 0.409467 | -0.485703 | -1.739276 |
|  |  |  | 1 | 0.999120 | -2.000595 | -1.057366 |
|  |  |  | 1 | 0.617092 | 0.631167 | 0.449884 |
|  |  |  | 1 | 1.118036 | -0.855983 | 1.222189 |
|  |  |  | 7 | 2.571640 | -0.001602 | -0.069952 |
|  |  |  | 6 | 2.855233 | 1.442559 | -0.247763 |
|  |  |  | 6 | 2.717539 | 2.282749 | 1.038003 |
|  |  |  | 6 | 2.019248 | 2.031606 | -1.391230 |
|  |  |  | 1 | 3.895431 | 1.504117 | -0.570002 |
|  |  |  | 1 | 3.338252 | 1.886487 | 1.846706 |
|  |  |  | 1 | 3.033609 | 3.313515 | 0.852024 |
|  |  |  | 1 | 1.683055 | 2.317522 | 1.391354 |
|  |  |  | 1 | 2.188882 | 1.469002 | -2.313042 |
|  |  |  | 1 | 0.947514 | 2.029782 | -1.176960 |
|  |  |  | 1 | 2.317444 | 3.069310 | -1.567640 |
|  |  |  | 6 | 3.546951 | -0.768115 | 0.737048 |
|  |  |  | 6 | 5.002322 | -0.417571 | 0.402510 |
|  |  |  | 6 | 3.351170 | -2.278698 | 0.532649 |
|  |  |  | 1 | 3.393338 | -0.560519 | 1.813340 |
|  |  |  | 1 | 5.279634 | 0.593208 | 0.705657 |
|  |  |  | 1 | 5.667088 | -1.106475 | 0.929599 |
|  |  |  | 1 | 5.188988 | -0.522501 | -0.670697 |
|  |  |  | 1 | 2.349189 | -2.615196 | 0.804193 |
|  |  |  | 1 | 3.532559 | -2.547207 | -0.512517 |
|  |  |  | 1 | 4.057131 | -2.832044 | 1.158072 |
|  |  |  |  |  |  |  |
|  |  |  |  |  |  |  |
| **VX 14** | 4.59 | -1379.87169 | 15 | 2.236171 | 0.410122 | 0.547353 |
|  |  |  | 8 | 1.556769 | 1.440152 | -0.289885 |
|  |  |  | 16 | 1.207410 | -1.463575 | 0.510311 |
|  |  |  | 8 | 3.760896 | 0.047012 | 0.134180 |
|  |  |  | 6 | 2.498082 | 0.846955 | 2.285221 |
|  |  |  | 1 | 3.141140 | 1.728134 | 2.326676 |
|  |  |  | 1 | 1.532727 | 1.074726 | 2.737766 |
|  |  |  | 1 | 2.967899 | 0.020679 | 2.819721 |
|  |  |  | 6 | 4.115930 | -0.245419 | -1.242582 |
|  |  |  | 6 | 5.613894 | -0.462442 | -1.301911 |
|  |  |  | 1 | 3.805689 | 0.595377 | -1.867918 |
|  |  |  | 1 | 3.570159 | -1.139424 | -1.559297 |
|  |  |  | 1 | 5.913358 | -0.683720 | -2.330213 |
|  |  |  | 1 | 6.148659 | 0.430811 | -0.971992 |
|  |  |  | 1 | 5.911276 | -1.300782 | -0.668248 |
|  |  |  | 6 | -0.329476 | -0.991013 | -0.416491 |
|  |  |  | 6 | -1.377253 | -0.285719 | 0.452253 |
|  |  |  | 1 | -0.707805 | -1.938112 | -0.801415 |
|  |  |  | 1 | -0.036944 | -0.365260 | -1.256957 |
|  |  |  | 1 | -1.554307 | -0.871682 | 1.360802 |
|  |  |  | 1 | -0.956589 | 0.666431 | 0.782149 |
|  |  |  | 7 | -2.629834 | -0.068452 | -0.283400 |
|  |  |  | 6 | -2.927142 | 1.359034 | -0.551315 |
|  |  |  | 6 | -1.839617 | 1.993435 | -1.427276 |
|  |  |  | 6 | -3.196626 | 2.201664 | 0.712099 |
|  |  |  | 1 | -3.839357 | 1.365739 | -1.149029 |
|  |  |  | 1 | -1.721709 | 1.426284 | -2.354150 |
|  |  |  | 1 | -2.128374 | 3.015737 | -1.689059 |
|  |  |  | 1 | -0.866424 | 2.042598 | -0.932562 |
|  |  |  | 1 | -3.990558 | 1.765757 | 1.325112 |
|  |  |  | 1 | -2.303401 | 2.297814 | 1.336368 |
|  |  |  | 1 | -3.508632 | 3.212443 | 0.433392 |
|  |  |  | 6 | -3.750449 | -0.891095 | 0.224638 |
|  |  |  | 6 | -3.426854 | -2.387186 | 0.086168 |
|  |  |  | 6 | -5.071964 | -0.610141 | -0.501665 |
|  |  |  | 1 | -3.911495 | -0.687243 | 1.300560 |
|  |  |  | 1 | -2.518741 | -2.673638 | 0.619431 |
|  |  |  | 1 | -4.244976 | -2.983825 | 0.498980 |
|  |  |  | 1 | -3.303891 | -2.652317 | -0.968207 |
|  |  |  | 1 | -5.472863 | 0.382806 | -0.292300 |
|  |  |  | 1 | -4.948379 | -0.714375 | -1.584022 |
|  |  |  | 1 | -5.821225 | -1.336169 | -0.176258 |
|  |  |  |  |  |  |  |
|  |  |  |  |  |  |  |
| **VX 15** | 6.91 | -1379.87034 | 15 | 2.639693 | -0.881993 | 0.258522 |
|  |  |  | 8 | 2.650667 | -0.990253 | 1.745638 |
|  |  |  | 16 | 0.695133 | -0.490491 | -0.534421 |
|  |  |  | 8 | 3.538014 | 0.303963 | -0.387274 |
|  |  |  | 6 | 3.337835 | -2.302161 | -0.624369 |
|  |  |  | 1 | 4.377083 | -2.425638 | -0.313677 |
|  |  |  | 1 | 2.769268 | -3.193124 | -0.355007 |
|  |  |  | 1 | 3.286693 | -2.145438 | -1.701958 |
|  |  |  | 6 | 3.485064 | 1.655995 | 0.137691 |
|  |  |  | 6 | 4.447025 | 2.505679 | -0.666946 |
|  |  |  | 1 | 3.758079 | 1.628285 | 1.195453 |
|  |  |  | 1 | 2.458786 | 2.025001 | 0.050868 |
|  |  |  | 1 | 4.432366 | 3.533376 | -0.293093 |
|  |  |  | 1 | 5.466546 | 2.123128 | -0.583215 |
|  |  |  | 1 | 4.165539 | 2.518533 | -1.722155 |
|  |  |  | 6 | -0.348792 | -1.093102 | 0.876736 |
|  |  |  | 6 | -1.518345 | -0.142913 | 1.137170 |
|  |  |  | 1 | 0.292853 | -1.147835 | 1.757157 |
|  |  |  | 1 | -0.687160 | -2.099879 | 0.630860 |
|  |  |  | 1 | -2.067189 | -0.542747 | 2.010109 |
|  |  |  | 1 | -1.102943 | 0.816098 | 1.450217 |
|  |  |  | 7 | -2.376159 | 0.081419 | -0.019217 |
|  |  |  | 6 | -2.992405 | 1.421562 | -0.111547 |
|  |  |  | 6 | -1.987400 | 2.440545 | -0.669313 |
|  |  |  | 6 | -3.634790 | 1.952026 | 1.188171 |
|  |  |  | 1 | -3.792665 | 1.329497 | -0.850282 |
|  |  |  | 1 | -1.588706 | 2.098914 | -1.626051 |
|  |  |  | 1 | -2.465158 | 3.414770 | -0.812782 |
|  |  |  | 1 | -1.143013 | 2.583222 | 0.011420 |
|  |  |  | 1 | -4.363108 | 1.253270 | 1.603071 |
|  |  |  | 1 | -2.883553 | 2.154245 | 1.957162 |
|  |  |  | 1 | -4.152618 | 2.894767 | 0.987595 |
|  |  |  | 6 | -3.212041 | -1.069504 | -0.407840 |
|  |  |  | 6 | -3.471068 | -1.085813 | -1.920895 |
|  |  |  | 6 | -4.527633 | -1.235614 | 0.381140 |
|  |  |  | 1 | -2.603912 | -1.952852 | -0.192143 |
|  |  |  | 1 | -2.529398 | -1.020174 | -2.469450 |
|  |  |  | 1 | -3.978979 | -2.011000 | -2.208809 |
|  |  |  | 1 | -4.108862 | -0.255211 | -2.237059 |
|  |  |  | 1 | -4.354569 | -1.236788 | 1.460663 |
|  |  |  | 1 | -5.239739 | -0.438521 | 0.149347 |
|  |  |  | 1 | -5.004192 | -2.185961 | 0.122268 |
|  |  |  |  |  |  |  |

Table S10: Cartesian coordinates (in Å) and total electronic energies (in Hartree) of gas-phase optimised geometries of A234, GB, VR and VX, obtained using the M062X/6-311++G(d,p) method.

| Species | Total Electronic  Energy (Hartree) | Cartesian Coordinates (in Å) | | | |
| --- | --- | --- | --- | --- | --- |
| A234 | -1016.84042 | 7 | -2.281926 | 0.307635 | -0.077647 |
|  |  | 6 | -1.164711 | -0.429103 | -0.227027 |
|  |  | 7 | -0.091843 | -0.021280 | 0.386430 |
|  |  | 15 | 1.382745 | -0.687228 | 0.380890 |
|  |  | 8 | 1.674756 | -2.001162 | -0.219950 |
|  |  | 9 | 1.749421 | -0.701557 | 1.926907 |
|  |  | 8 | 2.312584 | 0.498119 | -0.144500 |
|  |  | 6 | -2.247335 | 1.505075 | 0.768086 |
|  |  | 6 | -1.687342 | 2.710094 | 0.021180 |
|  |  | 1 | -1.636654 | 1.283651 | 1.642498 |
|  |  | 1 | -3.271086 | 1.691998 | 1.100643 |
|  |  | 1 | -1.699258 | 3.592201 | 0.664358 |
|  |  | 1 | -0.657342 | 2.510834 | -0.276698 |
|  |  | 1 | -2.280579 | 2.930060 | -0.870053 |
|  |  | 6 | -3.547139 | 0.019761 | -0.748152 |
|  |  | 6 | -4.507328 | -0.774865 | 0.132530 |
|  |  | 1 | -3.356274 | -0.504126 | -1.683209 |
|  |  | 1 | -3.991255 | 0.981932 | -1.017622 |
|  |  | 1 | -5.460260 | -0.924084 | -0.378841 |
|  |  | 1 | -4.089595 | -1.753043 | 0.378468 |
|  |  | 1 | -4.701089 | -0.247333 | 1.068547 |
|  |  | 6 | -1.251597 | -1.654472 | -1.108107 |
|  |  | 1 | -2.155033 | -2.226641 | -0.897468 |
|  |  | 1 | -1.279191 | -1.344986 | -2.156492 |
|  |  | 1 | -0.376617 | -2.281605 | -0.958434 |
|  |  | 6 | 3.717172 | 0.236576 | -0.329901 |
|  |  | 6 | 4.317590 | 1.439846 | -1.019538 |
|  |  | 1 | 3.829956 | -0.672910 | -0.924819 |
|  |  | 1 | 4.172538 | 0.071729 | 0.651086 |
|  |  | 1 | 5.387662 | 1.286224 | -1.172094 |
|  |  | 1 | 3.844976 | 1.594340 | -1.990554 |
|  |  | 1 | 4.174934 | 2.336568 | -0.415128 |
|  |  |  |  |  |  |
|  |  |  |  |  |  |
| GB | -750.12947 | 6 | 1.610306 | 0.103072 | 0.305376 |
|  |  | 1 | 1.468642 | 0.071724 | 1.389110 |
|  |  | 6 | 2.673005 | 1.113781 | -0.068226 |
|  |  | 6 | 1.897509 | -1.291626 | -0.220734 |
|  |  | 1 | 1.124733 | -1.996801 | 0.091492 |
|  |  | 1 | 1.946373 | -1.278859 | -1.311865 |
|  |  | 1 | 2.854716 | -1.642819 | 0.170161 |
|  |  | 1 | 2.786028 | 1.156458 | -1.153625 |
|  |  | 1 | 3.629251 | 0.824233 | 0.371695 |
|  |  | 1 | 2.405465 | 2.105896 | 0.296558 |
|  |  | 8 | 0.368324 | 0.601095 | -0.272282 |
|  |  | 8 | -1.092146 | -0.625249 | 1.484258 |
|  |  | 15 | -1.028748 | -0.029880 | 0.145503 |
|  |  | 6 | -2.211816 | 1.233031 | -0.290126 |
|  |  | 1 | -2.106081 | 2.063868 | 0.406435 |
|  |  | 1 | -2.032791 | 1.576442 | -1.308287 |
|  |  | 1 | -3.215411 | 0.816426 | -0.207468 |
|  |  | 9 | -1.272572 | -1.111631 | -0.997811 |
|  |  |  |  |  |  |
|  |  |  |  |  |  |
| VR | -1379.50138 | 15 | 1.001731 | -0.261258 | 1.063468 |
|  |  | 8 | 0.246289 | 0.998968 | 1.261646 |
|  |  | 16 | 0.401137 | -1.245857 | -0.710670 |
|  |  | 8 | 2.592750 | -0.116964 | 0.896982 |
|  |  | 6 | 0.927505 | -1.448618 | 2.410850 |
|  |  | 1 | 1.358766 | -0.987926 | 3.300506 |
|  |  | 1 | -0.117183 | -1.699861 | 2.592985 |
|  |  | 1 | 1.483398 | -2.348181 | 2.147539 |
|  |  | 6 | 3.123455 | 0.853639 | -0.024010 |
|  |  | 1 | 3.102481 | 1.832137 | 0.462087 |
|  |  | 1 | 2.489089 | 0.890507 | -0.918508 |
|  |  | 6 | -1.124082 | -0.278328 | -1.024125 |
|  |  | 6 | -2.288899 | -0.696856 | -0.133815 |
|  |  | 1 | -1.369960 | -0.448319 | -2.072751 |
|  |  | 1 | -0.883498 | 0.774284 | -0.884293 |
|  |  | 1 | -2.599082 | -1.706739 | -0.409563 |
|  |  | 1 | -1.965177 | -0.734006 | 0.919796 |
|  |  | 7 | -3.397960 | 0.231016 | -0.306108 |
|  |  | 6 | -3.234201 | 1.404609 | 0.559498 |
|  |  | 6 | -3.918173 | 2.641213 | -0.007474 |
|  |  | 1 | -2.164922 | 1.600524 | 0.674947 |
|  |  | 1 | -3.614527 | 1.183221 | 1.571868 |
|  |  | 1 | -3.777164 | 3.493327 | 0.660969 |
|  |  | 1 | -3.492840 | 2.886745 | -0.982217 |
|  |  | 1 | -4.992610 | 2.492825 | -0.136162 |
|  |  | 6 | -4.696162 | -0.412660 | -0.117677 |
|  |  | 6 | -5.108701 | -1.256817 | -1.317081 |
|  |  | 1 | -5.439976 | 0.373293 | 0.028866 |
|  |  | 1 | -4.700193 | -1.022542 | 0.803344 |
|  |  | 1 | -6.093259 | -1.700514 | -1.153146 |
|  |  | 1 | -5.147945 | -0.633839 | -2.212312 |
|  |  | 1 | -4.406415 | -2.071991 | -1.503324 |
|  |  | 6 | 4.534123 | 0.441536 | -0.404130 |
|  |  | 6 | 5.145183 | 1.517377 | -1.301081 |
|  |  | 6 | 4.531494 | -0.921603 | -1.091370 |
|  |  | 1 | 5.117398 | 0.375634 | 0.521130 |
|  |  | 1 | 5.157907 | 2.493305 | -0.809696 |
|  |  | 1 | 6.172504 | 1.258914 | -1.565203 |
|  |  | 1 | 4.576557 | 1.612002 | -2.231419 |
|  |  | 1 | 4.101562 | -1.687035 | -0.443686 |
|  |  | 1 | 3.937995 | -0.883270 | -2.010552 |
|  |  | 1 | 5.547087 | -1.223999 | -1.356288 |
|  |  |  |  |  |  |
|  |  |  |  |  |  |
| VX | -1379.50089 | 15 | 2.056079 | -0.030006 | 0.664211 |
|  |  | 8 | 1.290907 | 1.224033 | 0.459588 |
|  |  | 16 | 1.326935 | -1.591506 | -0.561552 |
|  |  | 8 | 3.624143 | 0.041794 | 0.315542 |
|  |  | 6 | 2.134702 | -0.643757 | 2.351822 |
|  |  | 1 | 2.623965 | 0.110243 | 2.969768 |
|  |  | 1 | 1.118370 | -0.812920 | 2.707028 |
|  |  | 1 | 2.696951 | -1.576676 | 2.383564 |
|  |  | 6 | 4.038274 | 0.638019 | -0.929029 |
|  |  | 6 | 5.542620 | 0.522288 | -1.013703 |
|  |  | 1 | 3.707243 | 1.679041 | -0.940513 |
|  |  | 1 | 3.550479 | 0.104741 | -1.750964 |
|  |  | 1 | 5.897702 | 0.966142 | -1.945715 |
|  |  | 1 | 6.012947 | 1.042202 | -0.178120 |
|  |  | 1 | 5.845248 | -0.525461 | -0.988957 |
|  |  | 6 | -0.232742 | -0.779749 | -1.087984 |
|  |  | 6 | -1.300483 | -0.769605 | 0.004513 |
|  |  | 1 | -0.580231 | -1.346264 | -1.952965 |
|  |  | 1 | 0.012150 | 0.232120 | -1.405174 |
|  |  | 1 | -1.634896 | -1.795998 | 0.168502 |
|  |  | 1 | -0.857514 | -0.421654 | 0.950716 |
|  |  | 7 | -2.412960 | 0.064070 | -0.408635 |
|  |  | 6 | -2.184213 | 1.495092 | -0.153462 |
|  |  | 6 | -2.540610 | 1.959355 | 1.263837 |
|  |  | 6 | -2.887408 | 2.351595 | -1.204613 |
|  |  | 1 | -1.106233 | 1.644978 | -0.269223 |
|  |  | 1 | -2.026580 | 1.359755 | 2.019159 |
|  |  | 1 | -2.231608 | 2.998019 | 1.401392 |
|  |  | 1 | -3.618291 | 1.903017 | 1.444305 |
|  |  | 1 | -2.591814 | 2.035564 | -2.206516 |
|  |  | 1 | -3.975854 | 2.275259 | -1.128432 |
|  |  | 1 | -2.623775 | 3.403848 | -1.074318 |
|  |  | 6 | -3.752794 | -0.463375 | -0.130558 |
|  |  | 6 | -3.958375 | -1.040526 | 1.279912 |
|  |  | 6 | -4.123393 | -1.505617 | -1.186101 |
|  |  | 1 | -4.439471 | 0.380077 | -0.243913 |
|  |  | 1 | -3.627409 | -0.350221 | 2.055953 |
|  |  | 1 | -5.018185 | -1.252480 | 1.442748 |
|  |  | 1 | -3.417862 | -1.982167 | 1.406654 |
|  |  | 1 | -4.068263 | -1.067672 | -2.183772 |
|  |  | 1 | -3.430806 | -2.352517 | -1.147991 |
|  |  | 1 | -5.131554 | -1.894197 | -1.019958 |

Table S11: Cartesian coordinates (in Å) and total electronic energies (in Hartree) of gas-phase optimised geometries depicted in Figure S6a obtained using the M062X/6-311++G(d,p) method.

| Species | Total Electronic  Energy (Hartree) | Cartesian Coordinates (in Å) | | | |
| --- | --- | --- | --- | --- | --- |
| A234-OMe_1 | -1032.0997 | 7 | 2.36839 | 0.01623 | -0.1935 |
|  |  | 6 | 1.15406 | -0.4798 | 0.13028 |
|  |  | 7 | 0.20724 | 0.36178 | 0.41359 |
|  |  | 15 | -1.3237 | -0.0507 | 0.83038 |
|  |  | 8 | -1.5863 | -1.093 | 1.84035 |
|  |  | 8 | -1.995 | -0.3718 | -0.6092 |
|  |  | 6 | 2.57293 | 1.46537 | -0.1873 |
|  |  | 6 | 2.10179 | 2.1108 | -1.4865 |
|  |  | 1 | 2.03041 | 1.87964 | 0.66179 |
|  |  | 1 | 3.6398 | 1.6412 | -0.0301 |
|  |  | 1 | 2.27546 | 3.18859 | -1.4618 |
|  |  | 1 | 1.03458 | 1.9292 | -1.62 |
|  |  | 1 | 2.63851 | 1.69895 | -2.3447 |
|  |  | 6 | 3.49829 | -0.8006 | -0.6264 |
|  |  | 6 | 4.45602 | -1.1292 | 0.51554 |
|  |  | 1 | 3.13341 | -1.7105 | -1.1007 |
|  |  | 1 | 4.0207 | -0.2378 | -1.405 |
|  |  | 1 | 5.31048 | -1.7001 | 0.14673 |
|  |  | 1 | 3.9529 | -1.7157 | 1.28662 |
|  |  | 1 | 4.83049 | -0.2151 | 0.98045 |
|  |  | 6 | 0.98707 | -1.9828 | 0.11507 |
|  |  | 1 | 1.86618 | -2.4939 | 0.50539 |
|  |  | 1 | 0.81819 | -2.316 | -0.9127 |
|  |  | 1 | 0.12481 | -2.258 | 0.71919 |
|  |  | 6 | -3.3698 | -0.7891 | -0.6224 |
|  |  | 6 | -3.7276 | -1.1583 | -2.0448 |
|  |  | 1 | -3.4884 | -1.6355 | 0.05879 |
|  |  | 1 | -3.9925 | 0.03439 | -0.2571 |
|  |  | 1 | -4.7714 | -1.4736 | -2.1 |
|  |  | 1 | -3.0969 | -1.9775 | -2.393 |
|  |  | 1 | -3.585 | -0.3041 | -2.7087 |
|  |  | 8 | -1.987 | 1.34496 | 1.2608 |
|  |  | 6 | -1.8333 | 2.49322 | 0.4318 |
|  |  | 1 | -2.4288 | 3.28456 | 0.88264 |
|  |  | 1 | -0.7832 | 2.78862 | 0.38872 |
|  |  | 1 | -2.1949 | 2.28807 | -0.5797 |
|  |  |  |  |  |  |
|  |  |  |  |  |  |
| A234-ONCH_2__1 | -1086.1785 | 7 | 2.51956 | 0.04462 | 0.30313 |
|  |  | 6 | 1.43484 | -0.5651 | -0.2202 |
|  |  | 7 | 0.29465 | 0.04542 | -0.1107 |
|  |  | 15 | -1.1627 | -0.4303 | -0.645 |
|  |  | 8 | -1.3336 | -1.5123 | -1.6343 |
|  |  | 8 | -1.976 | -0.6316 | 0.71869 |
|  |  | 6 | 2.36411 | 1.35047 | 0.95001 |
|  |  | 6 | 1.87938 | 1.21558 | 2.38941 |
|  |  | 1 | 1.65301 | 1.9352 | 0.36754 |
|  |  | 1 | 3.33657 | 1.84699 | 0.90906 |
|  |  | 1 | 1.80713 | 2.19895 | 2.85817 |
|  |  | 1 | 0.89273 | 0.75132 | 2.40071 |
|  |  | 1 | 2.56913 | 0.60601 | 2.97873 |
|  |  | 6 | 3.85563 | -0.5422 | 0.32867 |
|  |  | 6 | 4.72989 | -0.0576 | -0.8249 |
|  |  | 1 | 3.78047 | -1.6284 | 0.32523 |
|  |  | 1 | 4.30836 | -0.2674 | 1.28537 |
|  |  | 1 | 5.73438 | -0.4783 | -0.7472 |
|  |  | 1 | 4.30302 | -0.3542 | -1.7849 |
|  |  | 1 | 4.81344 | 1.03099 | -0.816 |
|  |  | 6 | 1.64129 | -1.9135 | -0.8725 |
|  |  | 1 | 2.5261 | -1.9135 | -1.5091 |
|  |  | 1 | 1.77724 | -2.6737 | -0.0984 |
|  |  | 1 | 0.76636 | -2.1725 | -1.4635 |
|  |  | 6 | -3.3806 | -0.9248 | 0.63266 |
|  |  | 6 | -3.8478 | -1.3352 | 2.01085 |
|  |  | 1 | -3.5337 | -1.722 | -0.0997 |
|  |  | 1 | -3.9011 | -0.0262 | 0.28806 |
|  |  | 1 | -4.9179 | -1.551 | 1.99477 |
|  |  | 1 | -3.3154 | -2.2284 | 2.34011 |
|  |  | 1 | -3.6618 | -0.534 | 2.72732 |
|  |  | 8 | -1.8281 | 0.92392 | -1.2999 |
|  |  | 7 | -1.9028 | 2.02689 | -0.4474 |
|  |  | 6 | -2.3119 | 3.04113 | -1.081 |
|  |  | 1 | -2.5459 | 3.00874 | -2.1428 |
|  |  | 1 | -2.4197 | 3.95401 | -0.508 |
|  |  |  |  |  |  |
|  |  |  |  |  |  |
| A234-ONH_2__1 | -1048.09415 | 7 | 2.36382 | 0.46337 | 0.02931 |
|  |  | 6 | 1.25513 | -0.2274 | 0.3788 |
|  |  | 7 | 0.11738 | 0.19223 | -0.0852 |
|  |  | 15 | -1.3432 | -0.4825 | 0.16154 |
|  |  | 8 | -1.6272 | -1.2584 | 1.38973 |
|  |  | 8 | -2.3325 | 0.74207 | -0.0959 |
|  |  | 6 | 2.23904 | 1.62411 | -0.8548 |
|  |  | 6 | 1.85282 | 2.88369 | -0.0872 |
|  |  | 1 | 1.48617 | 1.39658 | -1.6081 |
|  |  | 1 | 3.20151 | 1.75286 | -1.356 |
|  |  | 1 | 1.80021 | 3.73939 | -0.7633 |
|  |  | 1 | 0.87516 | 2.74416 | 0.37474 |
|  |  | 1 | 2.58664 | 3.10757 | 0.69121 |
|  |  | 6 | 3.70448 | 0.16248 | 0.52064 |
|  |  | 6 | 4.50657 | -0.6955 | -0.4548 |
|  |  | 1 | 3.64067 | -0.3203 | 1.49424 |
|  |  | 1 | 4.20996 | 1.11854 | 0.68309 |
|  |  | 1 | 5.5169 | -0.8633 | -0.0763 |
|  |  | 1 | 4.02569 | -1.6644 | -0.6024 |
|  |  | 1 | 4.58317 | -0.2061 | -1.4278 |
|  |  | 6 | 1.44749 | -1.4171 | 1.29247 |
|  |  | 1 | 2.27692 | -2.0379 | 0.95292 |
|  |  | 1 | 1.6683 | -1.0668 | 2.3043 |
|  |  | 1 | 0.53884 | -2.0091 | 1.33335 |
|  |  | 6 | -3.7302 | 0.56127 | 0.18913 |
|  |  | 6 | -4.4044 | 1.90489 | 0.02779 |
|  |  | 1 | -3.8359 | 0.17259 | 1.20466 |
|  |  | 1 | -4.1379 | -0.1744 | -0.5102 |
|  |  | 1 | -5.4734 | 1.81343 | 0.23052 |
|  |  | 1 | -3.9764 | 2.62801 | 0.72333 |
|  |  | 1 | -4.2708 | 2.27805 | -0.9884 |
|  |  | 8 | -1.6757 | -1.3537 | -1.1907 |
|  |  | 7 | -0.8504 | -2.5212 | -1.2093 |
|  |  | 1 | -0.6566 | -2.6501 | -2.1982 |
|  |  | 1 | -1.4689 | -3.2791 | -0.9268 |
|  |  |  |  |  |  |
|  |  |  |  |  |  |
| A234-OMe_2 | -786.04576 | 15 | 0.56686 | -0.5282 | -0.1129 |
|  |  | 8 | 0.48006 | -1.8182 | -0.7802 |
|  |  | 9 | 0.90587 | -0.6532 | 1.43081 |
|  |  | 8 | -0.7374 | 0.3713 | -0.0777 |
|  |  | 6 | -2.0293 | -0.244 | 0.14535 |
|  |  | 6 | -3.0819 | 0.81217 | -0.0906 |
|  |  | 1 | -2.1317 | -1.0855 | -0.5425 |
|  |  | 1 | -2.0568 | -0.6183 | 1.1719 |
|  |  | 1 | -4.0735 | 0.38623 | 0.07175 |
|  |  | 1 | -3.0243 | 1.18253 | -1.1146 |
|  |  | 1 | -2.946 | 1.6489 | 0.59556 |
|  |  | 8 | 1.67528 | 0.46164 | -0.6216 |
|  |  | 6 | 1.85896 | 1.75717 | -0.029 |
|  |  | 1 | 2.09831 | 1.65182 | 1.02992 |
|  |  | 1 | 2.69227 | 2.21252 | -0.5564 |
|  |  | 1 | 0.95576 | 2.35369 | -0.1577 |
|  |  |  |  |  |  |
|  |  |  |  |  |  |
| A234-ONCH_2__2 | -840.122278 | 15 | -0.1195 | -0.7689 | -0.0972 |
|  |  | 8 | 0.23724 | -1.9373 | -0.8877 |
|  |  | 9 | -0.3358 | -1.0868 | 1.42819 |
|  |  | 8 | 0.88147 | 0.44616 | -0.065 |
|  |  | 6 | 2.30107 | 0.20758 | 0.10426 |
|  |  | 6 | 3.00681 | 1.52314 | -0.1185 |
|  |  | 1 | 2.61095 | -0.5517 | -0.6167 |
|  |  | 1 | 2.46448 | -0.1708 | 1.11641 |
|  |  | 1 | 4.0825 | 1.38952 | 0.00869 |
|  |  | 1 | 2.8161 | 1.88908 | -1.1277 |
|  |  | 1 | 2.65897 | 2.26773 | 0.59798 |
|  |  | 8 | -1.5114 | -0.1015 | -0.52 |
|  |  | 7 | -1.9338 | 0.97485 | 0.29846 |
|  |  | 6 | -3.0104 | 1.44846 | -0.1574 |
|  |  | 1 | -3.4944 | 1.05471 | -1.0475 |
|  |  | 1 | -3.4312 | 2.27923 | 0.39568 |
|  |  |  |  |  |  |
|  |  |  |  |  |  |
| A234-ONH_2__2 | -802.039552 | 15 | 0.62728 | -0.3731 | 0.14272 |
|  |  | 8 | 0.36353 | -0.5152 | 1.57072 |
|  |  | 9 | 1.63765 | -1.4113 | -0.4457 |
|  |  | 8 | -0.6133 | -0.5062 | -0.8179 |
|  |  | 6 | -1.9449 | -0.7345 | -0.2872 |
|  |  | 6 | -2.5724 | 0.56905 | 0.15242 |
|  |  | 1 | -1.8757 | -1.4419 | 0.54037 |
|  |  | 1 | -2.4874 | -1.1944 | -1.1113 |
|  |  | 1 | -3.5903 | 0.38458 | 0.50233 |
|  |  | 1 | -1.9962 | 1.01185 | 0.96614 |
|  |  | 1 | -2.6091 | 1.27529 | -0.6783 |
|  |  | 8 | 1.41165 | 0.93731 | -0.3562 |
|  |  | 7 | 0.62571 | 2.09928 | -0.0577 |
|  |  | 1 | 0.65051 | 2.6241 | -0.9279 |
|  |  | 1 | 1.18953 | 2.60848 | 0.61878 |
|  |  |  |  |  |  |
|  |  |  |  |  |  |
| A234-OMe_3 | -977.531927 | 7 | 2.00827 | 0.18369 | -0.0561 |
|  |  | 6 | 0.82828 | -0.4533 | -0.1751 |
|  |  | 7 | -0.2317 | 0.16287 | 0.26249 |
|  |  | 15 | -1.7678 | -0.3391 | 0.23359 |
|  |  | 8 | -2.1772 | -1.6752 | -0.2349 |
|  |  | 9 | -2.2247 | -0.1266 | 1.74063 |
|  |  | 6 | 2.05063 | 1.51433 | 0.55881 |
|  |  | 6 | 1.68995 | 2.60884 | -0.4393 |
|  |  | 1 | 1.3564 | 1.52243 | 1.39778 |
|  |  | 1 | 3.06195 | 1.65586 | 0.9466 |
|  |  | 1 | 1.76101 | 3.59005 | 0.03376 |
|  |  | 1 | 0.66715 | 2.46431 | -0.7888 |
|  |  | 1 | 2.36626 | 2.59363 | -1.2976 |
|  |  | 6 | 3.27698 | -0.3454 | -0.5503 |
|  |  | 6 | 4.07918 | -1.0508 | 0.53971 |
|  |  | 1 | 3.09435 | -1.0142 | -1.3897 |
|  |  | 1 | 3.84288 | 0.50155 | -0.9473 |
|  |  | 1 | 5.03946 | -1.3907 | 0.14725 |
|  |  | 1 | 3.53542 | -1.9163 | 0.92273 |
|  |  | 1 | 4.27137 | -0.3768 | 1.37681 |
|  |  | 6 | 0.83752 | -1.8207 | -0.8197 |
|  |  | 1 | 1.6461 | -2.4351 | -0.4236 |
|  |  | 1 | 0.98698 | -1.7124 | -1.8973 |
|  |  | 1 | -0.1155 | -2.3163 | -0.6556 |
|  |  | 8 | -2.5252 | 0.86625 | -0.4874 |
|  |  | 6 | -3.9253 | 0.7177 | -0.7489 |
|  |  | 1 | -4.2215 | 1.58564 | -1.3327 |
|  |  | 1 | -4.1104 | -0.1997 | -1.3091 |
|  |  | 1 | -4.4832 | 0.69684 | 0.18971 |
|  |  |  |  |  |  |
|  |  |  |  |  |  |
| A234-ONCH_2__3 | -1031.60942 | 7 | -2.038 | 0.32634 | -0.0239 |
|  |  | 6 | -1.0121 | -0.5227 | -0.2141 |
|  |  | 7 | 0.14332 | -0.1836 | 0.28162 |
|  |  | 15 | 1.55182 | -0.9687 | 0.19876 |
|  |  | 8 | 1.6946 | -2.3149 | -0.3646 |
|  |  | 9 | 2.06842 | -0.8762 | 1.68704 |
|  |  | 8 | 2.59528 | 0.03627 | -0.5419 |
|  |  | 6 | -1.8197 | 1.56029 | 0.73912 |
|  |  | 6 | -1.2049 | 2.65844 | -0.1212 |
|  |  | 1 | -1.1653 | 1.32866 | 1.57849 |
|  |  | 1 | -2.7911 | 1.86808 | 1.13238 |
|  |  | 1 | -1.1014 | 3.57695 | 0.45965 |
|  |  | 1 | -0.2151 | 2.34825 | -0.4575 |
|  |  | 1 | -1.8322 | 2.87183 | -0.9903 |
|  |  | 6 | -3.3788 | 0.12324 | -0.5673 |
|  |  | 6 | -4.3277 | -0.5132 | 0.44445 |
|  |  | 1 | -3.3205 | -0.4774 | -1.4733 |
|  |  | 1 | -3.7522 | 1.10509 | -0.8701 |
|  |  | 1 | -5.3296 | -0.6066 | 0.02122 |
|  |  | 1 | -3.9787 | -1.5069 | 0.73152 |
|  |  | 1 | -4.3941 | 0.09442 | 1.34897 |
|  |  | 6 | -1.2839 | -1.7853 | -0.999 |
|  |  | 1 | -2.2073 | -2.2615 | -0.6695 |
|  |  | 1 | -1.3859 | -1.5383 | -2.0592 |
|  |  | 1 | -0.4532 | -2.4772 | -0.8882 |
|  |  | 7 | 2.62343 | 1.35251 | -0.0586 |
|  |  | 6 | 3.50561 | 2.01594 | -0.6724 |
|  |  | 1 | 4.12721 | 1.58075 | -1.4513 |
|  |  | 1 | 3.61456 | 3.05233 | -0.377 |
|  |  |  |  |  |  |
|  |  |  |  |  |  |
| A234-ONH_2__3 | -993.523997 | 7 | -1.9976 | 0.19 | -0.0496 |
|  |  | 6 | -0.8266 | -0.4619 | -0.1733 |
|  |  | 7 | 0.24426 | 0.1466 | 0.25049 |
|  |  | 15 | 1.77571 | -0.3741 | 0.21069 |
|  |  | 8 | 2.15835 | -1.7082 | -0.273 |
|  |  | 9 | 2.265 | -0.1359 | 1.69427 |
|  |  | 8 | 2.48742 | 0.86 | -0.5597 |
|  |  | 6 | -2.0194 | 1.52596 | 0.55483 |
|  |  | 6 | -1.6561 | 2.608 | -0.4559 |
|  |  | 1 | -1.3169 | 1.533 | 1.38689 |
|  |  | 1 | -3.0253 | 1.68201 | 0.95087 |
|  |  | 1 | -1.7106 | 3.59382 | 0.00978 |
|  |  | 1 | -0.6389 | 2.448 | -0.8148 |
|  |  | 1 | -2.3414 | 2.594 | -1.3071 |
|  |  | 6 | -3.2774 | -0.3293 | -0.5256 |
|  |  | 6 | -4.0756 | -1.0157 | 0.57935 |
|  |  | 1 | -3.1111 | -1.0076 | -1.3606 |
|  |  | 1 | -3.838 | 0.52046 | -0.9242 |
|  |  | 1 | -5.0437 | -1.3485 | 0.20013 |
|  |  | 1 | -3.5375 | -1.8838 | 0.96452 |
|  |  | 1 | -4.2515 | -0.3322 | 1.41219 |
|  |  | 6 | -0.8585 | -1.8348 | -0.8054 |
|  |  | 1 | -1.6694 | -2.4362 | -0.3945 |
|  |  | 1 | -1.0193 | -1.7342 | -1.8821 |
|  |  | 1 | 0.09087 | -2.3394 | -0.6485 |
|  |  | 7 | 3.91328 | 0.70221 | -0.554 |
|  |  | 1 | 4.24413 | 1.65009 | -0.3984 |
|  |  | 1 | 4.14395 | 0.45522 | -1.5135 |

Table S12: Cartesian coordinates (in Å) and total electronic energies (in Hartree) of all structures depicted in Scheme 4 obtained using the M062X/6-311++G(d,p) method.

| Species | Total Electronic  Energy (Hartree) | Cartesian Coordinates (in Å) | | | |
| --- | --- | --- | --- | --- | --- |
| **1** | -1016.84042 | 7 | -2.2819 | 0.30764 | -0.0776 |
|  |  | 6 | -1.1647 | -0.4291 | -0.227 |
|  |  | 7 | -0.0918 | -0.0213 | 0.38643 |
|  |  | 15 | 1.38275 | -0.6872 | 0.38089 |
|  |  | 8 | 1.67476 | -2.0012 | -0.22 |
|  |  | 9 | 1.74942 | -0.7016 | 1.92691 |
|  |  | 8 | 2.31258 | 0.49812 | -0.1445 |
|  |  | 6 | -2.2473 | 1.50508 | 0.76809 |
|  |  | 6 | -1.6873 | 2.71009 | 0.02118 |
|  |  | 1 | -1.6367 | 1.28365 | 1.6425 |
|  |  | 1 | -3.2711 | 1.692 | 1.10064 |
|  |  | 1 | -1.6993 | 3.5922 | 0.66436 |
|  |  | 1 | -0.6573 | 2.51083 | -0.2767 |
|  |  | 1 | -2.2806 | 2.93006 | -0.8701 |
|  |  | 6 | -3.5471 | 0.01976 | -0.7482 |
|  |  | 6 | -4.5073 | -0.7749 | 0.13253 |
|  |  | 1 | -3.3563 | -0.5041 | -1.6832 |
|  |  | 1 | -3.9913 | 0.98193 | -1.0176 |
|  |  | 1 | -5.4603 | -0.9241 | -0.3788 |
|  |  | 1 | -4.0896 | -1.753 | 0.37847 |
|  |  | 1 | -4.7011 | -0.2473 | 1.06855 |
|  |  | 6 | -1.2516 | -1.6545 | -1.1081 |
|  |  | 1 | -2.155 | -2.2266 | -0.8975 |
|  |  | 1 | -1.2792 | -1.345 | -2.1565 |
|  |  | 1 | -0.3766 | -2.2816 | -0.9584 |
|  |  | 6 | 3.71717 | 0.23658 | -0.3299 |
|  |  | 6 | 4.31759 | 1.43985 | -1.0195 |
|  |  | 1 | 3.82996 | -0.6729 | -0.9248 |
|  |  | 1 | 4.17254 | 0.07173 | 0.65109 |
|  |  | 1 | 5.38766 | 1.28622 | -1.1721 |
|  |  | 1 | 3.84498 | 1.59434 | -1.9906 |
|  |  | 1 | 4.17493 | 2.33657 | -0.4151 |
|  |  |  |  |  |  |
|  |  |  |  |  |  |
| **2** | -76.4208889 | 8 | 0 | 0 | 0.1166 |
|  |  | 1 | 0 | 0.76167 | -0.4664 |
|  |  | 1 | 0 | -0.7617 | -0.4664 |
|  |  |  |  |  |  |
|  |  |  |  |  |  |
| **3** | -992.812781 | 7 | -2.2483 | 0.04591 | -0.1535 |
|  |  | 6 | -0.9928 | -0.3519 | 0.14302 |
|  |  | 7 | -0.128 | 0.55678 | 0.48115 |
|  |  | 15 | 1.43032 | 0.23726 | 0.85036 |
|  |  | 8 | 1.76861 | -0.757 | 1.89711 |
|  |  | 8 | 2.07803 | -0.0879 | -0.5868 |
|  |  | 6 | -2.5872 | 1.46835 | -0.0725 |
|  |  | 6 | -2.1978 | 2.21561 | -1.3432 |
|  |  | 1 | -2.0699 | 1.89041 | 0.78792 |
|  |  | 1 | -3.6635 | 1.53375 | 0.10575 |
|  |  | 1 | -2.4904 | 3.26503 | -1.272 |
|  |  | 1 | -1.1175 | 2.16283 | -1.4812 |
|  |  | 1 | -2.6895 | 1.78265 | -2.2181 |
|  |  | 6 | -3.2893 | -0.8428 | -0.6611 |
|  |  | 6 | -4.2192 | -1.3469 | 0.43907 |
|  |  | 1 | -2.8343 | -1.6753 | -1.1966 |
|  |  | 1 | -3.8585 | -0.2769 | -1.4038 |
|  |  | 1 | -5.0073 | -1.9737 | 0.01696 |
|  |  | 1 | -3.6666 | -1.9328 | 1.1761 |
|  |  | 1 | -4.6895 | -0.5103 | 0.95974 |
|  |  | 6 | -0.6842 | -1.829 | 0.03675 |
|  |  | 1 | -1.5245 | -2.4468 | 0.34976 |
|  |  | 1 | -0.4384 | -2.0706 | -1.0011 |
|  |  | 1 | 0.17487 | -2.0676 | 0.66146 |
|  |  | 6 | 3.48596 | -0.36 | -0.6521 |
|  |  | 6 | 3.81618 | -0.7398 | -2.0781 |
|  |  | 1 | 3.72261 | -1.1671 | 0.04699 |
|  |  | 1 | 4.02992 | 0.53938 | -0.3469 |
|  |  | 1 | 4.8835 | -0.9484 | -2.1745 |
|  |  | 1 | 3.25871 | -1.6309 | -2.3711 |
|  |  | 1 | 3.55544 | 0.07349 | -2.7567 |
|  |  | 8 | 2.05402 | 1.68374 | 1.20231 |
|  |  | 1 | 2.40864 | 1.68303 | 2.0966 |
|  |  |  |  |  |  |
|  |  |  |  |  |  |
| **4** | -100.443302 | 9 | 0 | 0 | 0.09178 |
|  |  | 1 | 0 | 0 | -0.826 |
|  |  |  |  |  |  |
|  |  |  |  |  |  |
| **5** | -938.24498 | 7 | 1.70692 | 0.12195 | 0.03076 |
|  |  | 6 | 0.49384 | -0.4386 | 0.19111 |
|  |  | 7 | -0.5448 | 0.27266 | -0.14 |
|  |  | 15 | -2.1076 | -0.1299 | -0.0592 |
|  |  | 8 | -2.5953 | -1.4079 | 0.48802 |
|  |  | 9 | -2.5771 | 0.05061 | -1.5616 |
|  |  | 6 | 1.808 | 1.48138 | -0.5099 |
|  |  | 6 | 1.59568 | 2.53395 | 0.57241 |
|  |  | 1 | 1.06248 | 1.59014 | -1.2963 |
|  |  | 1 | 2.79944 | 1.57575 | -0.9588 |
|  |  | 1 | 1.70353 | 3.53566 | 0.15219 |
|  |  | 1 | 0.59168 | 2.43624 | 0.98684 |
|  |  | 1 | 2.32695 | 2.41966 | 1.37646 |
|  |  | 6 | 2.96174 | -0.5239 | 0.40867 |
|  |  | 6 | 3.63819 | -1.2191 | -0.7697 |
|  |  | 1 | 2.78399 | -1.2263 | 1.22124 |
|  |  | 1 | 3.61363 | 0.25624 | 0.81091 |
|  |  | 1 | 4.59412 | -1.6471 | -0.4616 |
|  |  | 1 | 3.00896 | -2.021 | -1.1603 |
|  |  | 1 | 3.82522 | -0.5126 | -1.5806 |
|  |  | 6 | 0.44419 | -1.8389 | 0.75821 |
|  |  | 1 | 1.17584 | -2.4842 | 0.27199 |
|  |  | 1 | 0.67446 | -1.8057 | 1.82646 |
|  |  | 1 | -0.552 | -2.2565 | 0.63989 |
|  |  | 8 | -2.7747 | 1.15407 | 0.63053 |
|  |  | 1 | -3.6242 | 0.93133 | 1.02564 |
|  |  |  |  |  |  |
|  |  |  |  |  |  |
| **6** | -155.012769 | 6 | -0.0848 | 0.55086 | -1E-05 |
|  |  | 6 | 1.21698 | -0.2225 | 9E-06 |
|  |  | 1 | -0.1421 | 1.19312 | 0.88729 |
|  |  | 1 | -0.1422 | 1.19314 | -0.8873 |
|  |  | 1 | 2.07051 | 0.45812 | 0.00019 |
|  |  | 1 | 1.2746 | -0.8585 | 0.8848 |
|  |  | 1 | 1.27481 | -0.8583 | -0.8849 |
|  |  | 8 | -1.1433 | -0.3958 | -3E-05 |
|  |  | 1 | -1.9824 | 0.06884 | 0.00019 |
|  |  |  |  |  |  |
|  |  |  |  |  |  |
| **7** | -746.757886 | 15 | -0.832 | -0.0375 | -0.1008 |
|  |  | 8 | -1.1706 | -0.9428 | -1.1953 |
|  |  | 9 | -1.0647 | -0.6892 | 1.31503 |
|  |  | 8 | 0.6465 | 0.48782 | 0.01902 |
|  |  | 6 | 1.74075 | -0.4622 | 0.01501 |
|  |  | 6 | 3.02343 | 0.33037 | -0.0547 |
|  |  | 1 | 1.62121 | -1.1234 | -0.8466 |
|  |  | 1 | 1.68167 | -1.0536 | 0.93233 |
|  |  | 1 | 3.87714 | -0.3495 | -0.05 |
|  |  | 1 | 3.05297 | 0.92396 | -0.9688 |
|  |  | 1 | 3.10323 | 0.99975 | 0.80229 |
|  |  | 8 | -1.6596 | 1.30692 | 0.00921 |
|  |  | 1 | -2.3903 | 1.34368 | -0.6171 |
|  |  |  |  |  |  |
|  |  |  |  |  |  |
| **8** | -346.49687 | 7 | 0.01385 | -0.1814 | -0.0308 |
|  |  | 6 | 0.04242 | 1.18757 | -0.0915 |
|  |  | 6 | 1.1692 | -0.9268 | -0.5182 |
|  |  | 6 | 2.26631 | -1.0384 | 0.53649 |
|  |  | 1 | 1.55296 | -0.4185 | -1.4029 |
|  |  | 1 | 0.82 | -1.9185 | -0.8195 |
|  |  | 1 | 3.11015 | -1.6185 | 0.15613 |
|  |  | 1 | 2.61889 | -0.0403 | 0.79859 |
|  |  | 1 | 1.89208 | -1.5305 | 1.43814 |
|  |  | 6 | -1.0663 | -0.9543 | 0.56013 |
|  |  | 6 | -2.0644 | -1.4736 | -0.4742 |
|  |  | 1 | -1.5816 | -0.3601 | 1.31493 |
|  |  | 1 | -0.6158 | -1.7969 | 1.09462 |
|  |  | 1 | -2.8291 | -2.0924 | 0.00119 |
|  |  | 1 | -2.5578 | -0.6438 | -0.9848 |
|  |  | 1 | -1.5569 | -2.0768 | -1.23 |
|  |  | 6 | -1.1783 | 1.91249 | 0.43634 |
|  |  | 1 | -2.104 | 1.50629 | 0.0246 |
|  |  | 1 | -1.2309 | 1.83689 | 1.5252 |
|  |  | 1 | -1.114 | 2.96637 | 0.17142 |
|  |  | 7 | 1.07811 | 1.77632 | -0.5707 |
|  |  | 1 | 0.93882 | 2.78118 | -0.5714 |
|  |  |  |  |  |  |
|  |  |  |  |  |  |
| **9** | -726.879894 | 15 | -0.8425 | -0.0615 | -0.1096 |
|  |  | 8 | -1.1675 | -0.9846 | -1.195 |
|  |  | 9 | -0.9625 | -0.7489 | 1.31566 |
|  |  | 8 | 0.64085 | 0.50224 | -0.013 |
|  |  | 6 | 1.74486 | -0.4287 | 0.06825 |
|  |  | 6 | 3.02055 | 0.3596 | -0.1085 |
|  |  | 1 | 1.62257 | -1.1802 | -0.7158 |
|  |  | 1 | 1.70493 | -0.9214 | 1.04304 |
|  |  | 1 | 3.88007 | -0.3107 | -0.0519 |
|  |  | 1 | 3.02792 | 0.8559 | -1.0794 |
|  |  | 1 | 3.11518 | 1.11465 | 0.67299 |
|  |  | 7 | -1.7886 | 1.26526 | 0.00791 |
|  |  | 1 | -1.4913 | 2.06831 | 0.5399 |
|  |  | 1 | -2.4189 | 1.45139 | -0.756 |
|  |  |  |  |  |  |
|  |  |  |  |  |  |
| **10** | -366.392431 | 7 | 0.03262 | -0.1715 | -0.0193 |
|  |  | 6 | -0.0597 | 1.18789 | -0.1046 |
|  |  | 6 | 1.25109 | -0.807 | -0.5158 |
|  |  | 6 | 2.36269 | -0.8017 | 0.52822 |
|  |  | 1 | 1.57258 | -0.2697 | -1.4081 |
|  |  | 1 | 0.9958 | -1.8289 | -0.8082 |
|  |  | 1 | 3.25565 | -1.2984 | 0.143 |
|  |  | 1 | 2.61994 | 0.2276 | 0.78199 |
|  |  | 1 | 2.04794 | -1.3209 | 1.43707 |
|  |  | 6 | -0.9798 | -1.0322 | 0.57703 |
|  |  | 6 | -1.9233 | -1.6405 | -0.4582 |
|  |  | 1 | -1.5438 | -0.4707 | 1.32199 |
|  |  | 1 | -0.4589 | -1.8258 | 1.12157 |
|  |  | 1 | -2.639 | -2.3136 | 0.01882 |
|  |  | 1 | -2.4777 | -0.8577 | -0.9799 |
|  |  | 1 | -1.364 | -2.2093 | -1.2039 |
|  |  | 6 | -1.3292 | 1.83555 | 0.41634 |
|  |  | 1 | -2.2299 | 1.34851 | 0.03951 |
|  |  | 1 | -1.3541 | 1.80187 | 1.50825 |
|  |  | 1 | -1.3153 | 2.87525 | 0.09899 |
|  |  | 8 | 0.84146 | 1.86116 | -0.5742 |
|  |  |  |  |  |  |
|  |  |  |  |  |  |
| **11** | -879.517236 | 6 | 2.4044 | -0.6163 | -0.0559 |
|  |  | 15 | -0.1687 | 0.42861 | 0.07193 |
|  |  | 8 | 0.03775 | 1.49534 | -0.9036 |
|  |  | 9 | -0.3577 | 1.02142 | 1.53057 |
|  |  | 8 | -1.4388 | -0.5033 | -0.0473 |
|  |  | 6 | 2.90822 | 0.69469 | -0.5917 |
|  |  | 1 | 3.98183 | 0.60521 | -0.7334 |
|  |  | 1 | 2.41045 | 0.94068 | -1.5301 |
|  |  | 1 | 2.68686 | 1.50517 | 0.10585 |
|  |  | 6 | -2.7623 | 0.09085 | -0.0836 |
|  |  | 6 | -3.7405 | -1.005 | -0.4307 |
|  |  | 1 | -2.7605 | 0.88654 | -0.832 |
|  |  | 1 | -2.966 | 0.52182 | 0.89923 |
|  |  | 1 | -4.7523 | -0.5971 | -0.4599 |
|  |  | 1 | -3.5057 | -1.4287 | -1.4073 |
|  |  | 1 | -3.7056 | -1.7986 | 0.31632 |
|  |  | 8 | 3.09631 | -1.5783 | 0.14333 |
|  |  | 7 | 1.02536 | -0.7063 | 0.2295 |
|  |  | 1 | 0.76112 | -1.6081 | 0.61273 |
|  |  |  |  |  |  |
|  |  |  |  |  |  |
| **12** | -213.739689 | 7 | -0.0327 | -1.0436 | 0.12006 |
|  |  | 6 | 1.06769 | -0.2326 | 0.63523 |
|  |  | 6 | 1.77079 | 0.66984 | -0.3822 |
|  |  | 1 | 1.79193 | -0.9219 | 1.07642 |
|  |  | 1 | 0.68715 | 0.37482 | 1.46147 |
|  |  | 1 | 2.62848 | 1.16693 | 0.07673 |
|  |  | 1 | 2.13213 | 0.08771 | -1.2348 |
|  |  | 1 | 1.10285 | 1.44603 | -0.7629 |
|  |  | 6 | -1.0771 | -0.3121 | -0.594 |
|  |  | 6 | -1.7748 | 0.69475 | 0.31222 |
|  |  | 1 | -1.8063 | -1.0499 | -0.9376 |
|  |  | 1 | -0.7023 | 0.20646 | -1.4901 |
|  |  | 1 | -2.6414 | 1.12463 | -0.1934 |
|  |  | 1 | -2.1098 | 0.20519 | 1.229 |
|  |  | 1 | -1.1116 | 1.51847 | 0.58673 |
|  |  | 1 | 0.33773 | -1.7725 | -0.4797 |

**References**

[S1] A. R. H. Walker, R. D. Suenram, A. Samuels, J. Jensen, M. W. Ellzy, J. M. Lochner, and D. Zeroka, J. Mol. Spectrosc., 2001, 207, 77-82.

[S2] L. V. Skripnikov, Chemissian Version 4.23, Visualisation Computer Program, 2014, www.chemissian.com (Accessed 16 October 2018).

[S3] S. H. Lone, S. Jameel, M. A. Bhat, R. A. Lone, R. J. Butcherd, and K. A. Bhat, RSC Adv., 2018, 8, 8259-8268.

[S4] J. S. Murray and P. Politzer, WIREs Comput. Mol. Sci., 2011, 1, 153-163.

[S5] D. Majumdar, S. Roszak, J. Wang, T. C. Dinadayalane, B. Rasulev, H. Pinto, and J. Leszczynski, Advances in *In Silico* Research on Nerve Agents, J. Leszczynski and M.K. Shukla (Eds.), In: Practical Aspects of Computational Chemistry III, Springer, 2014, 283-322.

[S6] K. P. Huber and G. Herzberg, Molecular Spectra and Molecular Structure, IV. Constants of Diatomic Molecules, Van Nostrand Reinhold Co., 1979.

[S7] C. E. Moore, Atomic Energy Levels, Circular of the NBS 467, Washington, DC, Vol 1, 1949.

[S8] https://cccbdb.nist.gov/elecspin.asp [Accessed 21 August 2018]

[S9] J. D. Cox, D. D. Wagman, and V. A. Medvedev, CODATA Key Values for Thermodynamics, Hemisphere Publishing Corp., New York, 1989.

**Further reading**

[A] P. May, Novichok, The Notorious Nerve Agent, Molecule of the Month August 2018, http://www.chm.bris.ac.uk/motm/novichok/novichokh.htm; DOI:10.6084/m9.figshare.7177919 (Accessed 16 December 2018).

[B] C. H. Arnaud, C&EN’s Molecules of the Year for 2018, Chemistry News Outlet Highlights Compounds that Made Headlines this Year, 2018, 96 (4), https://cen.acs.org/synthesis/CENs-molecules-year-2018/96/i49#poll (Accessed 16 December 2018).
